# Modular geodesics and wedge domains in non-compactly causal symmetric spaces

**DOI:** 10.1007/s10455-023-09937-6

**Published:** 2023-12-31

**Authors:** Vincenzo Morinelli, Karl-Hermann Neeb, Gestur Ólafsson

**Affiliations:** 1 Dipartimento di Matematica, Università di Roma “Tor Vergata”, Rome, Italy; 2 Department Mathematik, Friedrich-Alexander-Universität, Erlangen-Nürnberg Cauerstrasse 11, 91058 Erlangen, Germany; 3 Department of mathematics, Louisiana State University, Baton Rouge, LA 70803 USA

**Keywords:** Euler element, Wedge domain, Causal space, Modular geodesic, Primary 22E45, Secondary 81R05, 81T05

## Abstract

We continue our investigation of the interplay between causal structures on symmetric spaces and geometric aspects of Algebraic Quantum Field Theory. We adopt the perspective that the geometric implementation of the modular group is given by the flow generated by an Euler element of the Lie algebra (an element defining a 3-grading). Since any Euler element of a semisimple Lie algebra specifies a canonical non-compactly causal symmetric space $M = G/H$, we turn in this paper to the geometry of this flow. Our main results concern the positivity region *W* of the flow (the corresponding wedge region): If *G* has trivial center, then *W* is connected, it coincides with the so-called observer domain, specified by a trajectory of the modular flow which at the same time is a causal geodesic. It can also be characterized in terms of a geometric KMS condition, and it has a natural structure of an equivariant fiber bundle over a Riemannian symmetric space that exhibits it as a real form of the crown domain of *G*/*K*. Among the tools that we need for these results are two observations of independent interest: a polar decomposition of the positivity domain and a convexity theorem for *G*-translates of open *H*-orbits in the minimal flag manifold specified by the 3-grading.

## Introduction

A new Lie theoretical approach to localization on spacetimes involved in Algebraic Quantum Field Theory (AQFT) has been introduced in the recent years by the authors and collaborators in a series of works, see [38, 41, 49–52, 54]. In the current paper, we continue the investigation of the structure of wedge regions in non-compactly causal symmetric spaces, started in [52]. First we briefly recall the motivation form AQFT, and then, we introduce tools and details to formulate our results.

Symmetric spaces are quotients $M = G/H$, where *G* is a Lie group, $\tau$ is an involutive automorphism of *G* and $H \subseteq G^\tau$ is an open subgroup (cf. [36]). A causal symmetric space carries a *G*-invariant field of pointed generating closed convex cones $C_m \subseteq T_m(M)$ in their tangent spaces. Typical examples are de Sitter space $\mathop {\textrm{dS}}\nolimits ^d \cong \mathop {\textrm{SO}}\nolimits _{1,d}({{\mathbb {R}}})_e/\mathop {\textrm{SO}}\nolimits _{1,d-1}({{\mathbb {R}}})_e$ and anti-de Sitter space $\mathop {\textrm{AdS}}\nolimits ^d \cong \mathop {\textrm{SO}}\nolimits _{2,d-1}({{\mathbb {R}}})_e/\mathop {\textrm{SO}}\nolimits _{1,d-1}({{\mathbb {R}}})_e$ as well as products $\mathop {\textrm{dS}}\nolimits ^d \times {{\mathbb {S}}}^k$ and $\mathop {\textrm{AdS}}\nolimits ^d \times {{\mathbb {H}}}^k$ with spheres and hyperbolic spaces, respectively. These are Lorentzian, but we do not require our causal structure to come from a Lorentzian metric, which creates much more flexibility and a richer variety of geometries. Causal symmetric spaces permit to study causality aspects of spacetimes in a highly symmetric environment. Here we shall always assume that *M* is *non-compactly causal* in the sense that the causal curves define a global order structure with compact order intervals (they are called globally hyperbolic), and in this context one can also prove the existence of a global “time function” with group theoretic methods (see [46]). We refer to the monograph [26] for more details and a complete exposition of the classification of irreducible causal symmetric spaces. A new perspective on the classification has been developed in [41].

Recent interest in causal symmetric spaces in relation to representation theory arose from their role as analogs of spacetime manifolds in the context of Algebraic Quantum Field Theory in the sense of Haag–Kastler. A model in AQFT is specified by a *net* of von Neumann algebras ${\mathcal {M}}({\mathcal {O}})$ acting on a fixed Hilbert space indexed by open subsets ${\mathcal {O}}$ of the chosen spacetime *M* [20]. The hermitian elements of the algebra ${\mathcal {M}}({\mathcal {O}})$ represent observables that can be measured in the “laboratory” ${\mathcal {O}}$. These nets are supposed to satisfy fundamental quantum and relativistic assumptions:

(I) Isotony: $${\mathcal {O}}_1 \subseteq {\mathcal {O}}_2$$ implies $${\mathcal {M}}({\mathcal {O}}_1) \subseteq {\mathcal {M}}({\mathcal {O}}_2).$$
(L) Locality: ${\mathcal {O}}_1 \subseteq {\mathcal {O}}_2'$ implies ${\mathcal {M}}({\mathcal {O}}_1) \subseteq {\mathcal {M}}({\mathcal {O}}_2)'$, where ${\mathcal {O}}'$ is the “causal complement” of ${\mathcal {O}}$, i.e., the maximal open subset that cannot be connected to ${\mathcal {O}}$ by causal curves.
(RS) Reeh–Schlieder property: There exists a unit vector $\Omega \in {\mathcal {H}}$ that is cyclic for ${\mathcal {M}}({\mathcal {O}})$ if ${\mathcal {O}}\not =\emptyset$.
(Cov) Covariance: There is a Lie group *G* acting on *M* and a unitary representation $U : G \rightarrow \mathop {\textrm{U}}\nolimits ({\mathcal {H}})$ such that $U_g {\mathcal {M}}({\mathcal {O}}) U_g^{-1} = {\mathcal {M}}(g{\mathcal {O}})$ for $g \in G$.
(BW) Bisognano–Wichmann property: $\Omega$ is separating for some “wedge region” $W \subseteq M$ and there exists an element $h \in {{\mathfrak {g}}}$ with $\Delta ^{-it/2\pi } = U(\exp th)$ for $t\in {{\mathbb {R}}}$, where $\Delta$ is the modular operator corresponding to $({\mathcal {M}}(W),\Omega )$ in the sense of the Tomita–Takesaki Theorem ([4, Thm. 2.5.14]).
(Vac) Invariance of the vacuum: $U(g)\Omega = \Omega$ for every $g \in G$.

The (BW) property gives a geometrical meaning to the dynamics provided by the modular group $(\Delta ^{it})_{t \in {{\mathbb {R}}}}$ of the von Neumann algebra ${\mathcal {M}}(W)$ associated with wedge regions with respect to the vacuum state specified by $\Omega$. On Minkowski/de Sitter spacetime, it provides an identification of the one-parameter group $(\Lambda _W(t))_{t \in {{\mathbb {R}}}}$ of boosts in the Poincaré/Lorentz group with the Tomita–Takesaki modular operator:

$$\begin{aligned} U(\Lambda _W(t))=\Delta ^{-it/2\pi }. \end{aligned}$$

Here $\Lambda _{W}=g\Lambda _{W_1}g^{-1}$ is a one-parameter group of boosts associated with $W = g.W_1$, where $W_1=\{x\in M:|x_0|<x_1\}$ is the standard right wedge and

$$\begin{aligned} \Lambda _{W_1}(t) =(\cosh (t)x_0+\sinh (t)x_1,\cosh (t)x_1+\sinh (t)x_0,x_2,\ldots ,x_d)\end{aligned}$$

describes the boosts associated with $W_1$.

The homogeneous spacetimes occurring naturally in AQFT are causal symmetric spaces associated with their symmetry groups (Minkowski spacetime for the Poincaré group, de Sitter space for the Lorentz group and anti-de Sitter space for $\mathop {\textrm{SO}}\nolimits _{2,d}({{\mathbb {R}}})$), and the localization in wedge regions is ruled by the acting group. The rich interplay between the geometric and algebraic objects in AQFT allowed a generalization of fundamental localization properties and the subsequent definition of fundamental models (second quantization fields), having as initial data a general Lie group with distinguished elements (Euler elements) in the Lie algebra. Given an AQFT on Minkowski spacetime $M = {{\mathbb {R}}}^{1,d}$ (or de Sitter spacetime $\mathop {\textrm{dS}}\nolimits ^d \subseteq {{\mathbb {R}}}^{1,d}$), the Bisognano–Wichmann (BW) property allows an identification of geometric and algebraic objects in both free and interacting theories in all dimensions [3, 13, 44]. This plays a central role in many results in AQFT and is a building block of our discussion.

One can generalize the picture we get from these explicit AQFT models and construct nets of von Neumann algebras on causal symmetric spaces with representation theoretical methods. We start with a unitary representation $U : G \rightarrow \mathop {\textrm{U}}\nolimits ({\mathcal {H}})$ of a reductive Lie group *G* whose Lie algebra contains Euler elements. Then, one constructs so-called one-particle nets on causal symmetric spaces. These are isotonous, *G*-covariant maps that associate to non-empty open subsets of the causal symmetric space standard subspaces^1^ of the “one-particle space” ${\mathcal {H}}$. For positive energy representations, we refer to [50] for left invariant nets on reductive Lie groups, to [55] for left invariant nets on non-reductive Lie groups, and to [51] for nets on compactly causal symmetric spaces. For general unitary representation, nets on non-compactly causal symmetric spaces have been constructed in [17] and on abstract wedge families in [38]; see also [40]. These constructions have the (BW) property as a fundamental input. Bosonic second quantization associates to a one-particle net an isotonous, *G*-covariant net of von Neumann algebras acting on the bosonic Fock space [5, 38].

These constructions naturally generalize the AQFT framework, re-construct the free second quantization AQFT models on the chiral conformal circle, on de Sitter and anti-de Sitter space, and provide several new models [17, 38, 50]. One can also recover free AQFT models on Minkowski spacetime as addressed in [38–40]. If *Z*(*G*) is non-trivial, then a proper second quantization scheme to provide a (twisted-)local net of von Neumann algebras remains to be determined (cf. [11, 19]). We stress that our setting provides a general framework to study properties of AQFT that is not restricted to second quantization theories. It also provides results on the type of von Neumann algebras and on properties of wedge symmetries appearing in these models (see, e.g., [40]).

We know from [40] that, in the general context, the potential generators $h \in {{\mathfrak {g}}}$ of the modular groups in (BW) are *Euler elements*, i.e., $\mathop {\textrm{ad}}\nolimits h$ defines a 3-grading

$$\begin{aligned} {{\mathfrak {g}}}= {{\mathfrak {g}}}_1(h) \oplus {{\mathfrak {g}}}_0(h) \oplus {{\mathfrak {g}}}_{-1}(h), \quad \text{ where } \quad {{\mathfrak {g}}}_\lambda (h) = \ker (\mathop {\textrm{ad}}\nolimits h - \lambda {{\textbf {1}}}). \end{aligned}$$

This leads to the question how the existence and the choice of the Euler element affect the geometry of the associated symmetric space. The (BW) property establishes a one-to-one correspondence between “wedge regions” $W \subseteq M$ and the associated Euler elements. So these *fundamental localization regions* can be determined in terms of Euler elements. This allowed the following generalization of nets of von Neumann algebras on Minkowski/de Sitter spacetime:

- Given a Lie group *G* with Lie algebra ${{\mathfrak {g}}}$, then the couples $(h,\tau _h)$, where $h \in {{\mathfrak {g}}}$ is an Euler element and $\tau _h$ an involutive automorphism of *G*, inducing on ${{\mathfrak {g}}}$ the involution $\tau _h = e^{\pi i \mathop {\textrm{ad}}\nolimits h}$, allow the definition of an ordered, *G*-covariant set of “abstract wedge regions” carrying also some locality information [38]. In particular, they encode the commutation relation property of the Tomita operators (modular operator and modular conjugation).
- Causal symmetric spaces provide manifolds and a causal structure supporting nets of algebras. Here the wedge regions can be defined as open subsets in several ways. The equivalence of various characterizations has been shown in [51, 52]; see also the discussion below.

The whole picture complies with Minkowski, de Sitter and anti-de Sitter spacetimes and the associated free fields. A generalization of wedge regions of the Minkowski or de Sitter spacetime on general curved spacetimes has been proposed by many authors, see for instance [12] and references therein. In our framework, on non-compactly causal symmetric spaces, the rich geometric symmetries allow different characterizations of wedge regions, in particular in terms of *positivity of the modular flow*, or *geometric KMS conditions* and in terms of *polar decompositions* as described in [52]. Some of them directly accord with the literature, for instance for positivity of the modular flow, see [9, Defin. 3.1] and in particular [45] for the connection to thermodynamics on de Sitter space. To see how these definitions apply to wedges in de Sitter space, cf. [52, App. D.3] and [6]. For causal symmetric spaces all definitions of wedge regions discussed in [41, 52] specify the same regions, up to choosing connected components (cf. [52, Thm. 7.1]). In Theorem 7.1, we prove that the identification is actually complete for the adjoint groups since the wedge region defined in terms of positivity of the modular flow is connected. This contrasts the situation for compactly causal symmetric spaces, where wedge regions are in general not connected, as for anti-de Sitter space ([52, Lemma 11.2]).

To formulate our results, we recall some basic terminology concerning symmetric Lie algebras (see [52] for more details).

- A *symmetric Lie algebra* is a pair $({{\mathfrak {g}}},\tau )$, where ${{\mathfrak {g}}}$ is a finite-dimensional real Lie algebra and $\tau$ an involutive automorphism of ${{\mathfrak {g}}}$. We write ${{\mathfrak {h}}}= {{\mathfrak {g}}}^\tau = \ker ({{\textbf {1}}} - \tau )$ and ${{\mathfrak {q}}}= {{\mathfrak {g}}}^{-\tau } = \ker ({{\textbf {1}}} + \tau )$ for the $\tau$-eigenspaces.
- A *causal symmetric Lie algebra* is a triple $({{\mathfrak {g}}},\tau ,C)$, where $({{\mathfrak {g}}},\tau )$ is a symmetric Lie algebra and $C \subseteq {{\mathfrak {q}}}$ is a pointed generating closed convex cone invariant under the group $\mathop {\textrm{Inn}}\nolimits _{{\mathfrak {g}}}({{\mathfrak {h}}}) = \langle e^{\mathop {\textrm{ad}}\nolimits {{\mathfrak {h}}}}\rangle$ acting in ${{\mathfrak {q}}}$. We call $({{\mathfrak {g}}},\tau ,C)$ *compactly causal (cc)* if *C* is *elliptic* in the sense that, for $x \in C^\circ$ (the interior of *C* in ${{\mathfrak {q}}}$), the operator $\mathop {\textrm{ad}}\nolimits x$ is semisimple with purely imaginary spectrum. We call $({{\mathfrak {g}}},\tau ,C)$ *non-compactly causal (ncc)* if *C* is *hyperbolic* in the sense that, for $x \in C^\circ$, the operator $\mathop {\textrm{ad}}\nolimits x$ is diagonalizable.

As explained in detail in [41], Euler elements in reductive Lie algebras ${{\mathfrak {g}}}$ lead naturally to ncc symmetric Lie algebras: For an Euler element $h \in {{\mathfrak {g}}}$, choose a Cartan involution $\theta$ of ${{\mathfrak {g}}}$ with ${{\mathfrak {z}}}({{\mathfrak {g}}}) \subseteq {{\mathfrak {g}}}^{-\theta }$ such that $\theta (h) = -h$. Then $\tau _h:= e^{\pi i \mathop {\textrm{ad}}\nolimits h}$ is an involutive automorphism of ${{\mathfrak {g}}}$ commuting with $\theta$, so that $\tau := \tau _h \theta$ defines a symmetric Lie algebra $({{\mathfrak {g}}},\tau )$ and there exists a pointed generating $\mathop {\textrm{Inn}}\nolimits _{{\mathfrak {g}}}({{\mathfrak {h}}})$-invariant hyperbolic cone *C* with $h \in C^\circ$. Under the assumption that ${{\mathfrak {h}}}= {{\mathfrak {g}}}^\tau$ contains no non-zero ideal of ${{\mathfrak {g}}}$, there is a unique minimal cone $C_{{\mathfrak {q}}}^{\textrm{min}}(h)$ with this property. It is generated by the orbit $\mathop {\textrm{Inn}}\nolimits _{{\mathfrak {g}}}({{\mathfrak {h}}})h \subseteq {{\mathfrak {q}}}$.

Let $({{\mathfrak {g}}},\tau ,C)$ be an ncc symmetric Lie algebra and $(G, \tau ^G, H)$ a corresponding *symmetric Lie group,* i.e., *G* is a connected Lie group, $\tau ^G$ an involutive automorphism of *G* integrating $\tau$, and $H \subseteq G^{\tau ^G}$ an open subgroup. If, in addition, $\mathop {\textrm{Ad}}\nolimits (H)C = C$, then we call the quadruple $(G,\tau ^G, H,C)$ a *causal symmetric Lie group*. On $M=G/H$, we then obtain the structure of a *causal symmetric space*, specified by the *G*-invariant field of open convex cones^2^

1.1 $$\begin{aligned} V_+(gH):= g.C^\circ \subseteq T_{gH}(M).^2 \end{aligned}$$

We further assume that

$$\begin{aligned} S:= H \exp (C) = \exp (C)H \subseteq G \end{aligned}$$

is a closed subsemigroup for which the polar map $H \times C \rightarrow S, (h,x) \mapsto h \exp x$ is a homeomorphism. Then

1.2 $$\begin{aligned} g_1 H \le g_2 H \quad \text{ if } \quad g_2^{-1} g_1 \in S \end{aligned}$$

defines on *M* a partial order, called the *causal order on* *M*. According to Lawson’s Theorem [30] and Theorem C.1), this is always the case if ${{\mathfrak {z}}}({{\mathfrak {g}}}) \subseteq {{\mathfrak {q}}}$ and $\exp \vert _{{{\mathfrak {z}}}({{\mathfrak {g}}})}$ is injective. The second condition is always satisfied if *G* is simply connected.

For an Euler element $h \in {{\mathfrak {g}}}$, we consider the associated *modular flow* on $M = G/H$, defined by

1.3 $$\begin{aligned} \alpha _t(gH) = \exp (th)gH. \end{aligned}$$

We study orbits of this flow which are geodesics $\gamma : {{\mathbb {R}}}\rightarrow M$ with respect to the symmetric space structure and causal in the sense that $\gamma '(t) \in V_+(\gamma (t))$ for $t \in {{\mathbb {R}}}$. We call them *h*-*modular geodesics*. All these are contained in the *positivity domain*

1.4 $$\begin{aligned} W_M^+(h):= \{ m \in M : X^M_h(m) \in V_+(m)\} \end{aligned}$$

of the vector field $X^M_h$ generating the modular flow. We refer to [52] for a detailed analysis of the latter domain in the special situations where the modular flow on *M* has fixed points, which is equivalent to the adjoint orbit ${\mathcal {O}}_h = \mathop {\textrm{Inn}}\nolimits ({{\mathfrak {g}}})h$ intersecting ${{\mathfrak {h}}}$.

We show for ncc symmetric Lie algebras, which are direct sums of irreducible ones, that:

- Causal modular geodesics exist if and only if the adjoint orbit ${\mathcal {O}}_h = \mathop {\textrm{Ad}}\nolimits (G)h\subseteq {{\mathfrak {g}}}$ intersects the interior of the cone $C \subseteq {{\mathfrak {q}}}$, and then the centralizer $G^h = \{ g \in G : \mathop {\textrm{Ad}}\nolimits (g)h = h\}$ of *h* acts transitively on the union of the corresponding curves (Proposition 3.2(c)).
- Suppose that the cone is maximal, i.e., $C_{{\mathfrak {q}}}= C_{{\mathfrak {q}}}^{\textrm{max}}$ (see (2.3) and [41, §3.5.2] for details). Let ${{\mathfrak {q}}}_{{\mathfrak {k}}}= {{\mathfrak {q}}}\cap {{\mathfrak {k}}}$ for a Cartan decomposition ${{\mathfrak {g}}}= {{\mathfrak {k}}}\oplus {{\mathfrak {p}}}$ with $h \in {{\mathfrak {q}}}_{{\mathfrak {p}}}$ and consider the domain
  $$\begin{aligned} \Omega _{{{\mathfrak {q}}}_{{\mathfrak {k}}}} = \Big \{ x \in {{\mathfrak {q}}}_{{\mathfrak {k}}}: \rho (\mathop {\textrm{ad}}\nolimits x) < \frac{\pi }{2}\Big \}, \end{aligned}$$
  where $\rho (\mathop {\textrm{ad}}\nolimits x)$ is the spectral radius of $\mathop {\textrm{ad}}\nolimits x$. Then, the connected component $W:= W_M^+(h)_{eH}$ of the base point *eH* in the positivity domain is
  $$\begin{aligned} W = G^h_e.\mathop {\textrm{Exp}}\nolimits _{eH}(\Omega _{{{\mathfrak {q}}}_{{\mathfrak {k}}}}) \end{aligned}$$
  (Theorem 3.6).
- We associate to any modular geodesic a connected open subset $W(\gamma ) \subseteq M$; the corresponding *observer domain*. For de Sitter space $\mathop {\textrm{dS}}\nolimits ^d$, we thus obtain the familiar wedge domain obtained by intersecting $\mathop {\textrm{dS}}\nolimits ^d$ with a Rindler wedge in Minkowski space ${{\mathbb {R}}}^{1,d}$ (Example 5.3). In Theorem 5.7, we show that it coincides with *W*, provided that $H = K^h \exp ({{\mathfrak {h}}}_{{\mathfrak {p}}})$ and $C_{{\mathfrak {q}}}= C_{{\mathfrak {q}}}^{\textrm{max}}$.
- A key step in the proof of Theorem 5.7 is the following Convexity Theorem. Let
  $$\begin{aligned} P^-:= \exp ({{\mathfrak {g}}}_{-1}(h)) G^h \subseteq G \end{aligned}$$
  be the “negative” parabolic subgroup of *G* specified by *h* and identity ${{\mathfrak {g}}}_1(h)$ with the open subset ${\mathcal {B}}:= \exp ({{\mathfrak {g}}}_1(h)).eP^- \subseteq G/P^-$. Then ${\mathcal {D}}:= H.0 \subseteq {\mathcal {B}}$ is an open convex subset, and for any $g \in G$ with $g.{\mathcal {D}}\subseteq {\mathcal {B}}$, the subset $g.{\mathcal {D}}\subseteq {\mathcal {B}}$ is convex (Theorem 4.5).
- In Sect. 6 we further show that, for $C= C_{{\mathfrak {q}}}^{\textrm{max}}$ and ${{\mathfrak {g}}}$ simple, that the real tube domain ${{\mathfrak {h}}}+ C^\circ$ intersects the set ${\mathcal {E}}({{\mathfrak {g}}})$ of Euler elements in a connected subset (Theorem 6.1). As a consequence, we derive that $W_M^+(h') \not =\emptyset$ if and only if $h' \in {\mathcal {O}}_h$ (Corollary 6.3). In particular, only one conjugacy class of Euler elements possesses non-empty positivity regions. This is of particular relevance for locality properties of nets of local algebras. We plan to investigate this in subsequent work.
- In Theorem 7.1 we show that the positivity domain $W_M^+(h)$ is connected for $G = \mathop {\textrm{Inn}}\nolimits ({{\mathfrak {g}}})$ and ${{\mathfrak {g}}}$ simple, and this implies that
  $$\begin{aligned} W(\gamma ) = W = W_M^+(h).\end{aligned}$$
  From this in turn we derive that the stabilizer group $G_W = \{ g \in G : g.W = W\}$ coincides with $G^h$ (Proposition 7.3), so that the *wedge space* ${\mathcal {W}}(M):= \{g.W : g \in G\}$ of wedge regions in *M* can be identified, as a homogeneous *G*-space, with the adjoint orbit ${\mathcal {O}}_h= \mathop {\textrm{Ad}}\nolimits (G)h \cong G/G^h$. In particular ${\mathcal {W}}(M)$ also is a symmetric space.
- Finally, we show in Theorem 8.2 that *W* coincides with the KMS wedge domain
  $$\begin{aligned} W^{\textrm{KMS}} = \{ m \in M : \alpha _{it}(m) \in \Xi \text{ for } 0< t < \pi \}, \end{aligned}$$
  where $\Xi$ is the crown domain of the Riemannian symmetric space *G*/*K*.

We conclude this introduction with some more motivation from AQFT. The analysis of the properties of the modular flow on symmetric spaces is also motivated by the investigation of energy inequalities in quantum and relativistic theories. In General Relativity, there exist many solutions to the Einstein equation that, for various reasons, may not be physical. Energy conditions such as the pointwise non-negativity of the energy density, which ensures that the gravity force is attractive, can be required to discard non-physical models [14, 62]. In quantum and relativistic theories, the energy conditions need to be rewritten. For instance, it is well known that the energy density at individual spacetime points is unbounded from below, even if the energy density integrated over a Cauchy surface is non-negative (see [14, 15] and references therein).

Families of inequalities have been discussed in several models, employing different mathematical and physical approaches (see for instance [14, 16, 27, 29, 42, 61]). In recent years, operator algebraic techniques have been very fruitful for the study of the energy inequalities because of the central role played by the modular hamiltonian in some of these energy conditions. This object corresponds to the logarithm of the modular operator of a local algebra of a specific region, which in some cases can be identified with the generator of a one-parameter group of spacetime symmetries by the Bisognano–Wichmann property. In this regard, we mention the ANEC (Averaged Null Energy Condition) and the QNEC (Quantum Null Energy Condition) and their relation with the Araki relative entropy, an important quantum-information quantity, defined in terms of relative modular operators (see, for instance, [1, 9, 10, 32–35, 43]). We stress that, in this analysis, the study of the modular flow on the manifold can be particularly relevant. Moreover, in order to find regions where energy inequalities hold, one may also need to deform the modular flow [8, 43]. In our abstract context, the Euler element specifies the flow that can be implemented by the modular operator, hence the modular Hamiltonian, when the Bisognano–Wichmann property holds. In particular, the identification of specific flows on symmetric spaces (modular flows), the characterization in terms of modular operators of covariant local subspaces attached to specific regions (wedges) motivate an analysis of modular flows on non-compactly causal symmetric spaces pursued in our project.

In this respect, the wedge regions are the first fundamental open subsets of spacetime to be studied in detail. Following General Relativity (see, for instance, [9, 12] and references therein), one can define them as an open connected, causally convex subregion *W* of a spacetime *M*, associated with a Killing flow $\Lambda$ preserving *W*, which is timelike and time-oriented on *W*. On Minkowski spacetime the flow $\Lambda$, a one-parameter group of boosts, corresponds to the time-evolution of a uniformly accelerated observer moving within *W*. Then, *W* is a horizon for this observer: he cannot send a signal outside *W* and receive it back. Then the vacuum state becomes a thermal state for the algebra of observables inside the wedge region *W* by the Bisognano–Wichmann property [18, 21, 31]. In our general context, we recover the definition (and equivalent ones) of wedge regions. Then, by the Bisognano–Wichmann property, the thermal property of the vacuum state holds when nets of algebras or standard subspaces are considered [38, 41, 52]. In this paper, we focus on the related properties of the wedge regions in non-compactly causal symmetric spaces.

### Notation

- If *M* is a topological space and $m\in M$, then $M_m$ denotes the connected component of *M* containing *m*. In particular, we write $e \in G$ for the identity element in the Lie group *G* and $G_e$ for its identity component.
- Involutive automorphisms of *G* are typically denoted $\tau ^G$, and $\tau$ is the corresponding automorphism of the Lie algebra ${{\mathfrak {g}}}= \mathop {{\textbf {L}}}\nolimits (G)$. We write ${{\mathfrak {g}}}^\tau = \ker ({{\textbf {1}}}-\tau )$ and ${{\mathfrak {g}}}^{-\tau } = \ker ({{\textbf {1}}} + \tau )$.
- For $x \in {{\mathfrak {g}}}$, we write $G^x:= \{ g \in G : \mathop {\textrm{Ad}}\nolimits (g)x = x \}$ for the stabilizer of *x* in the adjoint representation and $G^x_e = (G^x)_e$ for its identity component.
- For $h \in {{\mathfrak {g}}}$ and $\lambda \in {{\mathbb {R}}}$, we write ${{\mathfrak {g}}}_\lambda (h):= \ker (\mathop {\textrm{ad}}\nolimits h - \lambda {{\textbf {1}}})$ for the corresponding eigenspace in the adjoint representation.
- If ${{\mathfrak {g}}}$ is a Lie algebra, we write ${\mathcal {E}}({{\mathfrak {g}}})$ for the set of *Euler elements* $h \in {{\mathfrak {g}}}$, i.e., $\mathop {\textrm{ad}}\nolimits h$ is non-zero and diagonalizable with $\textrm{Spec}(\mathop {\textrm{ad}}\nolimits h) \subseteq \{-1,0,1\}$. The corresponding involution is denoted $\tau _h = e^{\pi i \mathop {\textrm{ad}}\nolimits h}$.
- For a Lie subalgebra ${{\mathfrak {s}}}\subseteq {{\mathfrak {g}}}$, we write $\mathop {\textrm{Inn}}\nolimits _{{\mathfrak {g}}}({{\mathfrak {s}}})= \langle e^{\mathop {\textrm{ad}}\nolimits {{\mathfrak {s}}}} \rangle \subseteq \mathop {\textrm{Aut}}\nolimits ({{\mathfrak {g}}})$ for the subgroup generated by $e^{\mathop {\textrm{ad}}\nolimits {{\mathfrak {s}}}}$.
- For a convex cone *C* in a vector space *V*, we write $C^\circ := \mathop {\textrm{int}}\nolimits _{C-C}(C)$ for the relative interior of *C* in its span.
- We use the notation
  1.5 $$\begin{aligned} \rho (A):= \sup \{ |\lambda | : \lambda \in \textrm{Spec}(A) \} \end{aligned}$$
  for the spectral radius of a linear operator *A*.

## Causal Euler elements and ncc symmetric spaces

In this section, we recall some basic results on Euler elements and their relation with non-compactly causal symmetric spaces. Most of these statements are discussed in detail in [41].

Recall from above that an Euler element in a Lie algebra ${{\mathfrak {g}}}$ is an element *h* defining a 3-grading of ${{\mathfrak {g}}}$ by ${{\mathfrak {g}}}= {{\mathfrak {g}}}_{-1}\oplus {{\mathfrak {g}}}_0\oplus {{\mathfrak {g}}}_{+1}$ with ${{\mathfrak {g}}}_j = \ker (\mathop {\textrm{ad}}\nolimits h - j{{\textbf {1}}})$, $j =-1,0,1$. We write ${\mathcal {E}}({{\mathfrak {g}}})$ for the set of Euler elements in ${{\mathfrak {g}}}$. In this section, we recall some results on from [41] on Euler elements that are crucially used in the following.

### Definition 2.1

Let ${{\mathfrak {g}}}$ be a reductive Lie algebra.

- A *Cartan involution* of ${{\mathfrak {g}}}$ is an involutive automorphism $\theta$ for which ${{\mathfrak {z}}}({{\mathfrak {g}}}) \subseteq {{\mathfrak {g}}}^{-\theta }$ and ${{\mathfrak {g}}}^\theta$ is maximal compactly embedded in the commutator algebra $[{{\mathfrak {g}}},{{\mathfrak {g}}}]$. We then write, using the notation from the introduction,
  $$\begin{aligned} {{\mathfrak {g}}}= {{\mathfrak {k}}}\oplus {{\mathfrak {p}}}\quad \text{ with } \quad {{\mathfrak {k}}}= {{\mathfrak {g}}}^\theta \quad \text{ and } \quad {{\mathfrak {p}}}= {{\mathfrak {g}}}^{-\theta } \end{aligned}$$
- If $\tau$ is another involution on ${{\mathfrak {g}}}$ commuting with $\theta$, ${{\mathfrak {h}}}:= {{\mathfrak {g}}}^\tau$ and ${{\mathfrak {q}}}:= {{\mathfrak {g}}}^{-\tau }$, then we have
  $$\begin{aligned} {{\mathfrak {h}}}= {{\mathfrak {h}}}_{{\mathfrak {k}}}\oplus {{\mathfrak {h}}}_{{\mathfrak {p}}}, \quad {{\mathfrak {q}}}= {{\mathfrak {q}}}_{{\mathfrak {k}}}\oplus {{\mathfrak {q}}}_{{\mathfrak {p}}}\quad \text{ with } \quad {{\mathfrak {h}}}_{{\mathfrak {k}}}= {{\mathfrak {h}}}\cap {{\mathfrak {k}}}, \quad {{\mathfrak {h}}}_{{\mathfrak {p}}}= {{\mathfrak {h}}}\cap {{\mathfrak {p}}},\quad {{\mathfrak {q}}}_{{\mathfrak {k}}}= {{\mathfrak {q}}}\cap {{\mathfrak {k}}}, \quad {{\mathfrak {q}}}_{{\mathfrak {p}}}= {{\mathfrak {q}}}\cap {{\mathfrak {p}}}.\end{aligned}$$
- The *Cartan dual* of the symmetric Lie algebra $({{\mathfrak {g}}},\tau )$ is the symmetric Lie algebra $({{\mathfrak {g}}}^c, \tau ^c)$ with
  $$\begin{aligned} {{\mathfrak {g}}}^c = {{\mathfrak {h}}}+ i {{\mathfrak {q}}}\quad \text{ and } \quad \tau ^c(x+ iy) = x - iy \quad \text{ for } \quad x \in {{\mathfrak {h}}}, y \in {{\mathfrak {q}}}.\end{aligned}$$
  Note that ${{\mathfrak {g}}}^c =({{\mathfrak {g}}}_{{\mathbb {C}}})^{\overline{\tau }}$ where $\overline{\tau }$ is the conjugate-linear extension of $\tau$ to ${{\mathfrak {g}}}_{{\mathbb {C}}}$; in particular ${{\mathfrak {g}}}^c$ is a real form of ${{\mathfrak {g}}}_{{\mathbb {C}}}$.

### Definition 2.2

Let $({{\mathfrak {g}}},\tau )$ be a symmetric Lie algebra and $h \in {\mathcal {E}}({{\mathfrak {g}}}) \cap {{\mathfrak {q}}}$. We say that *h* is *causal* if there exists an $\mathop {\textrm{Inn}}\nolimits _{{\mathfrak {g}}}({{\mathfrak {h}}})$-invariant closed pointed generating convex cone *C* in ${{\mathfrak {q}}}$ with $h\in C^\circ$. We write ${\mathcal {E}}_c({{\mathfrak {q}}}) \subseteq {\mathcal {E}}({{\mathfrak {g}}}) \cap {{\mathfrak {q}}}$ for the *set of causal Euler elements in* ${{\mathfrak {q}}}$. Recall that the triple $({{\mathfrak {g}}},\tau ,C)$ is ncc if *C* is hyperbolic.

### Lemma 2.3

Let $({{\mathfrak {g}}},\tau ,C)$ be a **simple** ncc symmetric Lie algebra and $h \in {{\mathfrak {q}}}$ be a causal Euler element. Then, the following assertions hold:

- There exist closed convex pointed generating $\mathop {\textrm{Inn}}\nolimits _{{\mathfrak {g}}}({{\mathfrak {h}}})$-invariant cones
  $$\begin{aligned} C_{{\mathfrak {q}}}^{\textrm{min}}(h) \subseteq C_{{\mathfrak {q}}}^{\textrm{max}}(h) \end{aligned}$$
  such that $h \in C_{{\mathfrak {q}}}^{\textrm{min}}(h)^\circ$ and either
  $$\begin{aligned} C_{{\mathfrak {q}}}^{\textrm{min}}(h) \subseteq C \subseteq C_{{\mathfrak {q}}}^{\textrm{max}}(h) \quad \text{ or } \quad C_{{\mathfrak {q}}}^{\textrm{min}}(h) \subseteq -C \subseteq C_{{\mathfrak {q}}}^{\textrm{max}}(h).\end{aligned}$$
- If $(G,\tau ^G, H)$ is a connected symmetric Lie group with symmetric Lie algebra $({{\mathfrak {g}}},\tau )$, then two mutually exclusive cases occur:
  $\mathop {\textrm{Ad}}\nolimits (H)h = \mathop {\textrm{Ad}}\nolimits (H_e)h$ and *G*/*H* is causal.
  $-h \in \mathop {\textrm{Ad}}\nolimits (H)h$ and *G*/*H* is not causal.

### Proof

(a) follows from [41, Sect. 3.5.2] and (b) from [41, Prop. 4.18]. $\square$

If *h* is an Euler element in the **reductive** Lie algebra ${{\mathfrak {g}}}$ and $\theta$ a Cartan involution with $\theta (h) = -h$, $\tau := \theta \tau _h$ and ${{\mathfrak {z}}}({{\mathfrak {g}}}) \subseteq {{\mathfrak {g}}}^{-\theta }$, then [41, Thm. 4.2] implies that there exists an $\mathop {\textrm{Inn}}\nolimits _{{\mathfrak {g}}}({{\mathfrak {h}}})$-invariant pointed closed convex cone $C \subseteq {{\mathfrak {q}}}$ with $h\in C^\circ$, so that $({{\mathfrak {g}}}, \tau ,C)$ is ncc. Further, all ideals of ${{\mathfrak {g}}}$ contained in ${{\mathfrak {g}}}^\tau = {{\mathfrak {h}}}$ are compact. We have a decomposition

2.1 $$\begin{aligned} {{\mathfrak {g}}}= {{\mathfrak {g}}}_k \oplus {{\mathfrak {g}}}_r \oplus {{\mathfrak {g}}}_s, \end{aligned}$$

where ${{\mathfrak {g}}}_s$ is the sum of all simple ideals not commuting with *h* (the strictly ncc part), ${{\mathfrak {g}}}_r$ is the sum of the center ${{\mathfrak {z}}}({{\mathfrak {g}}})$ and all non-compact simple ideals commuting with *h* on which $\tau = \theta$ (the non-compact Riemannian part), and ${{\mathfrak {g}}}_k$ is the sum of all simple compact ideals (they commute with *h*). All these ideals are invariant under $\theta$ and $\tau = \tau _h \theta$, so that we obtain decompositions

2.2 $$\begin{aligned} {{\mathfrak {g}}}_s = {{\mathfrak {h}}}_s\oplus {{\mathfrak {q}}}_s, \quad {{\mathfrak {g}}}_r = {{\mathfrak {h}}}_r\oplus {{\mathfrak {q}}}_r \quad \text{ and } \quad {{\mathfrak {g}}}_k = {{\mathfrak {h}}}_k, \end{aligned}$$

where ${{\mathfrak {h}}}_r \oplus {{\mathfrak {h}}}_k$ is a compact ideal of ${{\mathfrak {h}}}$, ${{\mathfrak {g}}}_r = {{\mathfrak {h}}}_r\oplus {{\mathfrak {q}}}_r$ is a Cartan decomposition and ${{\mathfrak {q}}}_{{\mathfrak {p}}}={{\mathfrak {q}}}_{{{\mathfrak {p}}},s}\oplus {{\mathfrak {q}}}_r$. In particular ${{\mathfrak {q}}}= {{\mathfrak {q}}}_s \oplus {{\mathfrak {q}}}_r$. Let $p_s: {{\mathfrak {q}}}\rightarrow {{\mathfrak {q}}}_s$ be the projection onto ${{\mathfrak {q}}}_s$ with kernel ${{\mathfrak {q}}}_r$. Then, [41, Prop. B.4] implies that every $\mathop {\textrm{Inn}}\nolimits _{{\mathfrak {g}}}({{\mathfrak {h}}})$-invariant closed convex cone *C* satisfies

$$\begin{aligned} C_s:= p_s(C) = C\cap {{\mathfrak {q}}}_s\quad \text {and}\quad C_s^\circ = C^\circ \cap {{\mathfrak {q}}}_s. \end{aligned}$$

By Lemma 2.3(a), we obtain a pointed $\mathop {\textrm{Inn}}\nolimits _{{\mathfrak {g}}}({{\mathfrak {h}}})$-invariant cone $C_{{{\mathfrak {q}}}_s}^{\textrm{min}}(h) \subseteq {{\mathfrak {q}}}_s$, adapted to the decomposition into irreducible summands, whose dual cone $C_{{{\mathfrak {q}}}_s}^{\textrm{max}}(h)$ with respect to the Cartan–Killing form $\kappa (x,y) = \mathop {\textrm{tr}}\nolimits (\mathop {\textrm{ad}}\nolimits x \mathop {\textrm{ad}}\nolimits y)$ satisfies $C_{{{\mathfrak {q}}}_s}^\textrm{min}(h) \subseteq C_{{{\mathfrak {q}}}_s}^{\textrm{max}}(h)$. Put

2.3 $$\begin{aligned} C_{{\mathfrak {q}}}^{\textrm{min}}(h):= C_{{{\mathfrak {q}}}_s}^{\textrm{min}}(h) \subseteq C_{{\mathfrak {q}}}^{\textrm{max}}(h):= C_{{{\mathfrak {q}}}_s}^{\textrm{max}}(h) + {{\mathfrak {q}}}_r. \end{aligned}$$

Both cones are adapted to the decomposition of $({{\mathfrak {g}}},\tau )$ into irreducible summands. Further, each pointed generating $\mathop {\textrm{Inn}}\nolimits _{{\mathfrak {g}}}({{\mathfrak {h}}})$-invariant cone *C* containing *h* satisfies

2.4 $$\begin{aligned} C_{{\mathfrak {q}}}^{\textrm{min}}(h) \subseteq C \subseteq C_{{\mathfrak {q}}}^\textrm{max}(h). \end{aligned}$$

Here the first inclusion is obvious, and the second one follows from the fact that *h* is also contained in the dual cone

$$\begin{aligned} C^{\star } = \{ y \in {{\mathfrak {q}}}: (\forall x \in C) \ \kappa (x,y) \ge 0\}.\end{aligned}$$

This leads to $C_{{\mathfrak {q}}}^{\textrm{min}}(h) \subseteq C^\star$, and thus to $C \subseteq C_{{\mathfrak {q}}}^{\textrm{min}}(h)^\star = C_{{\mathfrak {q}}}^{\textrm{max}}(h)$ (cf. [41, §3.5] for more details).

### Lemma 2.4

If $x \in (C_{{\mathfrak {q}}}^{\textrm{max}})^\circ$, then the centralizer ${{\mathfrak {z}}}_{{\mathfrak {h}}}(x) = {{\mathfrak {h}}}\cap \ker (\mathop {\textrm{ad}}\nolimits x)$ is compactly embedded in ${{\mathfrak {g}}}$, i.e., consists of elliptic elements.

### Proof

First we observe that the cone $C_{{\mathfrak {q}}}^{\textrm{max}}$ is adapted to the decomposition ${{\mathfrak {g}}}= ({{\mathfrak {g}}}_k + {{\mathfrak {g}}}_r) + {{\mathfrak {g}}}_s$ and so is the centralizer of $x = x_r + x_s$ in ${{\mathfrak {h}}}= ({{\mathfrak {g}}}_{{\mathfrak {k}}}+ {{\mathfrak {k}}}_r) + {{\mathfrak {h}}}_s$. Hence the assertion follows from the fact that ${{\mathfrak {g}}}_k + {{\mathfrak {k}}}_r$ is compactly embedded and ${{\mathfrak {z}}}_{{{\mathfrak {h}}}_s}(x) = {{\mathfrak {z}}}_{{{\mathfrak {h}}}_s}(x_s)$ is compactly embedded because the cone $C_{{{\mathfrak {q}}}_s}^{\textrm{max}}$ is pointed ([47, Prop. V.5.11]). $\square$

### Theorem 2.5

(Uniqueness of the causal involution) ([41, Thm. 4.5]) Let $({{\mathfrak {g}}},\tau ,C)$ be a semisimple ncc symmetric Lie algebra for which all ideals of ${{\mathfrak {g}}}$ contained in ${{\mathfrak {h}}}$ are compact, ${{\mathfrak {g}}}_s$ the sum of all non-Riemannian ideals, ${{\mathfrak {q}}}_s:= {{\mathfrak {g}}}_s \cap {{\mathfrak {q}}}$, $C_s:= C \cap {{\mathfrak {q}}}_s$, and $\theta$ a Cartan involution commuting with $\tau$. Then the following assertions hold:

- $C_s^\circ \cap {{\mathfrak {q}}}_{{\mathfrak {p}}}$ contains a unique Euler element *h*, and this Euler element satisfies $\tau = \tau _h \theta$.
- $\mathop {\textrm{Inn}}\nolimits _{{\mathfrak {g}}}({{\mathfrak {h}}})$ acts transitively on $C_s^\circ \cap {\mathcal {E}}({{\mathfrak {g}}})$.
- For every Euler element $h \in C_s^\circ$, the involution $\tau \tau _h$ is Cartan.

### Proposition 2.6

Let $(G,\tau ^G, H,C)$ be a connected semisimple ncc symmetric Lie group for which ${{\mathfrak {h}}}= {{\mathfrak {g}}}^\tau$ contains no non-compact ideal of ${{\mathfrak {g}}}$ (${{\mathfrak {g}}}= {{\mathfrak {g}}}_r + {{\mathfrak {g}}}_s$) and let $h \in C_s^\circ$ (cf. Theorem 2.5) be a causal Euler element. Then the following assertions hold:

- $H = H_e H^h$, i.e., every connected component of *H* meets $H^h$.
- $\mathop {\textrm{Ad}}\nolimits (H^h) = \mathop {\textrm{Ad}}\nolimits (H)^h$ is a maximal compact subgroup of $\mathop {\textrm{Ad}}\nolimits (H)$.
- $\mathop {\textrm{Ad}}\nolimits (H)^h = \mathop {\textrm{Ad}}\nolimits (H)^{\tau _h}$ and $\tau _h:= e^{\pi i \mathop {\textrm{ad}}\nolimits h}$ induces a Cartan involution on $\mathop {\textrm{Ad}}\nolimits (H)$.
- $\tau$ induces a Cartan involution on $\mathop {\textrm{Ad}}\nolimits (H)^h$ for which $\mathop {\textrm{Ad}}\nolimits (H^h_e)^\tau = e^{\mathop {\textrm{ad}}\nolimits {{\mathfrak {h}}}_{{\mathfrak {k}}}}$ is connected.

### Proof

The statements on the adjoint group $\mathop {\textrm{Ad}}\nolimits (G) = \mathop {\textrm{Inn}}\nolimits ({{\mathfrak {g}}})$ follow from [41, Cor. 4.6] because $\mathop {\textrm{Ad}}\nolimits (H) \subseteq \mathop {\textrm{Inn}}\nolimits ({{\mathfrak {g}}})^\tau$ preserves *C*. Further, $\mathop {\textrm{Ad}}\nolimits (H)^h = \mathop {\textrm{Ad}}\nolimits (H^h)$ and $H^h = \mathop {\textrm{Ad}}\nolimits ^{-1}(\mathop {\textrm{Ad}}\nolimits (H)^h)$ imply with (a) (for $\mathop {\textrm{Ad}}\nolimits (G)$) that $H = H_e H^h$. $\square$

### Definition 2.7

If ${{\mathfrak {g}}}$ is a simple hermitian Lie algebra, $\theta$ a Cartan involution of ${{\mathfrak {g}}}$ and ${{\mathfrak {a}}}\subseteq {{\mathfrak {p}}}$ maximal abelian, then the restricted root system $\Sigma ({{\mathfrak {g}}},{{\mathfrak {a}}})$ is either of type $C_r$ or $BC_r$. In the first case, we say that ${{\mathfrak {g}}}$ is *of tube type*.

Recall that if $({{\mathfrak {g}}},\tau )$ is simple ncc, then either ${{\mathfrak {g}}}^c$ is simple hermitian or ${{\mathfrak {g}}}^c \cong {{\mathfrak {h}}}_{{\mathbb {C}}}$, where ${{\mathfrak {h}}}= {{\mathfrak {g}}}^\tau$ is simple hermitian ([41, Rem. 4.24]).

### Proposition 2.8

([41, Lemma 5.1, Prop. 5.2]) Let $({{\mathfrak {g}}},\tau ,C)$ be a simple ncc symmetric Lie algebra. Pick a causal Euler element $h \in C^\circ$ and ${{\mathfrak {t}}}_{{\mathfrak {q}}}\subseteq {{\mathfrak {q}}}_{{\mathfrak {k}}}$ maximal abelian and set $s:= \mathop {\textrm{dim}}\nolimits {{\mathfrak {t}}}_{{\mathfrak {q}}}$. Then, the following assertions hold:

- The Lie algebra ${{\mathfrak {l}}}$ generated by *h* and ${{\mathfrak {t}}}_{{\mathfrak {q}}}$ is reductive.
- The commutator algebra $[{{\mathfrak {l}}},{{\mathfrak {l}}}]$ is isomorphic to $\mathop {{\mathfrak {sl} }}\nolimits _2({{\mathbb {R}}})^s$
- ${{\mathfrak {z}}}({{\mathfrak {l}}}) = {{\mathbb {R}}}h_0$ for some hyperbolic element $h_0$ satisfying $\tau (h_0) =- h_0 = \theta (h_0)$ which is zero if and only if ${{\mathfrak {g}}}^c$ is of tube type.
- The Lie algebra ${{\mathfrak {l}}}$ is $\tau$-invariant and ${{\mathfrak {l}}}^\tau \cong \mathop {{\mathfrak {so} }}\nolimits _{1,1}({{\mathbb {R}}})^s$.
- For $x \in {{\mathfrak {t}}}_{{\mathfrak {q}}}$, we have $\rho (\mathop {\textrm{ad}}\nolimits x) = \rho (\mathop {\textrm{ad}}\nolimits x\vert _{{{\mathfrak {s}}}}),$ where $\rho$ denotes the spectral radius. With the basis
  $$\begin{aligned} z^j = \Big (0,\ldots , 0, \frac{1}{2}\begin{pmatrix} 0 &{}\quad -1 \\ 1 &{}\quad 0 \end{pmatrix},0,\cdots , 0\Big ), \quad j =1,\ldots , s, \end{aligned}$$
  in $\mathop {{\mathfrak {so} }}\nolimits _2({{\mathbb {R}}})^s$ we have for $x = \sum _{j = 1}^s x_j z^j$
  2.5 $$\begin{aligned} \rho (\mathop {\textrm{ad}}\nolimits x) = \max \{ |x_j| : j = 1,\ldots , s\}. \end{aligned}$$

Note that (c) implies that ${{\mathfrak {l}}}$ is semisimple, i.e., $h \in [{{\mathfrak {l}}},{{\mathfrak {l}}}]$, if and only if ${{\mathfrak {g}}}^c$ is of tube type.

### Proposition 2.9

([41, Prop. 7.10]) Let $({{\mathfrak {g}}},\tau ,C)$ be a semisimple modular non-compactly causal semisimple symmetric Lie algebra, where $\tau = \tau _h \theta$, $h \in {{\mathfrak {q}}}_{{\mathfrak {p}}}\cap C$ a causal Euler element,

$$\begin{aligned} G:= \mathop {\textrm{Inn}}\nolimits _{{{\mathfrak {g}}}_{{\mathbb {C}}}}({{\mathfrak {g}}})\cong \mathop {\textrm{Inn}}\nolimits ({{\mathfrak {g}}}), \quad K:= G^\theta = \mathop {\textrm{Inn}}\nolimits _{{\mathfrak {g}}}({{\mathfrak {k}}}),\quad \text{ and } \quad G^c:= \mathop {\textrm{Inn}}\nolimits _{{{\mathfrak {g}}}_{{\mathbb {C}}}}({{\mathfrak {g}}}^c).\end{aligned}$$

Then $$H:= G \cap G^c$$ satisfies

$$\begin{aligned} H = K^h \exp ({{\mathfrak {h}}}_{{\mathfrak {p}}}) \quad \text{ and } \quad H \cap K = K^h.\end{aligned}$$

In particular $K^h \subseteq K^{\tau _h} = K^\tau$ implies $H \subseteq G^\tau$.

## The positivity domain and modular geodesics

Let $(G, \tau ^G, H, C)$ be a connected **semisimple** causal symmetric Lie group with ncc symmetric Lie algebra $({{\mathfrak {g}}},\tau , C)$. We fix a causal Euler element $h \in C^\circ$ (Theorem 2.5) and write $M = G/H$ for the associated symmetric space.

One of our goals in this paper is to describe the structure of the *positivity domain*

$$\begin{aligned} W_M^+(h):= \{ m \in M : X^M_h(m) \in V_+(m)\} \end{aligned}$$

of the vector field $X^M_h$ generating the modular flow. Our first major result is the identification of the connected component *W* of the base point *eH* in the positivity domain $W_M^+(h)$ as

3.1 $$\begin{aligned} W:= W_M^+(h)_{eH} = G^h_e \mathop {\textrm{Exp}}\nolimits _{eH}(\Omega _{{{\mathfrak {q}}}_{{\mathfrak {k}}}}) \end{aligned}$$

(Theorem 3.6).

Some of the results in this section had been obtained in [52] for the special case of ncc symmetric Lie algebras for which ${{\mathfrak {h}}}$ contains an Euler element, whereas here we are dealing with general non-compactly causal symmetric Lie algebras.

### Modular geodesics

In this subsection, we introduce the concept of an *h*-modular geodesic in a non-compactly causal symmetric space *M* and discuss some of its immediate properties. We also show that, in compactly causal spaces, non-trivial causal modular geodesics do not exist.

#### Definition 3.1

*(Geodesics and causality)* Let $M = G/H$ as above.

- We call a geodesic $\gamma : {{\mathbb {R}}}\rightarrow M$ *causal* if $\gamma '(t) \in V_+(\gamma (t))$ for every $t \in {{\mathbb {R}}}$ (see (1.1)).
- Let $h \in {{\mathfrak {g}}}$ be an Euler element. The flow on *M* defined by
  3.2 $$\begin{aligned} \alpha _t(gH) = \exp (th) gH =g\exp (\mathop {\textrm{Ad}}\nolimits (g^{-1})h)H \end{aligned}$$
  is called the *modular flow* (associated to *h*). Its infinitesimal generator is denoted $X^M_h \in {\mathcal {V}}(M)$.
- A geodesic $\gamma : {{\mathbb {R}}}\rightarrow M$ is called *h*-*modular* if $\gamma (t) = \alpha _t(\gamma (0))$ holds for all $t \in {{\mathbb {R}}}$, i.e., $\gamma$ is an integral curve of $X_h^M$.

#### Proposition 3.2

Suppose that $({{\mathfrak {g}}},\tau )$ is a direct sum of irreducible ncc symmetric Lie algebras (${{\mathfrak {g}}}= {{\mathfrak {g}}}_s$). The following assertions hold for any Euler element ${h} \in {\mathcal {E}}({{\mathfrak {g}}})$ and the corresponding modular flow $\alpha _t(m) = \exp (t{h}).m$ on $M = G/H$:

- The orbit under the modular flow is a causal geodesic if and only if *m* is contained in
  3.3 $$\begin{aligned} M^{{h}}_C = \{ g H\in G/H : \mathop {\textrm{Ad}}\nolimits (g)^{-1} {h} \in C^\circ \}. \end{aligned}$$
- All connected components of $M^{{h}}_C$ are Riemannian symmetric space of non-compact type: For every $m \in M^{{h}}_C$, the exponential map
  $$\begin{aligned} \mathop {\textrm{Exp}}\nolimits _m : T_m(M_C^{{h}}) \rightarrow (M^{{h}})_m \end{aligned}$$
  is a diffeomorphism.
- *h*-modular causal geodesics exist if and only if ${\mathcal {O}}_{{h}} = \mathop {\textrm{Ad}}\nolimits (G){h}$ intersects $C^\circ$. In this case $G^{{h}}$ acts transitively on $M_C^{{h}}$.

#### Proof

(a) Assume first that $\mathop {\textrm{Ad}}\nolimits (g)^{-1} h\in {{\mathfrak {q}}}$. Then (3.2) implies that the orbit of $m = gH$ under the modular flow is a geodesic. The causality is by definition equivalent to $\mathop {\textrm{Ad}}\nolimits (g)^{-1}{h}\in C^\circ$.

Suppose, conversely, that $t \mapsto \alpha _t(gH)$ is a causal geodesic. Lemma B.1 implies that $\mathop {\textrm{Ad}}\nolimits (g)^{-1}{h} = x_{{\mathfrak {h}}}+ x_{{\mathfrak {q}}}$, where $[x_{{\mathfrak {h}}}, x_{{\mathfrak {q}}}] = 0$ and $x_{{\mathfrak {q}}}\in C^\circ$. By Lemma 2.4, $x_{{\mathfrak {h}}}$ is elliptic and $x_{{\mathfrak {q}}}$ is hyperbolic because it is contained in $C^\circ$. Therefore, $\mathop {\textrm{ad}}\nolimits x_{{\mathfrak {h}}}+ \mathop {\textrm{ad}}\nolimits x_{{\mathfrak {q}}}$ is the unique Jordan decomposition of $\mathop {\textrm{ad}}\nolimits x$ into elliptic and hyperbolic summand. As $\mathop {\textrm{Ad}}\nolimits (g)^{-1}{h}$ is an Euler element, the elliptic summand vanishes, and thus, $\mathop {\textrm{ad}}\nolimits x_{{\mathfrak {h}}}= 0$, i.e., $x_{{\mathfrak {h}}}\in {{\mathfrak {z}}}({{\mathfrak {g}}}) \cap {{\mathfrak {h}}}= \{0\}$ (recall that ${{\mathfrak {z}}}({{\mathfrak {g}}}) \subseteq {{\mathfrak {q}}}$). This shows that $\mathop {\textrm{Ad}}\nolimits (g)^{-1} {h} \in {{\mathfrak {q}}}$, so that $gH \in M^{{h}}_C$.

(b) Choosing *m* as a base point, we may assume that $m = eH$, so that (a) implies that $h \in C^\circ \subseteq {{\mathfrak {q}}}$ is a causal Euler element. Pick a Cartan involution $\theta$ commuting $\tau$ which satisfies $\theta (h) = -h$ (cf. [28]), i.e., $h \in {{\mathfrak {q}}}_{{\mathfrak {p}}}$. Then $\tau = \tau _{h} \theta$ follows from Theorem 2.5(a). As $(M^c_C)_m = G^h_e.m$ by Lemma B.2, the assertion now follows from ${{\mathfrak {g}}}^h ={{\mathfrak {h}}}_{{\mathfrak {k}}}\oplus {{\mathfrak {q}}}_{{\mathfrak {p}}}$.

(c) The first assertion follows immediately from (a). For the second assertion, suppose that $m_0 = g_0H \in M^{{h}}_C$. As $M^{{h}}_C$ is $G^{{h}}$-invariant, $G^{{h}}.m_0 \subseteq M^{{h}}_C$. Let $h_c:= \mathop {\textrm{Ad}}\nolimits (g_0)^{-1}{h}$, so that ${\mathcal {E}}({{\mathfrak {g}}}) \cap C^\circ = \mathop {\textrm{Inn}}\nolimits _{{\mathfrak {g}}}({{\mathfrak {h}}}) h_c$ by Theorem 2.5(b) (recall that $C = C_s$). If $gH \in M^h_C$, i.e.,

$$\begin{aligned} \mathop {\textrm{Ad}}\nolimits (g)^{-1} {{h}} \in C^\circ \cap {\mathcal {E}}({{\mathfrak {g}}}) \ {\buildrel 2.5 \over =}\ \mathop {\textrm{Inn}}\nolimits _{{\mathfrak {g}}}({{\mathfrak {h}}}) h_c = \mathop {\textrm{Inn}}\nolimits _{{\mathfrak {g}}}({{\mathfrak {h}}})\mathop {\textrm{Ad}}\nolimits (g_0)^{-1}h, \end{aligned}$$

then there exists an element $g_1 \in \mathop {\textrm{Inn}}\nolimits _{{\mathfrak {g}}}({{\mathfrak {h}}})$ with $g g_1 g_0^{-1} \in G^{{h}}$, so that $g \in G^{{h}} g_0 g_1^{-1} \in G^{{h}} g_0 H$, and therefore $gH \in G^{{h}}.m_0$. $\square$

We record the following consequence of (3.2):

#### Lemma 3.3

For any causal Euler element $h \in C^\circ$, we have

$$\begin{aligned} W_M^+(h) = \{ gH \in G/H : \mathop {\textrm{Ad}}\nolimits (g)^{-1}h \in {\mathcal {T}}_C\}, \quad \text{ where } \quad {\mathcal {T}}_C:= {{\mathfrak {h}}}+ C^\circ .\end{aligned}$$

Due to the hyperbolicity of Euler elements, modular causal geodesics do not exist for compactly causal symmetric spaces:

#### Proposition 3.4

If $M = G/H$ is a compactly causal symmetric space, then non-trivial causal modular geodesics do not exist.

#### Proof

If there exists a modular causal geodesic and $({{\mathfrak {g}}},\tau ,C)$ is the infinitesimal data of *M*, then there exists a $g \in G$ such that the Euler element *h* satisfies $\mathop {\textrm{Ad}}\nolimits (g)^{-1}h = x_{{\mathfrak {h}}}+ x_{{\mathfrak {q}}}$ with $x_{{\mathfrak {q}}}\in C^\circ$ and $[x_{{\mathfrak {h}}}, x_{{\mathfrak {q}}}] =0$ (Lemma B.1). As *C* is elliptic, $x_{{\mathfrak {q}}}$ is elliptic. Further the pointedness of *C* implies that $x_{{\mathfrak {h}}}\in \ker (\mathop {\textrm{ad}}\nolimits x_{{\mathfrak {q}}})$ is elliptic. This implies that the Euler element $\mathop {\textrm{Ad}}\nolimits (g)^{-1}h$ is elliptic, a contradiction. $\square$

### The fiber bundle structure of the positivity domain

The main result of this section is Theorem 3.6 in which we exhibit a natural bundle structure on the wedge domain $W \subseteq M$ that is equivariant with respect to the connected group $G^h_e$, the base is the Riemannian symmetric space of this group, and the fiber is a bounded convex subset of ${{\mathfrak {q}}}_{{\mathfrak {k}}}$.

#### Definition 3.5

Let $h \in {{\mathfrak {q}}}_{{\mathfrak {p}}}\cap C^\circ$ be a causal Euler element, so that $\tau = \tau _h \theta$. Then ${{\mathfrak {z}}}_{{\mathfrak {h}}}(h) = {{\mathfrak {h}}}^{\tau _h} = {{\mathfrak {h}}}_{{\mathfrak {k}}}$ implies that

$$\begin{aligned}{\mathcal {O}}_h^{{\mathfrak {q}}}:= \mathop {\textrm{Inn}}\nolimits _{{\mathfrak {g}}}({{\mathfrak {h}}})h = e^{\mathop {\textrm{ad}}\nolimits {{\mathfrak {h}}}_{{\mathfrak {p}}}}h\end{aligned}$$

is the non-compact Riemannian symmetric space associated with the symmetric Lie algebra $({{\mathfrak {h}}},\theta )$.

#### Theorem 3.6

(Positivity Domain Theorem) Suppose that $(G,\tau ^G,C,H)$ is a connected semisimple non-compactly causal Lie group for which $({{\mathfrak {g}}},\tau )$ contains no $\tau$-invariant Riemannian ideals (${{\mathfrak {g}}}= {{\mathfrak {g}}}_s$) and that *h* is a causal Euler element. Suppose that $C:= C^{\textrm{max}}_{{\mathfrak {q}}}(h)$ is the maximal $\mathop {\textrm{Inn}}\nolimits _{{\mathfrak {g}}}({{\mathfrak {h}}})$-invariant cone with $h \in C^\circ$. Then, the following assertions hold:

- The connected component $W= W_M^+(h)_{eH}$ of *eH* in the positivity domain $W_M^+(h)$ is given by
  3.4 $$\begin{aligned} W = G^h_e.\mathop {\textrm{Exp}}\nolimits _{eH}(\Omega _{{{\mathfrak {q}}}_{{\mathfrak {k}}}}), \quad \text{ where } \quad \Omega _{{{\mathfrak {q}}}_{{\mathfrak {k}}}} = \Big \{ x \in {{\mathfrak {q}}}_{{\mathfrak {k}}}: \rho (\mathop {\textrm{ad}}\nolimits x) < \frac{\pi }{2}\Big \}. \end{aligned}$$
- The polar map $\Psi : G^h_e \times _{G^h_e \cap H} \Omega _{{{\mathfrak {q}}}_{{\mathfrak {k}}}} \rightarrow W, [g,x] \mapsto g.\mathop {\textrm{Exp}}\nolimits _{eH}(x)$ is a diffeomorphism
- *W* is contractible, hence in particular simply connected.
- $$G^h_e \cap H = K^h_e$$.

#### Proof

(a) Recall from [41, Thm. 6.7] that the connected component of *h* in the open subset ${\mathcal {O}}_{h} \cap {\mathcal {T}}_{C}$ of ${\mathcal {O}}_{h}$ is

3.5 $$\begin{aligned} \mathop {\textrm{Inn}}\nolimits _{{\mathfrak {g}}}({{\mathfrak {h}}})e^{\mathop {\textrm{ad}}\nolimits \Omega _{{{\mathfrak {q}}}_{{\mathfrak {k}}}}} h = \mathop {\textrm{Ad}}\nolimits (H_e)e^{\mathop {\textrm{ad}}\nolimits \Omega _{{{\mathfrak {q}}}_{{\mathfrak {k}}}}} h \subseteq {\mathcal {T}}_{C}. \end{aligned}$$

If $x \in \Omega _{{{\mathfrak {q}}}_{{\mathfrak {k}}}}$, then $\rho (\mathop {\textrm{ad}}\nolimits x) < \pi /2$, so that (3.5) implies that $g = \exp x$ satisfies

3.6 $$\begin{aligned} \mathop {\textrm{Ad}}\nolimits (g)^{-1} h = e^{-\mathop {\textrm{ad}}\nolimits x} h \in {\mathcal {T}}_{C} = {{\mathfrak {h}}}+ C^\circ . \end{aligned}$$

By Lemma 3.3

3.7 $$\begin{aligned} \mathop {\textrm{Exp}}\nolimits _{eH}(\Omega _{{{\mathfrak {q}}}_{{\mathfrak {k}}}})\subseteq W_M^+(h), \quad \text{ and } \text{ thus } \quad G^h_e.\mathop {\textrm{Exp}}\nolimits _{eH}(\Omega _{{{\mathfrak {q}}}_{{\mathfrak {k}}}})\subseteq W \end{aligned}$$

by $G^h$-invariance of $W_M^+(h)$.

Conversely, for $gH \in W$, the element $\mathop {\textrm{Ad}}\nolimits (g)^{-1}h \in {\mathcal {O}}_h \cap {\mathcal {T}}_{C}$ is contained in the connected component of *h*, so that (3.5) implies that it is contained in $\mathop {\textrm{Ad}}\nolimits (H_e) e^{\mathop {\textrm{ad}}\nolimits \Omega _{{{\mathfrak {q}}}_{{\mathfrak {k}}}}} h$. Therefore

$$\begin{aligned} g H_e \exp (\Omega _{{{\mathfrak {q}}}_{{\mathfrak {k}}}}) \cap G^h \not =\emptyset .\end{aligned}$$

This is equivalent to $g H_e \cap G^h \exp (\Omega _{{{\mathfrak {q}}}_{{\mathfrak {k}}}}) \not =\emptyset ,$ which implies

$$\begin{aligned} gH \in G^h \exp (\Omega _{{{\mathfrak {q}}}_{{\mathfrak {k}}}}).eH = G^h \mathop {\textrm{Exp}}\nolimits _{eH}(\Omega _{{{\mathfrak {q}}}_{{\mathfrak {k}}}})\end{aligned}$$

and thus

3.8 $$\begin{aligned} W \subseteq G^h.\mathop {\textrm{Exp}}\nolimits _{eH}(\Omega _{{{\mathfrak {q}}}_{{\mathfrak {k}}}}). \end{aligned}$$

If $g \in G^h$ satisfies $g \mathop {\textrm{Exp}}\nolimits _{eH}(\Omega _{{{\mathfrak {q}}}_{{\mathfrak {k}}}}) \cap W \not = \emptyset ,$ then $g.W = W$ follows from (3.7) and the fact that *g* permutes the connected components of $W_M^+(h)$. Therefore, (3.8), combined with (3.7), leads with $G^h_W:= \{ g \in G^h : g.W = W\}$ to

$$\begin{aligned} W \subseteq G^h_W.\mathop {\textrm{Exp}}\nolimits _{eH}(\Omega _{{{\mathfrak {q}}}_{{\mathfrak {k}}}}) \subseteq G^h_W.W = W,\end{aligned}$$

and this entails

3.9 $$\begin{aligned} W = G^h_W.\mathop {\textrm{Exp}}\nolimits _{eH}(\Omega _{{{\mathfrak {q}}}_{{\mathfrak {k}}}}). \end{aligned}$$

Next we observe that the exponential map $\mathop {\textrm{Exp}}\nolimits _{eH} : {{\mathfrak {q}}}_{{\mathfrak {k}}}\rightarrow M$ is regular in every $x\in \Omega _{{{\mathfrak {q}}}_{{\mathfrak {k}}}}$ because $\rho (\mathop {\textrm{ad}}\nolimits x)< \pi /2 < \pi$ ([52, Lemma C.3(b)]). Thus [52, loc.cit.] further implies that the map

$$\begin{aligned} \Phi : G^h \times \Omega _{{{\mathfrak {q}}}_{{\mathfrak {k}}}} \rightarrow M, \quad (g,x) \mapsto g.\mathop {\textrm{Exp}}\nolimits _{eH}(x) \end{aligned}$$

is regular in (*g*, *x*) because $\textrm{Spec}(\mathop {\textrm{ad}}\nolimits x) \subseteq (-\pi /2,\pi /2)i$ does not intersect $\big (\frac{\pi }{2} + {{\mathbb {Z}}}\pi \big )i$ for $x \in \Omega _{{{\mathfrak {q}}}_{{\mathfrak {k}}}}$. This implies that the differential of $\Phi$ is surjective in each point of $G^h \times \Omega _{{{\mathfrak {q}}}_{{\mathfrak {k}}}}$; hence, the image of every connected component is open. Now the connectedness of *W* implies that $W \subseteq G^h_e.\mathop {\textrm{Exp}}\nolimits _{eH}(\Omega _{{{\mathfrak {q}}}_{{\mathfrak {k}}}}),$ and this completes the proof.

(b)–(d): The surjectivity of $\Psi$ follows from Theorem 3.6. As ${{\mathfrak {g}}}^h = {{\mathfrak {h}}}_{{\mathfrak {k}}}\oplus {{\mathfrak {q}}}_{{\mathfrak {p}}}$ is a Cartan decomposition of ${{\mathfrak {g}}}^h$, the polar map $K^h_e \times {{\mathfrak {q}}}_{{\mathfrak {p}}}\rightarrow G^h_e, (k,x) \mapsto k \exp x$ is a diffeomorphism. In particular,

$$\begin{aligned} G^h_e \cap H \subseteq G^h_e \cap G^{\tau ^G} = K^h_e \subseteq H \end{aligned}$$

implies $G^h_e \cap H = K^h_e$ and thus (b).

The space $G^h_e \times _{G^h_e \cap H} \Omega _{{{\mathfrak {q}}}_{{\mathfrak {k}}}}$ is a fiber bundle over $G^h_e/K^h_e$ whose fiber is the convex set $\Omega _{{{\mathfrak {q}}}_{{\mathfrak {k}}}}$. Therefore, it is homotopy equivalent to the base $G^h_e/K^h_e$, which is also contractible because the exponential map $\mathop {\textrm{Exp}}\nolimits _{eH} : {{\mathfrak {q}}}_{{\mathfrak {p}}}\rightarrow G^h_e/K^h_e$ is a diffeomorphism.

It therefore suffices to show that $\Psi$ is a diffeomorphism. The proof of (a) shows already that its differential is everywhere surjective, hence invertible by equality of the dimensions of both spaces. So it suffices to check injectivity, i.e., that $\mathop {\textrm{Exp}}\nolimits := \mathop {\textrm{Exp}}\nolimits _{eH} : {{\mathfrak {q}}}\rightarrow M$ satisfies

3.10 $$\begin{aligned} g_1.\mathop {\textrm{Exp}}\nolimits (x_1) = g_2.\mathop {\textrm{Exp}}\nolimits (x_2) \quad \Rightarrow \quad g_2^{-1}g_1 \in K^h_e, \quad x_2 = \mathop {\textrm{Ad}}\nolimits (g_2^{-1}g_1)x_1. \end{aligned}$$

**Step 1:** $\mathop {\textrm{Exp}}\nolimits \vert _{\Omega _{{{\mathfrak {q}}}_{{\mathfrak {k}}}}}$ is injective. If $\mathop {\textrm{Exp}}\nolimits (x_1) = \mathop {\textrm{Exp}}\nolimits (x_2)$, then applying the quadratic representation implies $\exp (2 x_1) = \exp (2 x_2)$ in *G*. As $x_1$ and $x_2$ are both $\exp$-regular, [25, Lemma 9.2.31] implies that

$$\begin{aligned}{}[x_1, x_2]= 0 \quad \text{ and } \quad \exp (2x_1 - 2 x_2)= e.\end{aligned}$$

We conclude that $e^{2 \mathop {\textrm{ad}}\nolimits (x_1-x_2)} = \mathop {\textrm{id}}\nolimits _{{\mathfrak {g}}}$, and since the spectral radius of $2 \mathop {\textrm{ad}}\nolimits (x_1 - x_2)$ is less than $2 \pi$, it follows that $\mathop {\textrm{ad}}\nolimits (x_1 - x_2) = 0$, so that $x_1 = x_2$.

**Step 2:** $g.\mathop {\textrm{Exp}}\nolimits (x_1) = \mathop {\textrm{Exp}}\nolimits (x_2)$ with $g \in G^h_e$ and $x_1, x_2 \in \Omega _{{{\mathfrak {q}}}_{{\mathfrak {k}}}}$ implies $g \in K^h_e$. Applying the involution $\theta ^M$, we see that $g.\mathop {\textrm{Exp}}\nolimits (x_1)$ is a fixed point, so that

$$\begin{aligned} g.\mathop {\textrm{Exp}}\nolimits (x_1) = \theta (g).\mathop {\textrm{Exp}}\nolimits (x_1) \end{aligned}$$

entails that $\theta (g)^{-1}g$ fixes $m_1:=\mathop {\textrm{Exp}}\nolimits (x_1)$. We now write $g = k \exp z$ in terms of the polar decomposition of $G^h_e$ and obtain

$$\begin{aligned} \theta (g)^{-1} g = \exp (2z) \in G^{m_1}.\end{aligned}$$

Applying the quadratic representation, we get

3.11 $$\begin{aligned} \exp (2z) \exp (2x_1) \exp (2z) = \exp (2x_1), \end{aligned}$$

which can be rewritten as

$$\begin{aligned} \exp (e^{2\mathop {\textrm{ad}}\nolimits x_1} 2z) = \exp (-2z).\end{aligned}$$

Since $\mathop {\textrm{ad}}\nolimits z$ has real spectrum, so has $e^{2 \mathop {\textrm{ad}}\nolimits x_1} z$. Therefore the same arguments as in Step 1 above imply that

$$\begin{aligned}{}[z,e^{2 \mathop {\textrm{ad}}\nolimits x_1} z] = 0 \quad \text{ and } \quad \exp (2 e^{2 \mathop {\textrm{ad}}\nolimits x_1} z + 2z) = e,\end{aligned}$$

and $e^{2 \mathop {\textrm{ad}}\nolimits x_1} z = - z$. The vanishing ${{\mathfrak {h}}}$-component of this element is $\sinh (2 \mathop {\textrm{ad}}\nolimits x_1) z$, and since ${\rho (2\mathop {\textrm{ad}}\nolimits x_1) < \pi }$, it follows that $[x_1, z] =0$. Now (3.11) leads to $\exp (4z) = e$, and further to $z = 0$, because the exponential function on ${{\mathfrak {q}}}_{{\mathfrak {p}}}$ is injective. This proves that $g = k \in K^h_e$.

**Step 3:** From (3.10), we derive

$$\begin{aligned} g_2^{-1}g_1.\mathop {\textrm{Exp}}\nolimits (x_1) = \mathop {\textrm{Exp}}\nolimits (x_2),\end{aligned}$$

so that Step 2 shows that $k:= g_2^{-1}g_1 \in K^h_e$. We thus obtain

$$\begin{aligned} \mathop {\textrm{Exp}}\nolimits (x_2) = k.\mathop {\textrm{Exp}}\nolimits (x_1) = \mathop {\textrm{Exp}}\nolimits (\mathop {\textrm{Ad}}\nolimits (k) x_1),\end{aligned}$$

and since $\mathop {\textrm{Ad}}\nolimits (k) x_1 \in \Omega _{{{\mathfrak {q}}}_{{\mathfrak {k}}}}$, we infer from Step 1 that $\mathop {\textrm{Ad}}\nolimits (k) x_1 = x_2$. This completes the proof. $\square$

The following corollary identifies the connected component of $M^h_C$ containing *eH* as a submanifold (cf. Lemma B.2) of the wedge domain *W*.

#### Corollary 6.4

Assume that $\tau _h^G$ exists and leaves *H* invariant, so that $\tau _h^M$ exists and leaves the base point $eH \in M$ invariant. Then $\tau ^M_h(W) = W$ and the fixed point set of $\tau _h^M$ in *W* is the Riemannian symmetric space

$$\begin{aligned} W^{\tau _h^M} = M^h_{eH} = G^h_e.eH = \mathop {\textrm{Exp}}\nolimits _{eH}({{\mathfrak {q}}}_{{\mathfrak {p}}}). \end{aligned}$$

#### Proof

For $$g \in G^h$$ and $$x\in {{\mathfrak {q}}}_{{\mathfrak {k}}}$$:

$$\begin{aligned} \tau _h^M(g \mathop {\textrm{Exp}}\nolimits _{eH}(x)) = g \mathop {\textrm{Exp}}\nolimits _{eH}(\tau _h(x)) = g \mathop {\textrm{Exp}}\nolimits _{eH}(\tau (x)) = g \mathop {\textrm{Exp}}\nolimits _{eH}(-x).\end{aligned}$$

So $g \mathop {\textrm{Exp}}\nolimits _{eH}(x)$ is a fixed point if and only if $\mathop {\textrm{Exp}}\nolimits _{eH}(-x) = \mathop {\textrm{Exp}}\nolimits _{eH}(x),$ which is equivalent to $\exp (2x) \in H$. Now $\tau (x) = -x$ implies $\exp (2x) =\exp (-2x)$. As $\rho (2\mathop {\textrm{ad}}\nolimits x) < \pi$, [52, Lemma C.3] further shows that $x-(-x) = 2x \in {{\mathfrak {z}}}({{\mathfrak {g}}}) = \{0\}$. Therefore, $g \mathop {\textrm{Exp}}\nolimits _{eH}(x)$ is a fixed point if and only if $x = 0$.

From $W = G^h_e.\mathop {\textrm{Exp}}\nolimits _{eH}(\Omega _{{{\mathfrak {q}}}_{{\mathfrak {k}}}})$ and the polar decomposition $G^h_e = K^h_e \exp ({{\mathfrak {q}}}_{{\mathfrak {p}}}) = \exp ({{\mathfrak {q}}}_{{\mathfrak {p}}}) (H_K)_e$ (Theorem3.6(b)), we derive that the fixed point set is

$$\begin{aligned} W^{\tau _h^M} = G^h_e.eH = \mathop {\textrm{Exp}}\nolimits _{eH}({{\mathfrak {q}}}_{{\mathfrak {p}}}) = M^h_{eH}.\end{aligned}$$

The preceding corollary shows that the wedge domain $W \subseteq M = G/H$ contains the symmetric subspace $M^h_{eH} = \mathop {\textrm{Exp}}\nolimits _{eH}({{\mathfrak {q}}}_{{\mathfrak {p}}})$ as the fixed point set of an involution. Hence, the description of *W* from Theorem 3.6 as

$$\begin{aligned} W = G^h_e.\mathop {\textrm{Exp}}\nolimits _{eH}(\Omega _{{{\mathfrak {q}}}_{{\mathfrak {k}}}}) \end{aligned}$$

suggest to consider *W* as a real “crown domain” of the Riemannian symmetric space $M^h_{eH} \cong G^h/H^h$.

#### Remark 3.8

Theorem 3.6 has a trivial generalization to semisimple non-compactly causal Lie algebras of the form ${{\mathfrak {g}}}= {{\mathfrak {g}}}_k \oplus {{\mathfrak {g}}}_r \oplus {{\mathfrak {g}}}_s$ because then

$$\begin{aligned} C_{{\mathfrak {q}}}^{\textrm{max}} = {{\mathfrak {q}}}_r \oplus C_{{{\mathfrak {q}}}_s}^{\textrm{max}} \quad \text{ and } \quad {\mathcal {T}}_{C_{{\mathfrak {q}}}^{\textrm{max}}} = {{\mathfrak {g}}}_k + {{\mathfrak {g}}}_r + {\mathcal {T}}_{C_{{{\mathfrak {q}}}_s}^{\textrm{max}}}.\end{aligned}$$

For $h = h_r + h_s$ with $h_s \in C_{{{\mathfrak {q}}}_s}^\circ$ the relation $\mathop {\textrm{Ad}}\nolimits (g)^{-1}h \in {\mathcal {T}}_{C_{{\mathfrak {q}}}^\textrm{max}}$ is therefore equivalent to $\mathop {\textrm{Ad}}\nolimits (g)^{-1}h_s \in {\mathcal {T}}_{C_{{{\mathfrak {q}}}_s}^{\textrm{max}}}$. If $M = \mathop {\textrm{Inn}}\nolimits ({{\mathfrak {g}}})/\mathop {\textrm{Inn}}\nolimits ({{\mathfrak {g}}})^\tau \cong M_r \times M_s$ is the corresponding product decomposition, we obtain

$$\begin{aligned} W_M^+(h) = M_r \times W_{M_s}^+(h_s) \quad \text{ for } \quad C = C_{{{\mathfrak {q}}}}^{\textrm{max}}.\end{aligned}$$

However, if ${{\mathfrak {g}}}_r \not =\{0\}$, then $C_{{\mathfrak {q}}}^{\textrm{max}}$ is not pointed, and there are many pointed invariant cones *C*, which are not maximal, for which the domain $W_M^+(h)$ may have a more complicated structure.

#### Example 3.9

We consider the reductive Lie algebra

$$\begin{aligned} {{\mathfrak {g}}}= \mathop {{{\mathfrak {gl}} }}\nolimits _2({{\mathbb {R}}}) = {{\mathbb {R}}}{{\textbf {1}}} \oplus \mathop {{\mathfrak {sl} }}\nolimits _2({{\mathbb {R}}}).\end{aligned}$$

Any Euler element in ${{\mathfrak {g}}}$ is conjugate to some

$$\begin{aligned} h = \begin{pmatrix} \lambda &{}\quad 0 \\ 0 &{}\quad \mu \end{pmatrix}\quad \text{ with } \quad \lambda - \mu = 1.\end{aligned}$$

The Cartan involution $\theta (x) = -x^\top$ on ${{\mathfrak {g}}}$ then satisfies $\theta (h) = -h$ and $\tau := \theta \tau _h$ acts by

$$\begin{aligned} \tau \begin{pmatrix} a &{}\quad b \\ c &{}\quad d \end{pmatrix} = \begin{pmatrix} -a &{}\quad c \\ b &{}\quad -d \end{pmatrix}.\end{aligned}$$

With the Euler element

$$\begin{aligned} h_0:= \frac{1}{2}\begin{pmatrix} 0 &{}\quad 1 \\ 1 &{}\quad 0 \end{pmatrix}, \end{aligned}$$

we then have

$$\begin{aligned} {{\mathfrak {h}}}= {{\mathbb {R}}}h_0 = \mathop {{\mathfrak {so} }}\nolimits _{1,1}({{\mathbb {R}}}) \quad \text{ and } \quad {{\mathfrak {q}}}= {{\mathbb {R}}}{{\textbf {1}}} + \underbrace{{{\mathbb {R}}}h + {{\mathbb {R}}}z}_{{{\mathfrak {q}}}_s} \quad \text{ with } \quad z:= \frac{1}{2} \begin{pmatrix} 0 &{}\quad -1 \\ 1 &{}\quad 0 \end{pmatrix}. \end{aligned}$$

The group $G:= \mathop {\textrm{GL}}\nolimits _2({{\mathbb {R}}})_e$ acts by $g.A:= g A g^\top$ on the 3-dimensional space $\mathop {\textrm{Sym}}\nolimits _2({{\mathbb {R}}})$ of symmetric matrices and the stabilizer of $$I_{1,-1}:= \begin{pmatrix} 1 &{}\quad 0 \\ 0 &{}\quad -1 \end{pmatrix}$$ is the subgroup $H:= \mathop {\textrm{SO}}\nolimits _{1,1}({{\mathbb {R}}})$ with Lie algebra ${{\mathfrak {h}}}$. Therefore $M:= G.I_{1,1} \cong G/H$ can be identified with the subspace $\mathop {\textrm{Sym}}\nolimits _{1,1}({{\mathbb {R}}})$ of indefinite symmetric matrices. Note that ${{\mathbb {R}}}^\times _e{{\textbf {1}}} = Z(G)_e$ acts by multiplication with $\lambda ^2$ and that ${{\mathbb {R}}}^\times _+ \times M_1 \rightarrow M, (\lambda ,A) \mapsto \lambda A$ is a diffeomorphism, where

$$\begin{aligned} M_1:= \{ A \in M : \det (A) =-1\} \cong \mathop {\textrm{SL}}\nolimits _2({{\mathbb {R}}})/\mathop {\textrm{SO}}\nolimits _{1,1}({{\mathbb {R}}}) \cong \mathop {\textrm{dS}}\nolimits ^2\end{aligned}$$

is a realization of 2-dimensional de Sitter space. Note that the determinant defines a quadratic form of signature (1, 2) on $\mathop {\textrm{Sym}}\nolimits _2({{\mathbb {R}}})$ which is invariant under the action of the subgroup

$$\begin{aligned} \{ g \in \mathop {\textrm{GL}}\nolimits _2({{\mathbb {R}}}) : |\det (g)| = 1\} \supseteq \mathop {\textrm{SL}}\nolimits _2({{\mathbb {R}}})\end{aligned}$$

which acts as $\mathop {\textrm{SO}}\nolimits _{1,2}({{\mathbb {R}}})^{\uparrow }$.

For the Euler element $$h_s:= \frac{1}{2}\begin{pmatrix} 1 &{}\quad 0 \\ 0 &{}\quad -1 \end{pmatrix}$$, we have

$$\begin{aligned}{}[h_0, h_s] = z, \quad [z,h_s] = h_0 \quad \text{ and } \quad [h_0,z] = h_s.\end{aligned}$$

According to [53, Ex. 3.1(c)], all $\mathop {\textrm{Ad}}\nolimits (H)$-invariant cones in ${{\mathfrak {q}}}$ are Lorentzian of the form

$$\begin{aligned} C_m = \{ x_0 {{\textbf {1}}} + x_1 (h_s + z) + x_{-1} (h_s - z) : x_1 x_{-1} - m x_0^2 \ge 0, x_{\pm 1} \ge 0\} \quad \text{ for } \text{ some } \quad m > 0. \end{aligned}$$

Actually $C_0 = C_{{\mathfrak {q}}}^{\textrm{max}}$ contains ${{\mathbb {R}}}{{\textbf {1}}}$ and is not pointed.

(a) We write

$$\begin{aligned} h = \frac{\lambda + \mu }{2} {{\textbf {1}}} + h_s\end{aligned}$$

to see that $h \in C_m$ is equivalent to

$$\begin{aligned} m (\lambda +\mu )^2 \le 1.\end{aligned}$$

We also note that the “semisimple part” of $C_m$ is

$$\begin{aligned} C_{m,s} = C_m \cap {{\mathfrak {q}}}_s = C_m \cap ({{\mathbb {R}}}h + {{\mathbb {R}}}z) = \mathop {\textrm{cone}}\nolimits (h_s\pm z)\end{aligned}$$

coincides with the projection of $C_m$ to ${{\mathfrak {q}}}_s$, so that $C_{m,s}^\circ = C_m^\circ \cap {{\mathfrak {q}}}_s$.

Write $W(C_m,h)$ for the positivity domain of the Euler element *h* with respect to the causal structure specified by the cone $C_m$. Then Theorem 3.6 implies that

$$\begin{aligned} W(C_0, h_s) = G^{h_s}_e.\mathop {\textrm{Exp}}\nolimits _{eH}(\Omega _{{{\mathfrak {q}}}_{{\mathfrak {k}}}}), \quad \text{ where } \quad \Omega _{{{\mathfrak {q}}}_{{\mathfrak {k}}}} = \Big (-\frac{\pi }{2},\frac{\pi }{2}\Big ) z.\end{aligned}$$

For $x \in \Omega _{{{\mathfrak {q}}}_{{\mathfrak {k}}}}$ we have

$$\begin{aligned} e^{-\mathop {\textrm{ad}}\nolimits x}.h_s \in C_s + {{\mathfrak {h}}}\subseteq C_m + {{\mathfrak {h}}}\end{aligned}$$

(see (3.6)) and $G^h = G^{h_s}$, so that we have

$$\begin{aligned} W(C_0,h_s) = G^h_e.\mathop {\textrm{Exp}}\nolimits _{eH}(\Omega _{{{\mathfrak {q}}}_{{\mathfrak {k}}}}) \subseteq W(C_m,h_s) \subseteq W(C_0, h_s)\end{aligned}$$

implies the equality

$$\begin{aligned} W(C_m,h_s) = G^h_e.\mathop {\textrm{Exp}}\nolimits _{eH}(\Omega _{{{\mathfrak {q}}}_{{\mathfrak {k}}}}) \quad \text{ for } \text{ all } \quad m > 0.\end{aligned}$$

We also note that

$$\begin{aligned} W(C_m,h) \subseteq W(C_0,h) = W(C_0, h_s) = G^h_e.\mathop {\textrm{Exp}}\nolimits _{eH}(\Omega _{{{\mathfrak {q}}}_{{\mathfrak {k}}}}) \end{aligned}$$

because $\mathop {\textrm{Ad}}\nolimits (g)^{-1}h = h_z + \mathop {\textrm{Ad}}\nolimits (g)^{-1}h_s \in C_0^\circ$ if and only if $\mathop {\textrm{Ad}}\nolimits (g)^{-1}h_s \in C_s^\circ$.

To determine the domain $W(C_m,h)$ in general, we write

$$\begin{aligned} h = h_z + h_s = \frac{\lambda +\mu }{2}{{\textbf {1}}} + \frac{1}{2}\begin{pmatrix} 1 &{}\quad 0 \\ 0 &{} \quad -1 \end{pmatrix}.\end{aligned}$$

By $G^h_e$-invariance, we have to determine when $\mathop {\textrm{Exp}}\nolimits _{eH}(t z)$, $|t| < \frac{\pi }{2}$, is contained in $W(C_m,h)$. For $g = \exp (tz)$, we have

$$\begin{aligned} \mathop {\textrm{Ad}}\nolimits (g)^{-1} h = h_z + e^{-t \mathop {\textrm{ad}}\nolimits z} h_s = h_z + \cos (t) h_s - \sin (t) h_0, \end{aligned}$$

and

$$\begin{aligned} p_{{\mathfrak {q}}}(\mathop {\textrm{Ad}}\nolimits (g)^{-1}h) = h_z + \cos (t) h_s = \frac{\lambda +\mu }{2} {{\textbf {1}}} + \frac{\cos (t)}{2}(h_s + z) + \frac{\cos (t)}{2}(h_s - z).\end{aligned}$$

We then have

$$\begin{aligned} x_0 = \frac{\lambda + \mu }{2} \quad \text{ and } \quad x_{\pm 1} = \frac{\cos (t)}{2}.\end{aligned}$$

We conclude that, for $|t| < \frac{\pi }{2}$, the inclusion $h_z + \cos (t) h_s \in (C_m)^\circ$ is equivalent to

$$\begin{aligned} x_1 x_{-1} - m x_0^2 = \frac{1}{4}(\cos ^2(t) - m(\lambda + \mu )^2) > 0.\end{aligned}$$

We thus obtain the condition

$$\begin{aligned} |t| < \arccos (\sqrt{m} |\lambda + \mu |).\end{aligned}$$

For $m> 0$ and $h \not = h_s$, this is specifies a proper subinterval of $(-\frac{\pi }{2}, \frac{\pi }{2})$.

(b) To determine which cone $C_m$ corresponds to the canonical order on the space $\mathop {\textrm{Sym}}\nolimits _{1,1}({{\mathbb {R}}})$, induced from the natural order of $\mathop {\textrm{Sym}}\nolimits _2({{\mathbb {R}}})$ (which is also Lorentzian), we evaluate the tangent map ${{\mathfrak {q}}}\rightarrow \mathop {\textrm{Sym}}\nolimits _2({{\mathbb {R}}}), x \mapsto x I_{11} + I_{11} x^\top$ to

$$\begin{aligned} {{\textbf {1}}}.I_{1,1} = 2 I_{1,1}, \quad h_s.I_{1,1} = {{\textbf {1}}}, \quad z.I_{1,1} = \begin{pmatrix} 0 &{}\quad 1 \\ 1 &{}\quad 0 \end{pmatrix}.\end{aligned}$$

We thus obtain for $x = x_0 {{\textbf {1}}} + x_1 (h_s + z) + x_{-1} (h_s - z)$ that

$$\begin{aligned} x.I_{1,1} = x I_{11} + I_{11} x^\top = \begin{pmatrix} 2 x_0 + x_1 + x_{-1} &{}\quad x_1 - x_{-1} \\ x_1 - x_{-1} &{}\quad -2 x_0 + x_1 + x_{-1} \end{pmatrix}.\end{aligned}$$

By the Hurwitz criterion, this matrix is positive semidefinite if and only if

$$\begin{aligned} x_1 + x_{-1} \ge |2x_0| \end{aligned}$$

and

$$\begin{aligned} (x_1 + x_{-1})^2 - 4 x_0^2 - (x_1 - x_{-1})^2 = 4( x_1 x_{-1} - x_0^2) \ge 0.\end{aligned}$$

Is $x_1 + x_{-1} \ge 0$, then these two inequalities are equivalent to $x_1 x_{-1} - x_0^2 \ge 0$. As these two conditions imply that $x_{\pm 1} \ge 0$, we see that the canonical order on *M* corresponds to the cone $C^1$, i.e., to $m = 1$.

(c) For the modular vector field $X_h$, we have

$$\begin{aligned} X_h\begin{pmatrix} a &{}\quad b \\ b &{}\quad d \end{pmatrix} = \begin{pmatrix} 2\lambda a &{}\quad (\lambda + \mu ) b \\ (\lambda + \mu )b &{}\quad 2\mu d \end{pmatrix}.\end{aligned}$$

The positivity domain of $X_h$ depends on $\lambda$, and with this formula one can also determine the positivity domain quite directly for $m = 1$, where $C^1$ corresponds to the canonical order.

#### Example 3.10

(cf. [52, Exs. 2.11, 2.25]) Let $G:= \mathop {\textrm{GL}}\nolimits _n({{\mathbb {R}}})_+$ and $K:= \mathop {\textrm{SO}}\nolimits _n({{\mathbb {R}}})$. We consider the Riemannian symmetric space

$$\begin{aligned} M_r:= \mathop {\textrm{Sym}}\nolimits _n({{\mathbb {R}}})_+ \cong \mathop {\textrm{GL}}\nolimits _n({{\mathbb {R}}})/\mathop {\textrm{SO}}\nolimits _n({{\mathbb {R}}}) \end{aligned}$$

and the corresponding irreducible subspace

$$\begin{aligned} M_{r,s}:= \{ A \in M : \det (A) = 1\}\cong \mathop {\textrm{SL}}\nolimits _n({{\mathbb {R}}})/\mathop {\textrm{SO}}\nolimits _n({{\mathbb {R}}})\end{aligned}$$

(here the index *s* refers to “semisimple”). On ${{\mathfrak {g}}}= \mathop {{{\mathfrak {gl}} }}\nolimits _n({{\mathbb {R}}})$, we consider the Cartan involution given by $\theta (x) = - x^\top$ and write $n = p + q$ with $p,q> 0$. Then

3.12 $$\begin{aligned} h^p_s:= \frac{1}{n}\begin{pmatrix} q {{\textbf {1}}}_p &{}\quad 0 \\ 0 &{}\quad -p{{\textbf {1}}}_q \end{pmatrix} \in \mathop {{\mathfrak {sl} }}\nolimits _n({{\mathbb {R}}}) \quad \text{ and } \quad h^p:= h^p_s - \frac{q}{n}{{\textbf {1}}} = \begin{pmatrix} 0 &{}\quad 0 \\ 0 &{}\quad {{\textbf {1}}}_q \end{pmatrix} \in {{\mathfrak {g}}}\end{aligned}$$

are Euler elements and $\tau := \tau _{h^p}\theta$ leads to a non-compactly causal symmetric Lie algebra $({{\mathfrak {g}}},\tau ,C)$, where

$$\begin{aligned} {{\mathfrak {h}}}= \mathop {{\mathfrak {so} }}\nolimits _{p,q}({{\mathbb {R}}}) \quad \text{ and } \quad {{\mathfrak {q}}}= \Big \{\begin{pmatrix} a &{}\quad b \\ -b^\top &{}\quad d \end{pmatrix} : a^\top = a, d^\top = d \Big \}\end{aligned}$$

To identify *G*/*H* in the boundary of the crown domain in $G_{{\mathbb {C}}}/K_{{\mathbb {C}}}\cong G_{{\mathbb {C}}}.{{\textbf {1}}} \cong \mathop {\textrm{Sym}}\nolimits _n({{\mathbb {C}}})^\times$, where $G_{{\mathbb {C}}}$ acts on $\mathop {\textrm{Sym}}\nolimits _n({{\mathbb {C}}})$ by $g.A:= gAg^\top$ ([52, Thm. 5.4]), we observe that

$$\begin{aligned} \exp (it h^p).{{\textbf {1}}} = e^{2ith^p} = \cos (2t h^p) + i \sin (2t h^p) = \begin{pmatrix} {{\textbf {1}}}_p &{}\quad 0 \\ 0 &{}\quad (\cos (2t) + i \sin (2t)) {{\textbf {1}}}_q \end{pmatrix},\end{aligned}$$

so that we obtain for $t =\frac{\pi }{2}$ the matrix

$$\begin{aligned} \exp \big (\frac{\pi i}{2} h^p\big ).{{\textbf {1}}} = I_{p,q}.\end{aligned}$$

The *G*-orbit of this matrix is the open subset

$$\begin{aligned} M:= G.I_{p,q} = \{ gI_{p,q} g^\top : g \in \mathop {\textrm{GL}}\nolimits _n({{\mathbb {R}}})_+\} = \mathop {\textrm{Sym}}\nolimits _{p,q}({{\mathbb {R}}}) \end{aligned}$$

of symmetric matrices of signature (*p*, *q*). We have

$$\begin{aligned} X_{h^p}\begin{pmatrix} a &{}\quad b \\ b^\top &{}\quad d \end{pmatrix} = h^p \begin{pmatrix} a &{}\quad b \\ b^\top &{}\quad d \end{pmatrix} + \begin{pmatrix} a &{}\quad b \\ b^\top &{}\quad d \end{pmatrix}h^p = \begin{pmatrix} 0 &{}\quad b \\ b^\top &{}\quad 2 d \end{pmatrix}.\end{aligned}$$

These matrices are **never** positive definite. So we have to take $h_s$ instead to find non-trivial positivity domains.

For the case $p = q = 1$ and $n = 2$, this has been carried out in Example 3.9. We also write

$$\begin{aligned} h = \begin{pmatrix} \lambda {{\textbf {1}}}_p &{}\quad 0 \\ 0 &{}\quad \mu {{\textbf {1}}}_q \end{pmatrix} \quad \text{ with } \quad \lambda - \mu = 1.\end{aligned}$$

Then

$$\begin{aligned} X_h\begin{pmatrix} a &{}\quad b \\ b^\top &{}\quad d \end{pmatrix} = \begin{pmatrix} 2\lambda a &{}\quad (\lambda + \mu ) b \\ (\lambda + \mu ) b^\top &{}\quad 2\mu d \end{pmatrix},\end{aligned}$$

so that

$$\begin{aligned} X_h(I_{p,q}) = 2 \begin{pmatrix} \lambda {{\textbf {1}}}_p &{}\quad 0 \\ 0 &{}\quad -\mu {{\textbf {1}}}_q \end{pmatrix}>\!\!> 0 \quad \text{ if } \quad 0< \lambda < 1, \end{aligned}$$

which is equivalent to $\lambda \mu < 0$.

### The connected components of $M_C^h$

The main result in this section is Proposition 3.11 on the subgroup $H_K$ of $K^h$. We then discuss several examples to clarify the situation.

#### Proposition 3.11

(Connected components of $M^h_C$) If $G = \mathop {\textrm{Inn}}\nolimits ({{\mathfrak {g}}})$ and $({{\mathfrak {g}}},\tau )$ is irreducible ncc with causal Euler element *h*, then $\pi _0(M^h_C) \cong K^h/H_K$ contains at most two elements.

#### Proof

We recall from Proposition 3.2(c) that $M^h_C = G^h.eH$. With [36, Thm. IV.3.5] we see that the symmetric space $G^h.eH \cong G^h/H^h$ is a vector bundle over $K^h/H_K^h$, hence in particular homotopy equivalent to $K^h.eH \cong K^h/H_K^h$. In view of Proposition 2.6(c), we have for $G = \mathop {\textrm{Inn}}\nolimits ({{\mathfrak {g}}})$ that $H^h= H^{\tau _h} = H_K \subseteq G^\tau$ is a maximal compact subgroup of *H*. It follows in particular that $H^h = H_K^h \subseteq K^h$. We conclude that $\pi _0(M^h_C) \cong \pi _0(K^h/H_K)$. From [41, §7], we know that $\pi _0(G^h)\cong \pi _0(K^h)$ has at most two elements. $\square$

#### Example 3.12

(The inclusion $H_K \subseteq K^h$ may be proper) We have $G^h = K^h \exp ({{\mathfrak {q}}}_{{\mathfrak {p}}})$ and $K^{\tau ^G} = K^{\tau _h^G}$ because $K = G^\theta$. Further $H_K \subseteq K^h$ by Proposition 2.6(a), so that the equality $H_K = K^h$ is equivalent to $K^h \subseteq H_K$. This may fail for two reasons. One is failure in the adjoint group $\mathop {\textrm{Inn}}\nolimits ({{\mathfrak {g}}})$ (Proposition 3.11), and the other reason is that *Z*(*G*) may be non-trivial.

Assume that ${{\mathfrak {g}}}$ is semisimple and $({{\mathfrak {g}}},\tau , C)$ ncc. Let *G* be a corresponding connected Lie group on which $\tau ^G$ exists (for $\tau = \tau _h \theta$) and $H:= G^{\tau ^G}_e$. For the connected group $K:= G^\theta$, the intersection $H_K:= H \cap K = \langle \exp {{\mathfrak {h}}}_{{\mathfrak {k}}}\rangle$ is connected but $K^h \supseteq Z(G) H_K$ is in general not connected because *Z*(*G*) need not be contained in $H_K$.

This can be seen easily for ${{\mathfrak {g}}}= \mathop {{\mathfrak {sl} }}\nolimits _2({{\mathbb {R}}})$. For

3.13 $$\begin{aligned} h = \frac{1}{2}\begin{pmatrix} 1 &{} 0 \\ 0 &{} -1\end{pmatrix}, \quad \theta (x) = - x^\top , \quad \text{ we } \text{ have } \quad \tau = \theta \tau _h, \quad \begin{pmatrix} a &{} b\\ c &{} -a\end{pmatrix} \mapsto \begin{pmatrix} -a &{} c\\ b &{} a\end{pmatrix}.\nonumber \\ \end{aligned}$$

For any connected Lie group *G* with Lie algebra ${{\mathfrak {g}}}$, the group $K = G^\theta$ is connected 1-dimensional and $\tau (k) = k^{-1}$ for $k \in K$. Moreover, $K^h = Z(G)$ is a discrete subgroup which intersects $H = \exp {{\mathfrak {h}}}\cong {{\mathbb {R}}}$ trivially. Even the inclusion $K^h \subseteq G^{\tau ^G}$ fails if $|Z(G)| \ge 3$, i.e., if $\tau$ acts non-trivially on *Z*(*G*). Note that *Z*(*G*) is infinite if *G* is simply connected.

#### Example 3.13

(a) For ${{\mathfrak {g}}}= \mathop {{\mathfrak {sl} }}\nolimits _2({{\mathbb {R}}})$, we consider again the Euler element *h* from (3.13) and the Cartan involution $\theta (x) = -x^\top$. By Lemma B.1, the $\alpha$-orbit of *gH* is a geodesic if and only if $\mathop {\textrm{Ad}}\nolimits (g)^{-1}h$ commutes with $\tau (\mathop {\textrm{Ad}}\nolimits (g)^{-1}h) = - \mathop {\textrm{Ad}}\nolimits (\tau (g))^{-1}h$, i.e., if

$$\begin{aligned} \mathop {\textrm{Ad}}\nolimits (g\tau (g)^{-1})h \in {{\mathfrak {z}}}_{{\mathfrak {g}}}(h) = {{\mathbb {R}}}h.\end{aligned}$$

As ${\mathcal {O}}_h \cap {{\mathbb {R}}}h = \{ \pm h\}$, this leaves two possibilities:

1. If $\mathop {\textrm{Ad}}\nolimits (g\tau (g)^{-1})h= h$, then $\mathop {\textrm{Ad}}\nolimits (\tau (g))^{-1}h = \mathop {\textrm{Ad}}\nolimits (g)^{-1}h$ implies $\mathop {\textrm{Ad}}\nolimits (g)^{-1}h \in {{\mathfrak {q}}}$.
2. If $\mathop {\textrm{Ad}}\nolimits (g\tau (g)^{-1})h= -h$, then $-\mathop {\textrm{Ad}}\nolimits (\tau (g))^{-1}h = \mathop {\textrm{Ad}}\nolimits (g)^{-1}h$ implies $\mathop {\textrm{Ad}}\nolimits (g)^{-1}h \in {{\mathfrak {h}}}$. In this case *gH* is a fixed point of the modular flow.

(b) For ${{\mathfrak {g}}}= \mathop {{\mathfrak {sl} }}\nolimits _{2k}({{\mathbb {R}}})$ with the Cartan involution $\theta (x) = - x^\top$ and the causal Euler element

$$\begin{aligned} h = \frac{1}{2} \begin{pmatrix} {{\textbf {1}}}_k &{}\quad 0 \\ 0 &{}\quad -{{\textbf {1}}}_k \end{pmatrix},\end{aligned}$$

we obtain ${{\mathfrak {h}}}= \mathop {{\mathfrak {so} }}\nolimits _{k,k}({{\mathbb {R}}})$ for $\tau = \theta \tau _h$. There exists a subalgebra ${{\mathfrak {s}}}\cong \mathop {{\mathfrak {sl} }}\nolimits _2({{\mathbb {R}}})^k$, where the $\mathop {{\mathfrak {sl} }}\nolimits _2$-factors correspond to the coordinates $x_j$ and $x_{j + k}$ for $1 \le j \le k$. Accordingly, $h = \sum _{j = 1}^k h_j$, where the Euler elements $h_j$ in the $\mathop {{\mathfrak {sl} }}\nolimits _2$-factors are conjugate to Euler elements $h_j'$ in ${{\mathfrak {h}}}$. Therefore, the “geodesic condition” is satisfied by all elements $\sum _{j = 1}^k {\widetilde{h}}_j \in {\mathcal {O}}_h$, where ${\widetilde{h}}_j$ is either $h_j$ or $h_j'$.

The following example shows that modular geodesics also exist in symmetric spaces without causal structure. They can be “space-like” rather than “time-like”, resp., causal.

#### Example 3.14

The *d*-dimensional hyperbolic space

$$\begin{aligned} {{\mathbb {H}}}^d:= \{ x = (x_0, {{\textbf {x}}}) \in {{\mathbb {R}}}^{1,d} : x_0^2 - {{\textbf {x}}}^2 = 1, x_0 > 0 \} \cong \mathop {\textrm{SO}}\nolimits _{1,d}^{\uparrow }({{\mathbb {R}}})/\mathop {\textrm{SO}}\nolimits _d({{\mathbb {R}}}) \end{aligned}$$

carries a modular flow specified by any Euler element $h \in {{\mathfrak {q}}}\subseteq \mathop {{\mathfrak {so} }}\nolimits _{1,d}({{\mathbb {R}}})$ (corresponding to a tangent vector of length 1). Every geodesic of ${{\mathbb {H}}}^d$ is an orbit of the flow generated by an Euler element of $\mathop {{\mathfrak {so} }}\nolimits _{1,d}({{\mathbb {R}}})$.

#### Remark 3.15

Let $({{\mathfrak {g}}},\tau ,C)$ be a simple ncc symmetric Lie algebra. In general, we have for a causal Euler element $h \in {\mathcal {E}}({{\mathfrak {g}}}) \cap C^\circ$ a proper inclusion

$$\begin{aligned} \mathop {\textrm{Inn}}\nolimits _{{\mathfrak {g}}}({{\mathfrak {h}}})h = {\mathcal {O}}_h \cap C^\circ \subseteq {\mathcal {O}}_h \cap {{\mathfrak {q}}}.\end{aligned}$$

By Lemma B.4, this implies that $M^h$ is not connected and $M^h_C \not = M^h$.

For instance, if ${{\mathfrak {g}}}= {{\mathfrak {h}}}_{{\mathbb {C}}}$ and ${{\mathfrak {h}}}$ is simple hermitian of tube type, then we obtain for any pointed generating invariant cone $C_{{\mathfrak {h}}}\subseteq {{\mathfrak {h}}}$ a hyperbolic cone $C:= i C_{{\mathfrak {h}}}\subseteq {{\mathfrak {q}}}= i {{\mathfrak {h}}}$. If $h \in {\mathcal {E}}({{\mathfrak {g}}}) \cap C^\circ$ is a causal Euler element, then $-h \in \mathop {\textrm{Ad}}\nolimits (G)h$ follows from [38, Thm. 3.10] and the subsequent discussion, but $-h \not \in C^\circ$; see also [41, Thm. 5.4].

#### Example 3.16

(a) For de Sitter space $M = \mathop {\textrm{dS}}\nolimits ^d$ (cf. Example 4.6 and Appendix D), the subspace $M^h_{eH} = \mathop {\textrm{Exp}}\nolimits _{eH}({{\mathbb {R}}}h)$ is a single geodesic, hence in particular 1-dimensional. Note that $\mathop {\textrm{dim}}\nolimits {{\mathfrak {q}}}_{{\mathfrak {p}}}= 1$ in this case. The modular flow on *M* has the fixed point set $M^\alpha \cong {{\mathbb {S}}}^{d-2}$.

(b) For $M = G_{{\mathbb {C}}}/G$, ${{\mathfrak {g}}}$ hermitian, we have $M^h_{eG} = \mathop {\textrm{Exp}}\nolimits _e(i{{\mathfrak {k}}})$ with dual symmetric space the group *K*, considered as a symmetric space.

## Open *H*-orbits in flag manifolds and a convexity theorem

In this section, we prove a convexity theorem that is vital to derive the equality $W = W(\gamma )$ in the next section. Here, as above, $W= W_M^+(h)_{eH}$.

Let $P^-:= \exp ({{\mathfrak {g}}}_{-1}(h)) G^h \subseteq G$ be the “negative” parabolic subgroup of *G* specified by *h* and identity ${{\mathfrak {g}}}_1(h)$ with the open subset ${\mathcal {B}}:= \exp ({{\mathfrak {g}}}_1(h)).eP^- \subseteq G/P^-$. Then ${\mathcal {D}}:= H.0 \subseteq {\mathcal {B}}$ is an open convex subset, and our convexity theorem (Theorem 4.5) asserts that, for any $g \in G$ with $g.{\mathcal {D}}\subseteq {\mathcal {B}}$, the subset $g.{\mathcal {D}}\subseteq {\mathcal {B}}$ is convex.

We consider a **connected semisimple** Lie group *G* with Lie algebra ${{\mathfrak {g}}}$ and an Euler element $h \in {{\mathfrak {g}}}$. We put

$$\begin{aligned} {{\mathfrak {n}}}^\pm := {{\mathfrak {g}}}_{\pm 1}(h) \quad \text{ and } \quad N^\pm := \exp ({{\mathfrak {n}}}^\pm ), \end{aligned}$$

and write

$$\begin{aligned} P^\pm := \{ g \in G : \mathop {\textrm{Ad}}\nolimits (g) {{\mathfrak {g}}}_{\pm 1}(h) = {{\mathfrak {g}}}_{\pm 1}(h)\} = N^\pm G^h \cong N^\pm \rtimes G^h\end{aligned}$$

(see [2, Thm. 1.12] for the equality) for the corresponding maximal parabolic subgroups. We write

$$\begin{aligned} {\mathcal {M}}_\pm := G/P^{\mp } \end{aligned}$$

for the corresponding flag manifold. The abelian subgroup $N^+$ has an open orbit ${\mathcal {B}}:= N^+.eP^- \subseteq {\mathcal {M}}_+$, which we call the *open Bruhat cell*. It carries a natural affine structure because the map

$$\begin{aligned} \varphi : {{\mathfrak {n}}}^+ \rightarrow {\mathcal {B}}:= N^+.eP^-, \quad x \mapsto \exp (x) P^- \end{aligned}$$

defines an open embedding. Below we shall always use these coordinates on ${\mathcal {B}}$.

Choose a Cartan involution $\theta$ with $\theta (h) = -h$ and consider the involution $\tau := \theta e^{\pi i \mathop {\textrm{ad}}\nolimits h}$. We write

$$\begin{aligned} H:= K^h \exp ({{\mathfrak {h}}}_{{\mathfrak {p}}}) \quad \text{ for } \quad {{\mathfrak {h}}}_{{\mathfrak {p}}}= {{\mathfrak {g}}}^\tau \cap {{\mathfrak {p}}}\quad \text{ and } \quad H_K:= K^h.\end{aligned}$$

Then

$$\begin{aligned} P^{\pm }\cap H = G^h \cap H = K^h, \end{aligned}$$

so that

4.1 $$\begin{aligned} {\mathcal {D}}_+:= H.eP^- \cong H/H_K \end{aligned}$$

is an open *H*-orbit in ${\mathcal {B}}\subseteq G/P^-$. It is a real bounded symmetric domain ([26, Thm. 5.1.8]) and coincides with the unit ball in the positive real Jordan triple

4.2 $$\begin{aligned} V:= {{\mathfrak {n}}}\quad \text{ with } \quad \{x,y,z\}:= x \square y.z = - \frac{1}{2}[[x,\theta (y)],z] \end{aligned}$$

(cf. [2, (4.6)])

### The open *H*-orbits in $G/P^\pm$

#### Lemma 4.1

([2, Cor. 1.10]) For $y \in {{\mathfrak {g}}}_{-1}(h)$ and $x \in {{\mathfrak {g}}}_1(h)$, we have $\exp (y).\exp (x) P^- \in {\mathcal {B}}$ if and only if the *Bergman operators*

$$\begin{aligned} B_+(x,y):= {{\textbf {1}}} + \mathop {\textrm{ad}}\nolimits (x)\mathop {\textrm{ad}}\nolimits (y) + \frac{1}{4} (\mathop {\textrm{ad}}\nolimits x)^2 (\mathop {\textrm{ad}}\nolimits y)^2 \in \mathop {\textrm{End}}\nolimits ({{\mathfrak {g}}}_1(h)) \end{aligned}$$

and

$$\begin{aligned} B_-(y,x):= {{\textbf {1}}} + \mathop {\textrm{ad}}\nolimits (y)\mathop {\textrm{ad}}\nolimits (x) + \frac{1}{4} (\mathop {\textrm{ad}}\nolimits y)^2 (\mathop {\textrm{ad}}\nolimits x)^2 \in \mathop {\textrm{End}}\nolimits ({{\mathfrak {g}}}_{-1}(h))\end{aligned}$$

are both invertible.

#### Remark 4.2

Note that

$$\begin{aligned} \theta B_-(y,x) \theta = {{\textbf {1}}} + \mathop {\textrm{ad}}\nolimits (\theta (y))\mathop {\textrm{ad}}\nolimits (\theta (x)) + \frac{1}{4} (\mathop {\textrm{ad}}\nolimits \theta (y))^2 (\mathop {\textrm{ad}}\nolimits \theta (x))^2 = B_+(\theta (y),\theta (x)).\end{aligned}$$

#### Example 4.3

We consider the group $G = \mathop {\textrm{SL}}\nolimits _2({{\mathbb {R}}})$ with Lie algebra ${{\mathfrak {g}}}= \mathop {{\mathfrak {sl} }}\nolimits _2({{\mathbb {R}}})$ and the linear basis

4.3 $$\begin{aligned} h:= \frac{1}{2} \begin{pmatrix} 1 &{}\quad 0 \\ 0 &{}\quad -1 \end{pmatrix},\quad e = \begin{pmatrix} 0 &{}\quad 1 \\ 0 &{}\quad 0 \end{pmatrix}, \quad f = \begin{pmatrix} 0 &{}\quad 0 \\ 1 &{}\quad 0 \end{pmatrix}, \end{aligned}$$

satisfying

$$\begin{aligned}{}[h,e] = e, \quad [h,f] = -f, \quad [e,f] = 2h.\end{aligned}$$

Then,

$$\begin{aligned} N^+ = \begin{pmatrix} 1 &{}\quad {{\mathbb {R}}}\\ 0 &{}\quad 1 \end{pmatrix}, \quad N^- = \begin{pmatrix} 1 &{}\quad 0 \\ {{\mathbb {R}}}&{}\quad 1 \end{pmatrix}, \quad \text{ and } \quad G^h = \Big \{ \begin{pmatrix} a &{}\quad 0 \\ 0 &{}\quad a^{-1} \end{pmatrix} : a \in {{\mathbb {R}}}^\times \Big \},\end{aligned}$$

so that

$$\begin{aligned} P^- = \Big \{ \begin{pmatrix} a &{}\quad 0 \\ c &{}\quad a^{-1} \end{pmatrix} : a \in {{\mathbb {R}}}^\times , c \in {{\mathbb {R}}}\Big \} = \{ g \in G : g e_2 \in {{\mathbb {R}}}e_2\}.\end{aligned}$$

For $K = \mathop {\textrm{SO}}\nolimits _2({{\mathbb {R}}})$, we have $K^h = \{ \pm {{\textbf {1}}}\}$. Identifying $G/P^-$ with the projective space ${{\mathbb {P}}}({{\mathbb {R}}}^2) = G.[e_2]$, the Bruhat cell is

$$\begin{aligned} {\mathcal {B}}= \{ [x:1] : x \in {{\mathbb {R}}}\} \cong {{\mathbb {R}}},\end{aligned}$$

and *G* acts by

$$\begin{aligned} g.x = \frac{ax + b}{cx+d}\quad \text{ for } \quad ax + b \not =0.\end{aligned}$$

In particular, we have

4.4 $$\begin{aligned} \exp (yf).x = \frac{x}{1 + xy}. \end{aligned}$$

We consider the Cartan involution $\theta (x) = -x^\top$, so that $\tau := \theta e^{\pi i \mathop {\textrm{ad}}\nolimits h}$ acts by

$$\begin{aligned} \tau \begin{pmatrix} a &{}\quad b \\ c &{}\quad -a \end{pmatrix} = \begin{pmatrix} -a &{}\quad c \\ b &{}\quad a \end{pmatrix} \quad \text{ and } \quad \tau ^G\begin{pmatrix} a &{}\quad b \\ c &{}\quad d \end{pmatrix} = \begin{pmatrix} d &{}\quad c \\ b &{}\quad a \end{pmatrix}. \end{aligned}$$

Then

$$\begin{aligned} H = \mathop {\textrm{SL}}\nolimits _2({{\mathbb {R}}})^{\tau ^G} = \Big \{ \begin{pmatrix} a &{}\quad b \\ b &{}\quad a \end{pmatrix} : a^2 - b^2 = 1\Big \} = \mathop {\textrm{SO}}\nolimits _{1,1}({{\mathbb {R}}}),\end{aligned}$$

so that

4.5 $$\begin{aligned} {\mathcal {D}}_+ = H.0 = \{ ba^{-1} : a^2 - b^2 = 1\} = (-1,1). \end{aligned}$$

Note that $\mathop {\textrm{Ad}}\nolimits (H) \cong H/\{ \pm {{\textbf {1}}}\}$ is connected.

The Jordan triple product satisfies

$$\begin{aligned} \{e,e,e\} = - \frac{1}{2}[[e,\theta (e)],e] = -\frac{1}{2}[[e,-f],e] = \frac{1}{2}[2h,e] = e,\end{aligned}$$

so that

$$\begin{aligned} \{xe,ye,ze\} = xyz \cdot e\quad \text{ for } \quad x,y,z \in {{\mathbb {R}}}.\end{aligned}$$

Further

$$\begin{aligned} (\mathop {\textrm{ad}}\nolimits f)e = -2h, \quad (\mathop {\textrm{ad}}\nolimits f)^2e = -2[f,h] = -2f, \quad (\mathop {\textrm{ad}}\nolimits e)f = 2h, \quad (\mathop {\textrm{ad}}\nolimits e)^2f = -2e\end{aligned}$$

implies

$$\begin{aligned} B_+(x e, y f) e&= e + xy \mathop {\textrm{ad}}\nolimits (e) \mathop {\textrm{ad}}\nolimits (f)e + \frac{x^2y^2}{4} (\mathop {\textrm{ad}}\nolimits e)^2 (\mathop {\textrm{ad}}\nolimits f)^2 e\\&= e + xy \mathop {\textrm{ad}}\nolimits (e)(-h) + \frac{x^2y^2}{4} (\mathop {\textrm{ad}}\nolimits e)^2 (-2f) \\&= e + xy 2 e + \frac{x^2y^2}{4} 4 e = (1 + 2 xy + x^2 y^2) e = (1 + xy)^2 e. \end{aligned}$$

Moreover,

$$\begin{aligned} B_+(\theta (yf), \theta (xe))e = B_+(-ye, -xf)e = (1 + (-y)(-x))^2 e. = B_+(xe,yf)e. \end{aligned}$$

As $1 +xy$ is invertible for all *x* with $|x| < 1$ if and only if $|y| \le 1$, it follows that

$$\begin{aligned} \exp (y).{\mathcal {D}}_+ \subseteq {\mathcal {B}}\quad \Leftrightarrow \quad |y| \le 1.\end{aligned}$$

Now back to the general case. In the following we write $\Vert \cdot \Vert$ for the spectral norm on the Jordan triple system ${{\mathfrak {g}}}_1(h) = {{\mathfrak {n}}}^+$. If $x = \sum _{j = 1}^k x_j c_j$ with pairwise orthogonal tripotents $c_j$, then

4.6 $$\begin{aligned} \Vert x\Vert = \max \{ |x_1|, \ldots , |x_k|\}. \end{aligned}$$

If

4.7 $$\begin{aligned} {\mathcal {D}}_{{\mathfrak {g}}}:= \{ x \in {{\mathfrak {g}}}_1(h) : \Vert x\Vert < 1 \}, \end{aligned}$$

then we have

4.8 $$\begin{aligned} {\mathcal {D}}_+ = \exp ({\mathcal {D}}_{{\mathfrak {g}}}) P^- \subseteq G/P^- \end{aligned}$$

([26, Thm. 5.1.8]).

#### Proposition 4.4

The following assertions hold:

- $g.{\mathcal {D}}_+ \subseteq {\mathcal {B}}$ is equivalent to $g \in P^+ \exp (y)$ for $y \in {{\mathfrak {n}}}^-$ with $\Vert y\Vert \le 1$.
- $g.{\mathcal {D}}_+ \subseteq {\mathcal {B}}$ is relatively compact if and only if $g \in P^+ \exp (y)$ for $y \in {{\mathfrak {n}}}^-$ with $\Vert y\Vert <1$.

#### Proof

The condition $g.eP^- \in {\mathcal {B}}$ is equivalent to $g \in N^+ P^- = N^+ G^h N^- = P^+ N^-.$ Let $y \in {{\mathfrak {n}}}^-$ with $g \in P^+ \exp (y)$. Then the invariance of ${\mathcal {B}}$ under $P^+$ implies that $g.{\mathcal {D}}_+ \subseteq {\mathcal {B}}$ is equivalent to $\exp (y).{\mathcal {D}}_+ \subseteq {\mathcal {B}}$.

(a) Suppose first that $\exp (y).{\mathcal {D}}_+ \subseteq {\mathcal {B}}$. By the Spectral Theorem for positive Jordan triples ([59, Thm. VI.2.3]^3^), there exist pairwise orthogonal tripotents $c_1, \ldots , c_k$ and $\beta _1, \ldots , \beta _k \in {{\mathbb {R}}}$ with

$$\begin{aligned} y = \sum _{j = 1}^k \beta _j \theta (c_j)\end{aligned}$$

([59, Thm. VI.2.3]). For $x = \sum _j \alpha _j c_j$ and $z = \sum _j \gamma _j c_j$, we then have

$$\begin{aligned} \{x,\theta (y),z\} = \sum _{j = 1}^k \alpha _j \beta _j \gamma _j \cdot c_j \end{aligned}$$

([59, Prop. V.3.1]). As $x \in {\mathcal {D}}_{{\mathfrak {g}}}$ is equivalent to

$$\begin{aligned} \Vert x\Vert = \max \{ |\alpha _j| : j =1,\ldots , k\} < 1,\end{aligned}$$

the calculations in Example 4.3 show that $\exp (y).{\mathcal {D}}_+ \subseteq {\mathcal {B}}$ implies $\Vert y\Vert \le 1$.^4^

To prove the converse, suppose first that $\Vert y\Vert < 1$. Then

$$\begin{aligned} \exp (-y) = \theta (\exp (-\theta (y)) \in \theta (H P^-) = H P^+ \end{aligned}$$

implies $\exp (y) \in P^+ H,$ so that

$$\begin{aligned} \exp (y).{\mathcal {D}}_+ \in P^+H.{\mathcal {D}}_+ = P^+.{\mathcal {D}}_+ \subseteq {\mathcal {B}}.\end{aligned}$$

Now we assume that $\Vert y\Vert = 1$. We observe that

$$\begin{aligned} \exp (y).x = \exp (-th) \exp (th) \exp (y).x = \exp (-th) \exp (e^{-t} y).(e^t x),\end{aligned}$$

so that, for $r > 0$, $\exp (y).x \in {\mathcal {B}}$ is equivalent to $\exp (r^{-1}y).(rx) \in {\mathcal {B}}$. For $x \in {\mathcal {D}}_{{\mathfrak {g}}}$, we pick $r > 1$ with $rx \in {\mathcal {D}}_{{\mathfrak {g}}}$. Then $\Vert r^{-1}y\Vert < 1$ implies $\exp (r^{-1}.y). \exp (rx) \in {\mathcal {B}},$ and thus $\exp (y).\exp (x) \in {\mathcal {B}}$. This shows that $\exp (y).{\mathcal {D}}_+ \subseteq {\mathcal {B}}$.

(b) If $\Vert y\Vert < 1$, then the argument under (a) shows that $\exp (y).{\mathcal {D}}_+ \subseteq P^+.{\mathcal {D}}_+$ is relatively compact.

Now we assume that $\Vert y\Vert = 1$. We show that this implies that $\exp (y).{\mathcal {D}}_+$ is unbounded. As above, we use the Spectral Theorem to write

$$\begin{aligned} y = \sum _{j = 1}^k \beta _j \theta (c_j)\end{aligned}$$

and observe that there exists an $\ell \in \{1,\ldots , k\}$ with $|b_{\ell }| = 1$. For $x = \sum _j \alpha _j c_j \in {\mathcal {D}}_{{\mathfrak {g}}}$, we then obtain with (4.4)

$$\begin{aligned} \exp (y).x = \sum _{j = 1}^k \frac{\alpha _j}{1 + \alpha _j \beta _j} c_j.\end{aligned}$$

For $x = \alpha c_{\ell }$ we get in particular

$$\begin{aligned} \exp (y).x = \frac{\alpha }{1 + \alpha \beta _{\ell }} c_{\ell }.\end{aligned}$$

For $\alpha \rightarrow -\mathop {\textrm{sgn}}\nolimits (\beta _\ell )$ these element leave every compact subset of ${\mathcal {B}}$. Therefore, $\exp (y).{\mathcal {D}}_+$ is unbounded. $\square$

#### Theorem 4.5

(Convexity theorem for conformal balls) If $g \in G$ is such that $g.{\mathcal {D}}_+ \subseteq {\mathcal {B}}$, then $g{\mathcal {D}}_+$ is convex. If $g.{\mathcal {D}}_+$ is relatively compact in ${\mathcal {B}}$, then there exists an element $p \in P^+$ with $g.{\mathcal {D}}_+ = p.{\mathcal {D}}_+$, so that $g.{\mathcal {D}}_+$ is an affine image of ${\mathcal {D}}_+$.

#### Proof

If $g.{\mathcal {D}}_+ \subseteq {\mathcal {B}}$ is relatively compact, then Proposition 4.4(b) and its proof imply the existence of $p \in P^+$ with $g.{\mathcal {D}}_+ = p.{\mathcal {D}}_+$. In particular $g.{\mathcal {D}}_+$ is an affine image of ${\mathcal {D}}_+$ and therefore convex.

If $g.{\mathcal {D}}_+\subseteq {\mathcal {B}}$ is not relatively compact, then we put $r_n:= 1-\frac{1}{n}$. Now

$$\begin{aligned} \exp y.{\mathcal {D}}_+ = \bigcup _{n \in {{\mathbb {N}}}} \exp y\exp (r_n{\mathcal {D}}_{{\mathfrak {g}}})P^- \end{aligned}$$

is an increasing union. Therefore it suffices to show that the subsets $\exp y\exp (r_n{\mathcal {D}}_{{\mathfrak {g}}})P^-$ are convex. For $r_n = e^t$ we have

$$\begin{aligned} e^{-t}\cdot (\exp y\exp (r_n {\mathcal {D}}_{{\mathfrak {g}}})P^-)&= \exp (-th).(\exp (y)\exp (r_n {\mathcal {D}}_{{\mathfrak {g}}})P^-) \\&= \exp (e^t y).\exp (e^{-t}r_n {\mathcal {D}}_{{\mathfrak {g}}})P^- = \exp (r_n y).{\mathcal {D}}_+, \end{aligned}$$

and these sets are convex by the preceding argument. $\square$

#### Example 4.6

We consider $G = \mathop {\textrm{SO}}\nolimits _{1,d}({{\mathbb {R}}})_e$ as the identity component of the conformal group of the Euclidean space ${{\mathbb {R}}}^{d-1}$, $H = G_{{{\textbf {e}}}_1} = \mathop {\textrm{SO}}\nolimits _{1,d-1}({{\mathbb {R}}})$, and the Euler element $h \in \mathop {{\mathfrak {so} }}\nolimits _{1,1}({{\mathbb {R}}})$ with $h.{{\textbf {e}}}_0 = {{\textbf {e}}}_1$ and $h.{{\textbf {e}}}_1 = {{\textbf {e}}}_0$. As $Z(\mathop {\textrm{SO}}\nolimits _{1,d-1}({{\mathbb {R}}})) \subseteq \{ \pm {{\textbf {1}}}\}$ and *G* preserves the positive light cone, the center of *G* is trivial.

The symmetric space $M = G/H \cong G.{{\textbf {e}}}_1 \cong \mathop {\textrm{dS}}\nolimits ^d$ is *d*-dimensional de Sitter space, $P = N^- \rtimes G^h$ is the stabilizer of the positive light ray ${{\mathbb {R}}}_+({{\textbf {e}}}_0 - {{\textbf {e}}}_1)$, and $G/P \cong {{\mathbb {S}}}^{d-1}$ is the sphere of positive light rays. On the sphere ${{\mathbb {S}}}^{d-1}$, the subgroup *H* has two open orbits which are positive half-spheres separated by the sphere ${{\mathbb {S}}}^{d-2}$ of positive light rays in the subspace ${{\textbf {e}}}_1^\bot$.

In the sphere the Bruhat cells are the point complements and if $g.{\mathcal {D}}_+ \subseteq {\mathcal {B}}\cong {{\mathbb {R}}}^{d-1}$, then the convexity of $g.{\mathcal {D}}_+$ is well-known from conformal geometry because conformal images of balls are balls or half spaces.

### The subset realization of the ordered space $M = G/H$

As before *G* is assumed to be a connected semisimple Lie group. To simplify the notation, we write ${\mathcal {M}}$ for ${\mathcal {M}}_+ = G/P^-$. Recall the following fact about the compression semigroup of the *H*-orbit ${\mathcal {D}}_+ = H.eP^- \subseteq {\mathcal {M}}_+$, which is the Riemannian symmetric space $H/H\cap K$.

#### Lemma 4.7

The compression semigroup of the open *H*-orbit ${\mathcal {D}}_+ = H.eP^-\subseteq G/P^-$ is

4.9 $$\begin{aligned} \mathop {\textrm{comp}}\nolimits ({{\mathcal {D}}_+}) = \{ g \in G : g.{{\mathcal {D}}_+} \subseteq {{\mathcal {D}}_+} \} = H \exp (-C_{{\mathfrak {q}}}^{\textrm{max}}(h)), \end{aligned}$$

#### Proof

This result was announced in [57, 58], and a detailed proof was given in [24, Thm. VI.11] for the case where $G \subseteq G_{{\mathbb {C}}}$, $G_{{\mathbb {C}}}$ is simply connected and $H = G^\tau$. In this case $\mathop {\textrm{Ad}}\nolimits (G^\tau )$ preserves $C_{{\mathfrak {q}}}^{\textrm{max}}(h)$, so that $G^\tau \subseteq K^h \exp ({{\mathfrak {h}}}_{{\mathfrak {p}}})$. Conversely, $K^h$ leaves ${\mathcal {D}}_+$ invariant, so that we obtain $G^\tau = H = K^h \exp ({{\mathfrak {h}}}_{{\mathfrak {p}}})$ in this particular case.

To see that the lemma also holds in the general case, note that the center of *G* acts trivially on $G/P^-$ and that $Z(G) \subseteq K^h\subseteq H$. Therefore, the general assertion follows if the equality (4.9) holds at least for one connected Lie group *G* with Lie algebra ${{\mathfrak {g}}}$. Hence, it follows from the special case discussed above. $\square$

We now use this to realize *G*/*H* as an ordered symmetric space as a set of subsets of ${\mathcal {M}}$ and describe the ordering in that realization.

#### Proposition 4.8

(The subset realization of ncc symmetric spaces) Let *G* be a connected semisimple Lie group, $h \in {{\mathfrak {g}}}$ an Euler element, $\theta$ a Cartan involution with $\theta (h) = -h$ and $\tau :=\theta \tau _h$, so that $({{\mathfrak {g}}},\tau )$ is a ncc symmetric Lie algebras with ${{\mathfrak {g}}}= {{\mathfrak {g}}}_s$. Let ${\mathcal {D}}_+\subseteq G/P^-$ be the open orbit of the base point under $H:= K^h \exp ({{\mathfrak {h}}}_{{\mathfrak {p}}})$. We endow the homogeneous space

$$\begin{aligned} M_{{\mathcal {D}}_+}:= \{g.{\mathcal {D}}_+ : g \in G \}, \end{aligned}$$

consisting of subsets of ${\mathcal {M}}$, with the inclusion order. Then the stabilizer subgroup $G^{{\mathcal {D}}_+}$ of the base point is *H*. The map $gH \mapsto g.{\mathcal {D}}_+$ induces an isomorphism

$$\begin{aligned} (M_{{\mathcal {D}}_+}, \subseteq ) \cong (G/H, C_{{\mathfrak {q}}}^{\textrm{max}}(h)) \end{aligned}$$

of ncc symmetric spaces, where $C_{{\mathfrak {q}}}^{\textrm{max}}(h)$ is the unique maximal $\mathop {\textrm{Ad}}\nolimits (H)$-invariant cone in ${{\mathfrak {q}}}$ containing *h* in its interior.

In this identification, the set $\{x\in G/H: x\ge eH\}$ is mapped to $\{s^{-1} {\mathcal {D}}_+ : s\in \mathop {\textrm{comp}}\nolimits ({{\mathcal {D}}_+}) \}$ and $\{x\in G/H : x\le eH\}$ is mapped to $\{s {\mathcal {D}}_+ : s\in \mathop {\textrm{comp}}\nolimits ({{\mathcal {D}}_+}) \}$. In particular, $gH\ge eH$ is equivalent to ${\mathcal {D}}_+\subset g.{\mathcal {D}}_+$ and $eH \ge gH$ to $g.{\mathcal {D}}_+\subset {\mathcal {D}}_+$.

#### Proof

This follows from Lemma 4.7. $\square$

#### Remark 4.9

(The Riemannian case) Let $({{\mathfrak {g}}},\theta )$ be a Riemannian symmetric Lie algebra, i.e., ${{\mathfrak {g}}}= {{\mathfrak {g}}}_r$. Then $H = K$, $M = G/K$ and $h=0$. Thus $G= P^-$ and $G/P^-$ is a single point. Hence $\mathop {\textrm{comp}}\nolimits ({\mathcal {D}}_+) = G$ and ${\mathcal {M}}_{{\mathcal {D}}_+}$ is a single point. Therefore, Riemannian summands cannot be permitted in Proposition 4.8.

#### Example 4.10

Let $G=\mathop {\textrm{SL}}\nolimits _2({{\mathbb {R}}})$ and $$h = \frac{1}{2}\begin{pmatrix} 1 &{}\quad 0 \\ 0 &{}\quad -1 \end{pmatrix}$$. Then, the canonical action of *G* on ${{\mathbb {P}}}_1({{\mathbb {R}}}) = {{\mathbb {P}}}({{\mathbb {R}}}^2) \cong {{\mathbb {S}}}^1 = {{\mathbb {R}}}\cup \{\infty \}$ is given by

$$\begin{aligned}\begin{pmatrix} a &{} b\\ c &{} d\end{pmatrix}.x = \frac{a x + b}{c x + d}\end{aligned}$$

and the stabilizer of 0 is

$$\begin{aligned}P^- = \Big \{ \begin{pmatrix} a &{}\quad 0 \\ c &{}\quad a^{-1} \end{pmatrix} : a \not =0, c \in {{\mathbb {R}}}\Big \} = \exp ({{\mathfrak {g}}}_{-1}(h)) \rtimes G^h, \quad G^h = \Big \{\begin{pmatrix} a &{}\quad 0 \\ 0 &{}\quad a^{-1} \end{pmatrix} \Big \} \cong {{\mathbb {R}}}^\times . \end{aligned}$$

The 1-parameter group

$$\begin{aligned} a_t = \begin{pmatrix} \cosh t &{} \sinh t \\ \sinh t &{} \cosh t \end{pmatrix} \end{aligned}$$

fixes $\pm 1$ and the orbit ${\mathcal {D}}_+ = H.0$ of $H = \{\pm a_t : t \in {{\mathbb {R}}}\}$ is the open unit interval ${\mathcal {D}}_+ = (-1,1)$. The maximal cone in ${{\mathfrak {q}}}$ is generated by $\mathop {\textrm{Ad}}\nolimits (H){{\mathbb {R}}}_+ h$.

Since elements of ${{\mathbb {P}}}_1({{\mathbb {R}}})$ represent one-dimensional linear subspaces of ${{\mathbb {R}}}^2$ and $\mathop {\textrm{SL}}\nolimits _2({{\mathbb {R}}})$ acts transitively on triples of such subspaces, it follows easily that it acts transitively on the set of non-dense open intervals $I \subseteq {{\mathbb {S}}}^1$, the ordered space *G*/*H* can be identified with the ordered set of open non-dense intervals in ${{\mathbb {S}}}^1$.

#### Example 4.11

A special case of the above construction is the “complex case” where *H* is a connected semisimple Lie group of hermitian type contained in a complex Lie group *G* with Lie algebra ${{\mathfrak {h}}}_{{\mathbb {C}}}= {{\mathfrak {h}}}\oplus i{{\mathfrak {h}}}$. Then, *G*/*H* is a ncc symmetric space. Let $\theta _H$ be a Cartan involution on *H*. Then $\theta _H$ extends to a Cartan involution $\theta$ on *G*. Denote the corresponding maximal compact subgroup of *G* by *K*. Then $H\cap K$ is a maximal compact subgroup of *H* and the Riemannian symmetric space $H/H\cap K$ can be realized as complex symmetric bounded domain ${\mathcal {D}}_+ \subseteq G/P^-$. Let $z_0\in {{\mathfrak {z}}}({{\mathfrak {h}}}\cap {{\mathfrak {k}}})$ be the element determining the complex structure on $H/H\cap K$. Then $h=-iz_0$ is an Euler element in ${{\mathfrak {q}}}= i {{\mathfrak {h}}}$. Now (4.1) is the Harish–Chandra realization of $H/H\cap K$ as ${\mathcal {D}}_+$ in $G/P^-$ (see [60, p. 58] or [22, Ch. VII] for details).

Suppose that the complex conjugation $\tau$ of ${{\mathfrak {g}}}$ with respect to ${{\mathfrak {h}}}$ integrates to an involution $\tau ^G$ on *G*. This is the case if *G* is simply connected or if $G=\mathop {\textrm{Inn}}\nolimits {{{\mathfrak {g}}}}$. We then assume that $H = G^{\tau ^G}_e$. If *G* is simply connected, then $H = G^{\tau ^G}$ is connected and [24, Thm. VI.11] implies that $H=G^{{\mathcal {D}}_+}$, where $G^{{\mathcal {D}}_+}$ is the stabilizer of the base point ${\mathcal {D}}_+$.

But in general, if *G* is not simply connected, then $G^{{\mathcal {D}}_+}$ and $G^{\tau ^G}$ may differ.

As an example, consider $H=\mathop {\textrm{PSL}}\nolimits _2({{\mathbb {R}}})\subseteq G=\mathop {\textrm{PSL}}\nolimits _2({{\mathbb {C}}})\cong \mathop {\textrm{Inn}}\nolimits ({{\mathfrak {g}}})$ and note that $\tau ^G(g) = \tau g \tau$ in this case. Then

$$\begin{aligned} G^{\tau ^G} =\mathop {\textrm{PSL}}\nolimits _2({{\mathbb {C}}})^{\tau ^G} \cong \mathop {\textrm{PGL}}\nolimits _2({{\mathbb {R}}}) \cong \mathop {\textrm{Aut}}\nolimits (\mathop {{\mathfrak {sl} }}\nolimits _2({{\mathbb {R}}})),\end{aligned}$$

which is not connected because it also contains the image of $$\begin{pmatrix} i &{}\quad 0 \\ 0 &{}\quad -i \end{pmatrix}$$. The domain ${\mathcal {D}}_+ = H.i \subseteq {{\mathbb {C}}}_\infty$ (the Riemann sphere) is the upper half plane and the stabilizer subgroup of ${\mathcal {D}}_+$ is

$$\begin{aligned} G^{{\mathcal {D}}_+} = \mathop {\textrm{PSL}}\nolimits _2({{\mathbb {R}}}) \not = \mathop {\textrm{PGL}}\nolimits _2({{\mathbb {R}}}) = G^{\tau ^G}.\end{aligned}$$

The reflections in $\mathop {\textrm{GL}}\nolimits _2({{\mathbb {R}}})$ exchange the two open *H*-orbits.

#### Remark 4.12

The flag manifolds ${\mathcal {M}}= G/P^- \cong K/K^h$ appearing in this section are compact symmetric spaces on which the maximal compactly embedded subgroup $K \subseteq G$ acts by automorphisms. These spaces are called *symmetric R-spaces*.

Defining a symmetric R-space as a compact symmetric space ${\mathcal {M}}$ which is a real flag manifold, Loos shows in [37, Satz 1] that this implies the existence of an Euler element $h \in {\mathcal {E}}({{\mathfrak {g}}})$ such that ${\mathcal {M}}\cong G/P^-$, so that ${\mathcal {M}}\cong K/K\cap P^- = K/K^h$ as a Riemannian symmetric space (see [41, §7.2] for more details).

If *G* is hermitian of tube type, then ${\mathcal {M}}\cong K/K^h$ can be identified with the Šhilov boundary of the corresponding bounded symmetric domain ${\mathcal {D}}_G \cong G/K$, and this leads to a *G*-invariant causal structure on ${\mathcal {M}}$. As $\mathop {\textrm{dim}}\nolimits Z(K) = 1$, with respect to the *K*-action, we have a natural 1-parameter family of *K*-invariant Lorentzian structures on ${\mathcal {M}}$. They correspond to $K^h$-invariant Lorentzian forms on $T_{eK^h}({\mathcal {M}}) \cong {{\mathfrak {q}}}_{{\mathfrak {k}}}= {{\mathfrak {z}}}({{\mathfrak {k}}}) \oplus [{{\mathfrak {h}}}_{{\mathfrak {k}}},{{\mathfrak {q}}}_{{\mathfrak {k}}}]$ which are positive definite on ${{\mathfrak {z}}}({{\mathfrak {k}}})$ and negative definite on its orthogonal space $[{{\mathfrak {h}}}_{{\mathfrak {k}}},{{\mathfrak {q}}}_{{\mathfrak {k}}}]$.

## Observer domains associated with modular geodesics

In this section, we associate to any modular causal geodesic $\gamma$ in an ncc semisimple symmetric space $M= G/H$ an *observer domain* $W(\gamma )$. It is an open connected subset of *M* invariant under the centralizer $G^h$ of the corresponding causal Euler element *h*. We then show that, for $h \in C^\circ$, the domain $W(\gamma )$ coincides with the connected component $W \subseteq W_M^+(h)$ of the base point *eH* of the corresponding positivity domain. In Sect. 6, we show that $W_M^+(h)$ is connected for $G = \mathop {\textrm{Inn}}\nolimits ({{\mathfrak {g}}})$, which implies that $W = W_M^+(h)$ in this case.

### Definition 5.1

Let $(G,\tau ^G, H,C)$ be a non-compactly causal symmetric Lie group and $M = G/H$ be the corresponding ncc symmetric space. We assume that ${{\mathfrak {g}}}= {{\mathfrak {g}}}_s$, i.e., that $({{\mathfrak {g}}},\tau )$ is a direct sum of irreducible ncc symmetric Lie algebras.

(a) We write $\le$ for the order on *M* defined by the closed Olshanski semigroup $S = H \exp (C) = \exp (C)H$ which always exists because ${{\mathfrak {z}}}({{\mathfrak {g}}}) = \{0\}$ ([30, Thm. 3.1] or Theorem C.1in Appendix C) via

$$\begin{aligned} g_1H \le g_2 H \quad \text{ if } \quad g_1^{-1} g_2 \in SH/H = \mathop {\textrm{Exp}}\nolimits _{eH}(C) \end{aligned}$$

and write order intervals as

$$\begin{aligned}{}[x,y] = \{ z \in M : x \le z \le y\} = {\uparrow }x \cap {\mathop {\downarrow }}y, \end{aligned}$$

where

$$\begin{aligned} {\uparrow }x = \{ z \in M : x \le z\} \quad \text{ and } \quad {\mathop {\downarrow }}y = \{ z \in M : z \le y\}.\end{aligned}$$

(b) A subset $X\subseteq M$ is called *order convex* if

$$\begin{aligned}{}[a,b] \subseteq X \quad \text{ for } \quad a,b \in X.\end{aligned}$$

As the intersection of order convex subsets is order convex, we can defined the *order convex hull*

$$\begin{aligned} \mathop {\textrm{oconv}}\nolimits (D):= \bigcap \{ D' \subseteq M : D \subseteq D', D' \ \text{ order } \text{ convex } \}.\end{aligned}$$

Clearly $\mathop {\textrm{oconv}}\nolimits (D)$ is the smallest order convex subset of *X* containing *D*.

(c) For a modular geodesic $\gamma : {{\mathbb {R}}}\rightarrow M$, we call

$$\begin{aligned} W(\gamma ):= {\mathop {\downarrow }}\gamma ({{\mathbb {R}}}) \cap {\uparrow }\gamma ({{\mathbb {R}}}) = \bigcup _{t < s} [\gamma (t), \gamma (s)] \end{aligned}$$

the *observer domain associated to* $\gamma$. Note that this domain depends on the cone $C \subseteq {{\mathfrak {q}}}$ specifying the order on *M*.

### Lemma 5.2

The subset $W(\gamma )$ has the following properties:

- $W(\gamma )\subseteq M$ is open and connected.
- $$W(\gamma ) = \mathop {\textrm{oconv}}\nolimits (M^h_{eH})$$ for $$M^h_{eH} = \mathop {\textrm{Exp}}\nolimits _{eH}({{\mathfrak {q}}}_{{\mathfrak {p}}})$$.
- Suppose that $H_K = K^h$ and $C = C_{{\mathfrak {q}}}^{\textrm{max}}$ and identify $M = G/H$ with $M_{{\mathcal {D}}_+}$ (Proposition 4.8). Then
  5.1 $$\begin{aligned} W(\gamma ) = \{ g.{{\mathcal {D}}_+} : 0 \in g.{{\mathcal {D}}_+}, g.{{\mathcal {D}}_+} \ \text{ bounded }\} \end{aligned}$$
  and this domain is $G^h$-invariant.

### Proof

(a) To see that $W(\gamma )$ is open, we first observe that $\gamma (s) \in ({\uparrow }\gamma (t))^\circ$ for $t < s$. For real numbers $t_j \in {{\mathbb {R}}}$ with $t_1< t_2< t_3 < t_4$, this implies that

$$\begin{aligned}{}[\gamma (t_2), \gamma (t_3)] \subseteq [\gamma (t_1), \gamma (t_4)]^\circ .\end{aligned}$$

This shows that $W(\gamma )$ is open.

To see that $W(\gamma )$ is connected, we recall that the order on *M* is globally hyperbolic, in particular all order intervals [*x*, *y*] are compact. As all elements $z \in [x,y]$ lie on causal curves from *x* to *y* ([23, Thm. 4.29]), the order intervals are pathwise connected. As an increasing union of the order intervals $[\gamma (-n), \gamma (n)]$, the wedge domain $W(\gamma )$ is connected.

(b) Order intervals are convex and directed unions of convex sets of convex. Therefore,

$$\begin{aligned} W(\gamma ) = \bigcup _{t < s} [\gamma (t), \gamma (s)] \end{aligned}$$

is convex, whence $W(\gamma ) = \mathop {\textrm{oconv}}\nolimits (\gamma ({{\mathbb {R}}})).$

From the fact that *h* is central in ${{\mathfrak {h}}}_{{\mathfrak {k}}}+ {{\mathfrak {q}}}_{{\mathfrak {p}}}$, it easily follows that, in the symmetric space $M^h_{eH} = \mathop {\textrm{Exp}}\nolimits _{eH}({{\mathfrak {q}}}_{{\mathfrak {p}}})$ the geodesic line $\gamma ({{\mathbb {R}}})$ is cofinal in both directions because we have in ${{\mathfrak {q}}}$:

$$\begin{aligned} \bigcup _{s < t} (sh + C) \cap (th - C) \supseteq {{\mathfrak {q}}}.\end{aligned}$$

For $x \in {{\mathfrak {q}}}_{{\mathfrak {p}}}$, we thus find $s,t \in {{\mathbb {R}}}$ with $x \in sh + C^\circ$ and $x \in st - C^\circ$. Then

$$\begin{aligned} \mathop {\textrm{Exp}}\nolimits (sh)< \mathop {\textrm{Exp}}\nolimits (x) < \mathop {\textrm{Exp}}\nolimits (th) \end{aligned}$$

in $M^h_e$. This implies that

$$\begin{aligned} W(\gamma ) \supseteq \mathop {\textrm{Exp}}\nolimits _{eH}({{\mathfrak {q}}}_{{\mathfrak {p}}}) = M^h_{eH} = G^h_e.eH \supseteq \gamma ({{\mathbb {R}}}).\end{aligned}$$

This completes the proof.

(c) The modular group acts on ${\mathcal {B}}\cong N^+.eP^- \subseteq G/P^-$ by $\exp (th).x = e^tx$. Therefore $\gamma (t) = e^t {{\mathcal {D}}_+}$ enlarges ${{\mathcal {D}}_+}$ for $t > 0$ and shrinks ${{\mathcal {D}}_+}$ for $t < 0$ (Theorem 4.5). As $\gamma$ is strictly increasing, this implies that

$$\begin{aligned} {\mathop {\downarrow }}\gamma ({{\mathbb {R}}}) = \{ g.{{\mathcal {D}}_+} : (\exists t \in {{\mathbb {R}}}) g.{{\mathcal {D}}_+} \subseteq e^t {{\mathcal {D}}_+} \} = \{ g.{{\mathcal {D}}_+} : g.{{\mathcal {D}}_+}\ \text{ bounded } \text{ in } \ {\mathcal {B}}\}.\end{aligned}$$

Further

$$\begin{aligned} {\uparrow }\gamma ({{\mathbb {R}}}) = \{ g.{{\mathcal {D}}_+} : (\exists t \in {{\mathbb {R}}}) g.{{\mathcal {D}}_+} \supseteq e^t {{\mathcal {D}}_+} \} = \{ g.{{\mathcal {D}}_+} : 0 \in g.{{\mathcal {D}}_+}\},\end{aligned}$$

so that (5.1) follows. As any $g \in G^h = P^+ \cap P^-$ acts by linear maps on the Bruhat cell ${\mathcal {B}}\cong {{\mathfrak {g}}}_1(h)$, (5.1) implies that $G^h$ leaves the set $W(\gamma )$ of all bounded domains $g.{{\mathcal {D}}_+}$ containing 0 invariant. $\square$

### Example 5.3

(de Sitter space) We consider de Sitter space

$$\begin{aligned} M = \mathop {\textrm{dS}}\nolimits ^d = \{ (x_0,{{\textbf {x}}}) \in {{\mathbb {R}}}^{1,d} : \beta (x,x) = -1\}, \quad \text{ where } \quad \beta (x,y) = x_0 y_0 - {{\textbf {x}}}{{\textbf {y}}}\end{aligned}$$

is the canonical Lorentzian form on ${{\mathbb {R}}}^{1,d}$ (cf. Sect. D). Here

$$\begin{aligned} G = \mathop {\textrm{SO}}\nolimits _{1,d}({{\mathbb {R}}})^{\uparrow }= \mathop {\textrm{SO}}\nolimits _{1,d}({{\mathbb {R}}})_e, \quad H = G_{{{\textbf {e}}}_1} = \mathop {\textrm{SO}}\nolimits _{1,d-1}({{\mathbb {R}}})^{\uparrow }\end{aligned}$$

and

$$\begin{aligned} C \subseteq T_{{{\textbf {e}}}_1}(M) = {{\textbf {e}}}_1^\bot \quad \text{ given } \text{ by } \quad C = \{ (x_0,{{\textbf {x}}}) : x_1 = 0, x_0 \ge 0, x_0^2 \ge {{\textbf {x}}}^2\}.\end{aligned}$$

We claim that, for the modular geodesic

$$\begin{aligned} \gamma (t) = \cosh (t) {{\textbf {e}}}_1 + \sinh (t) {{\textbf {e}}}_0 = e^{th}{{\textbf {e}}}_1, \end{aligned}$$

we have

5.2 $$\begin{aligned} W(\gamma ) = W_{\mathop {\textrm{dS}}\nolimits ^d}(h) = \{ x \in \mathop {\textrm{dS}}\nolimits ^d : x_1 > |x_0|\} = W_R \cap \mathop {\textrm{dS}}\nolimits ^d, \end{aligned}$$

where $W_R = \{ (x_0,{{\textbf {x}}}) : x_1 > |x_0|\}$ (cf. Appendix D in [52]). As the right wedge $W_R \subseteq {{\mathbb {R}}}^{1,d}$ is causally complete, we clearly have $W(\gamma ) \subseteq W_R \cap \mathop {\textrm{dS}}\nolimits ^d = W_{\mathop {\textrm{dS}}\nolimits ^d}(h)$. For the converse inclusion, let $x \in W_R$. We have to find a $t \in {{\mathbb {R}}}$ with $x \le \gamma (t)$, i.e.,

$$\begin{aligned} x_0 < \gamma (t)_0 = \sinh (t) \end{aligned}$$

and

$$\begin{aligned} 0 < \beta (\gamma (t) -x, \gamma (t) -x) = (\sinh (t)-x_0)^2 - (\cosh (t)-x_1)^2 - x_2^2 - \cdots - x_d^2.\end{aligned}$$

Since $\beta (\gamma (t),\gamma (t)) = -1$, we obtain for the right hand side

$$\begin{aligned} \beta (\gamma (t)-x,\gamma (t)-x)= & {} \beta (\gamma (t), \gamma (t)) - 2 \beta (\gamma (t),x\big ) + \beta (x,x)\\= & {} -1 - 2\beta (\gamma (t),x)+ \beta (x,x).\end{aligned}$$

Further

$$\begin{aligned} -2 \beta (\gamma (t),x) = 2 x_1 \cosh (t) - 2 x_0 \sinh (t) \approx e^t (x_1 - x_0) \quad \text{ for } \quad t>\!\!> 0,\end{aligned}$$

and if $x_1 > |x_0|$, this expression is arbitrarily large for $t \rightarrow \infty$. This shows that $W_R \subseteq {\mathop {\downarrow }}\gamma ({{\mathbb {R}}})$, and we likewise see that $W_R \subseteq {\uparrow }\gamma ({{\mathbb {R}}})$.

### Proposition 5.4

If $H_K = K^h$ and $C = C_{{\mathfrak {q}}}^{\textrm{max}}$, then

- $$W(\gamma ) \subseteq W = W_M^+(h)_{eH}$$.
- $$h + {\mathcal {D}}_{{\mathfrak {g}}}\subseteq {{\mathfrak {h}}}+ C^\circ $$.

### Proof

If $gH \in W(\gamma ) \subseteq G/H$, then the corresponding subset $g.{{\mathcal {D}}_+} \subseteq {\mathcal {B}}$ is convex by Theorem 4.5, and it contains 0 by (5.1). Therefore the curve

$$\begin{aligned} \eta : {{\mathbb {R}}}\rightarrow M,\quad \eta (t):= \exp (th)gH \end{aligned}$$

is increasing because $t \mapsto e^t g.{{\mathcal {D}}_+}$ is an increasing family of subsets of ${\mathcal {B}}$. The invariance of the order thus implies that

$$\begin{aligned} g^{-1}.\eta '(0) = p_{{\mathfrak {q}}}(\mathop {\textrm{Ad}}\nolimits (g)^{-1}h) \in C_{{\mathfrak {q}}}^{\textrm{max}}.\end{aligned}$$

We also know that $g.{{\mathcal {D}}_+} \in P^+.{{\mathcal {D}}_+}$ (Theorem 4.5 and Lemma 5.2(c)), so that there exist $g_1 \in G^h$ and $y \in {{\mathfrak {g}}}_1(h)$ with $g.H = g_1 \exp (y).H$. Thus

5.3 $$\begin{aligned} \mathop {\textrm{Ad}}\nolimits (g)^{-1}h \in \mathop {\textrm{Ad}}\nolimits (H) e^{-\mathop {\textrm{ad}}\nolimits y}h \in {{\mathfrak {h}}}+ C_{{\mathfrak {q}}}^{\textrm{max}}, \end{aligned}$$

and therefore

$$\begin{aligned} e^{-\mathop {\textrm{ad}}\nolimits y}h = h - [y,h] = h + y \in {{\mathfrak {h}}}+ C_{{\mathfrak {q}}}^{\textrm{max}}.\end{aligned}$$

Recall the definition of ${\mathcal {D}}_{{\mathfrak {g}}}$ in (4.7). The condition

$$\begin{aligned}eP^- \in g.{{\mathcal {D}}_+} = g_1 \exp (y).{{\mathcal {D}}_+} = g_1.\exp (y + {\mathcal {D}}_{{\mathfrak {g}}})P^-\end{aligned}$$

is equivalent to $-y \in {\mathcal {D}}_{{\mathfrak {g}}}= - {\mathcal {D}}_{{\mathfrak {g}}}$, showing that

5.4 $$\begin{aligned} W(\gamma ) = G^h \exp ({\mathcal {D}}_{{\mathfrak {g}}}).{\mathcal {D}}_+ \subseteq M_{{\mathcal {D}}_+} \end{aligned}$$

(cf. Lemma 5.2(c)). We therefore derive from (5.3) that $h + {\mathcal {D}}_{{\mathfrak {g}}}\subseteq {{\mathfrak {h}}}+ C_{{\mathfrak {q}}}^{\textrm{max}},$ and since $h \in C_{{\mathfrak {q}}}^{\mathrm{max,\circ }}$ and ${{\mathcal {D}}_+}$ is starlike with respect to 0, we obtain

5.5 $$\begin{aligned} h + {\mathcal {D}}_{{\mathfrak {g}}}\subseteq {{\mathfrak {h}}}+ C_{{\mathfrak {q}}}^{\mathrm{max, \circ }}. \end{aligned}$$

We thus obtain $\mathop {\textrm{Ad}}\nolimits (g)^{-1}.h \in {{\mathfrak {h}}}+ C_{{\mathfrak {q}}}^{\mathrm{max,\circ }}$, i.e., $gH \in W_M^+(h)$. This shows that $W(\gamma ) \subseteq W_M^+(h)$, and the connectedness of $W(\gamma )$ (Lemma 5.2(a)) yields $W(\gamma ) \subseteq W$. $\square$

### Remark 5.5

From (5.4) it follows that, as a subset of *M*,

5.6 $$\begin{aligned} W(\gamma ) = G^h \exp ({\mathcal {D}}_{{\mathfrak {g}}}).H = G^h_e \exp ({\mathcal {D}}_{{\mathfrak {g}}}).H. \end{aligned}$$

For the quotient map $q : G \rightarrow G/H$, this means that

$$\begin{aligned} q^{-1}(W(\gamma )) = G^h \exp ({\mathcal {D}}_{{\mathfrak {g}}}) H \subseteq G.\end{aligned}$$

This is a $G^h\times H$-invariant domain in *G* specified by its intersection with the abelian subgroup $N^+ = \exp ({{\mathfrak {g}}}_1(h))$; see [41, Rem. 6.2].

Combined with Theorem 7.1, that asserts the connectedness of $W_M^+(h)$, the following result implies that $W_M^+(h) \subseteq W(\gamma )$.

### Proposition 5.6

If $H_K = K^h$ and $C = C_{{\mathfrak {q}}}^{\textrm{max}}$, then $W \subseteq W(\gamma )$.

### Proof

As both sides are $G^h_e$-invariant (Lemma 5.2), the Positivity Domain Theorem (Theorem 3.6) implies that we have to verify the inclusion

$$\begin{aligned} \mathop {\textrm{Exp}}\nolimits _{eH}(\Omega _{{{\mathfrak {q}}}_{{\mathfrak {k}}}}) \subseteq W(\gamma ).\end{aligned}$$

Invariance of both sides under $(H_K)_e$ and $\mathop {\textrm{Ad}}\nolimits ((H_K)_e){{\mathfrak {t}}}_{{\mathfrak {q}}}= {{\mathfrak {q}}}_{{{\mathfrak {k}}}}$ further reduce the problem to the inclusion

5.7 $$\begin{aligned} \mathop {\textrm{Exp}}\nolimits _{eH}(\Omega _{{{\mathfrak {t}}}_{{\mathfrak {q}}}}) \subseteq W(\gamma ). \end{aligned}$$

To this end, we use the Lie subalgebra ${{\mathfrak {l}}}\subseteq {{\mathfrak {g}}}$ generated by *h* and ${{\mathfrak {t}}}_{{\mathfrak {q}}}$ (Proposition 2.8). Then $[{{\mathfrak {l}}},{{\mathfrak {l}}}] \cong \mathop {{\mathfrak {sl} }}\nolimits _2({{\mathbb {R}}})^s$ and ${{\mathfrak {t}}}_{{\mathfrak {q}}}\cong \mathop {{\mathfrak {so} }}\nolimits _2({{\mathbb {R}}})^s$. This reduces the verification of the inclusion (5.7) to the case where ${{\mathfrak {g}}}= \mathop {{\mathfrak {sl} }}\nolimits _2({{\mathbb {R}}})^s$, ${{\mathfrak {h}}}= \mathop {{\mathfrak {so} }}\nolimits _{1,1}({{\mathbb {R}}})^s$ and ${{\mathfrak {t}}}_{{\mathfrak {q}}}\cong \mathop {{\mathfrak {so} }}\nolimits _2({{\mathbb {R}}})^s$.

As this is a product situation, it suffices to consider the case where

$$\begin{aligned} {{\mathfrak {g}}}= \mathop {{\mathfrak {sl} }}\nolimits _2({{\mathbb {R}}})\supseteq {{\mathfrak {h}}}= \mathop {{\mathfrak {so} }}\nolimits _{1,1}({{\mathbb {R}}}), \quad {{\mathfrak {t}}}_{{\mathfrak {q}}}= \mathop {{\mathfrak {so} }}\nolimits _2({{\mathbb {R}}}) \quad \text{ and } \quad h = \frac{1}{2}\begin{pmatrix} 1 &{}\quad 0 \\ 0 &{}\quad -1 \end{pmatrix}.\end{aligned}$$

By (5.6), we have to show that

5.8 $$\begin{aligned} \exp (tx) \in G^h_e \exp ({\mathcal {D}}_{{\mathfrak {g}}}) H \quad \text{ for } \quad |t| < \pi /2 \quad \text{ and } \quad x = \frac{1}{2} \begin{pmatrix} 0 &{}\quad -1 \\ 1 &{}\quad 0 \end{pmatrix}. \end{aligned}$$

We identify $\mathop {{\mathfrak {sl} }}\nolimits _2({{\mathbb {R}}})$ with 3-dimensional Minkowski space ${{\mathbb {R}}}^{1,2}$, via

$$\begin{aligned} {{\textbf {e}}}_0:= \frac{1}{2} \begin{pmatrix} 0 &{}\quad -1 \\ 1 &{}\quad 0 \end{pmatrix}, \quad {{\textbf {e}}}_1:= \frac{1}{2} \begin{pmatrix} 0 &{}\quad 1\\ 1 &{}\quad 0 \end{pmatrix}, \quad {{\textbf {e}}}_2:= h =\frac{1}{2} \begin{pmatrix} 1 &{}\quad 0\\ 0 &{}\quad -1 \end{pmatrix}.\end{aligned}$$

In the centerfree group $G:= \mathop {\textrm{Inn}}\nolimits ({{\mathfrak {g}}}) \cong \mathop {\textrm{SO}}\nolimits _{1,2}({{\mathbb {R}}})_e$, we have

$$\begin{aligned} K:= G_{{{\textbf {e}}}_0} \cong \mathop {\textrm{SO}}\nolimits _2({{\mathbb {R}}}) \quad \text{ and } \quad K^h = \{e\} = H_K,\end{aligned}$$

so that $H:= G_{{{\textbf {e}}}_1} = \exp ({{\mathfrak {h}}}_{{\mathfrak {p}}}) = \mathop {\textrm{SO}}\nolimits _{1,1}({{\mathbb {R}}})_e$ is connected. Therefore, $G/H \cong G.{{\textbf {e}}}_1 = \mathop {\textrm{dS}}\nolimits ^2$ (de Sitter space) and $\exp (tx)H$ corresponds to

$$\begin{aligned} \exp (tx).{{\textbf {e}}}_1= \cos (t){{\textbf {e}}}_1 + \sin (t) {{\textbf {e}}}_2.\end{aligned}$$

Now $|t| < \pi /2$ implies $\cos (t) > 0$, hence that

5.9 $$\begin{aligned} \exp (tx).{{\textbf {e}}}_1\in W_{\mathop {\textrm{dS}}\nolimits ^2}(\gamma ) \quad \text{ for } \quad \gamma (t) = \cos (t) {{\textbf {e}}}_1 + \sin (t) {{\textbf {e}}}_0 \end{aligned}$$

(Example 5.3). We write elements of ${{\mathcal {D}}_+}$ as $y = s e$, $|s| < 1$ (see Example 4.3). Then $\exp (y).{{\textbf {e}}}_1$ corresponds to

$$\begin{aligned} e^{\mathop {\textrm{ad}}\nolimits y}.\frac{1}{2}(e+f)&= \frac{1}{2}e^{\mathop {\textrm{ad}}\nolimits se}(e+f) = \frac{1}{2}(e+ e^{\mathop {\textrm{ad}}\nolimits se} f) = \frac{1}{2}\big (e+ f + s[e,f] + \frac{s^2}{2}[e,[e,f]]\big ) \\&= \frac{1}{2}(e+ f) + sh + \frac{s^2}{2}[e,h] = \frac{1}{2}(e+ f) + sh - \frac{s^2}{2}e, \end{aligned}$$

so that

$$\begin{aligned} \exp (y).{{\textbf {e}}}_1 = {{\textbf {e}}}_1 + s {{\textbf {e}}}_2 - \frac{s^2}{2}({{\textbf {e}}}_1 - {{\textbf {e}}}_0) =\Big (\frac{s^2}{2}, 1 - \frac{s^2}{2}, s\Big ).\end{aligned}$$

This element lies in the wedge domain $W_{\mathop {\textrm{dS}}\nolimits ^2}(h)$ if and only if $1-s^2/2 > s^2/2$ (Example 5.3), which is equivalent to $|s| < 1$. Then its $G^h_e$-orbit contains the element $(0, \sqrt{1-s^2}, s)$. For $|t| < \pi /2$, the element $\exp (tx).{{\textbf {e}}}_1$ is of this form, showing that $\exp (tx) \in G^h \exp (y) H$. This completes the proof. $\square$

Combining the preceding two propositions, we get the main result of this section. It shows that the observer domain $W(\gamma )$ coincides with a connected component of the positivity domain $W_M^+(h)$. This result provides two complementary perspectives on this domain.

### Theorem 5.7

(Observer Domain Theorem) Let $({{\mathfrak {g}}},\tau ,C)$ be a non-compactly causal semisimple symmetric Lie algebra with causal Euler element $h \in C^\circ \cap {{\mathfrak {q}}}_{{\mathfrak {p}}}$ with $\tau = \tau _h \theta$ and let *G* be a connected Lie group with Lie algebra ${{\mathfrak {g}}}$ and $H:= K^h \exp ({{\mathfrak {h}}}_{{\mathfrak {p}}})$. If $C = C_{{\mathfrak {q}}}^{\textrm{max}}$, then $W = W(\gamma )$.

We can even extend this result to coverings:

### Corollary 7.2

If $H' \subseteq H = K^h\exp ({{\mathfrak {h}}}_{{\mathfrak {p}}})$ is an open subgroup and $C = C_{{\mathfrak {q}}}^{\textrm{max}}$, then $W':= W_{M'}^+(h)_{eH'} = W({\widetilde{\gamma }})$ holds in $M'= G/H'$ for ${\widetilde{\gamma }}(t):= \mathop {\textrm{Exp}}\nolimits _{eH'}(th)$.

### Proof

Let $q : M' = G/H' \rightarrow G/H \cong M_{{\mathcal {D}}_+}$ be the canonical equivariant covering from [41, Lemma 7.11].

First we show that $W' \subseteq M'$ is order convex. So let $x\le y \le z$ in $M'$ with $x,z \in W'$ and let $\eta : [0,2] \rightarrow M'$ be a causal curve with

$$\begin{aligned} \eta (0) =x, \quad \eta (1) = y, \quad \eta (2) = z.\end{aligned}$$

Then $q(\eta (t)) \in [q(x),q(z)] \subseteq W$ for $t \in [0,2]$ holds because $W = W(\gamma )$ is order convex in *M*.

As *W* is contractible by Theorem3.6(b), it is in particular simply connected. Therefore, $q^{-1}(W)$ is a disjoint union of open subsets $(W_j')_{j \in J}$ mapped by *q* diffeomorphically onto *W*. By definition, $W'$ is one such connected component, so that

$$\begin{aligned} q_W:= q\vert _{W'} : W' \rightarrow W \end{aligned}$$

is a diffeomorphism. Therefore $\eta$ is the unique continuous lift of $q \circ \eta$ in $M'$, hence contained in $W'$. This implies that $y \in W'$, so that $W'$ is order convex.

As $q_W : W' \rightarrow W$ is an isomorphism of causal manifolds, it also is an order isomorphism. Finally $W(\gamma ) = \mathop {\textrm{oconv}}\nolimits (\gamma ({{\mathbb {R}}})) = W$ implies that $W'(\gamma ) = \mathop {\textrm{oconv}}\nolimits ({\widetilde{\gamma }}({{\mathbb {R}}})) = W'$. $\square$

### Remark 5.9

It is not clear to which extent $W(\gamma )$ depends on the specific cone *C*. In particular it would be interesting to see if the minimal and maximal cones lead to the same domain $W(\gamma )$. We have already seen that the positivity domain $W_M^+(h)$ depends non-trivially on the cone *C* ([41, Ex. 6.8]) so one may expect that this is also the case for $W(\gamma )$.

### Lemma 5.10

The involution $\tau _M$ on *M* defined by $\tau _M(gH) = \tau ^G(g)H$ satisfies

5.10 $$\begin{aligned} \tau _M(W_M^+(h)) = W_M^+(h) \quad \text{ and } \quad \tau _M(W(\gamma )) = W(\gamma ). \end{aligned}$$

### Proof

(a) The condition $gH \in W_M^+(h)$ is equivalent to $\mathop {\textrm{Ad}}\nolimits (g)^{-1}h \in {\mathcal {T}}_C$ by (5.10), and this implies that

$$\begin{aligned} \mathop {\textrm{Ad}}\nolimits (\tau (g))^{-1}(-h) = \tau (\mathop {\textrm{Ad}}\nolimits (g)^{-1}h) \in \tau ({\mathcal {T}}_C) = -{\mathcal {T}}_C,\end{aligned}$$

so that $\mathop {\textrm{Ad}}\nolimits (\tau (g))^{-1}h \in {\mathcal {T}}_C$, i.e., $\tau _M(gH) \in W_M^+(h)$. As $\tau _M$ is an involution, it follows that $\tau _M(W_M^+(h)) = W_M^+(h)$.

(b) As $\tau (C) = - C$, the involution $\tau _M$ reverses the causal structure on *M*. Moreover, $\tau _M(\gamma (t)) = \gamma (-t)$, so that

$$\begin{aligned} \tau _M(W(\gamma )) = \bigcup _{t< s} [\tau _M(\gamma (s)), \tau _M(\gamma (t))] = \bigcup _{t < s} [\gamma (-s), \gamma (-t)] = W(\gamma ). \end{aligned}$$

$$\square $$

We have seen above that, for the modular geodesic $\gamma (t) = \mathop {\textrm{Exp}}\nolimits _{eH}(th)$ in *M*, we have $W(\gamma ) = W$. The modular geodesic $\gamma$ is a specific orbit of the modular flow inside *W*. Now we show that all other $\alpha$-orbits in *W* lead to the same “observer domain”.

### Proposition 5.11

Let $m \in W$ and consider the curve

$$\begin{aligned} \beta : {{\mathbb {R}}}\rightarrow W, \quad \beta (t) = \alpha _t(m) = \exp (th).m.\end{aligned}$$

Then

5.11 $$\begin{aligned} W = W(\beta ) = \bigcup _{s < t} [\beta (s), \beta (t)]. \end{aligned}$$

### Proof

Using the subset realization of $M = G/H$ as $M_{{\mathcal {D}}_+} = \{ g.{\mathcal {D}}_+ : g \in G \}$ from Proposition 4.8, we have

$$\begin{aligned} W(\gamma ) = \{ g {\mathcal {D}}_+ : 0 \in g.{\mathcal {D}}_+, g.{\mathcal {D}}_+\ \text{ bounded } \text{ in } \exp ({{\mathfrak {g}}}_1(h)).P^-\}\end{aligned}$$

(Lemma 5.2(c)) and $W = W(\gamma )$ by Theorem 5.7. So we can write

$$\begin{aligned} \beta (t) = e^t.{\mathcal {D}}' \quad \text{ for } \text{ some } \quad {\mathcal {D}}' \in W(\gamma ).\end{aligned}$$

As $\beta ({{\mathbb {R}}}) \subseteq W(\gamma )$, the order convex hull $W(\beta )$ of $\beta ({{\mathbb {R}}})$ is contained in $W(\gamma ) = W$. To verify the converse inclusion, let ${\mathcal {D}}'' \in W$. Then $0 \in {\mathcal {D}}''$, and since ${\mathcal {D}}'$ is bounded, there exists a $t \in {{\mathbb {R}}}$ with $\beta (t) \subseteq {\mathcal {D}}''$. Likewise the boundedness of ${\mathcal {D}}''$ implies the existence of some $s \in {{\mathbb {R}}}$ with ${\mathcal {D}}'' \subseteq \beta (s)$. Hence, ${\mathcal {D}}'' \in [\beta (t),\beta (s)] \subseteq W(\beta )$. This shows that $W \subseteq W(\beta )$, and hence equality in (5.11). $\square$

### Remark 5.12

A similar result also holds in Minkowski space. If

$${{\textbf {x}}}\in W_R = \{ {{\textbf {y}}}\in {{\mathbb {R}}}^{1,d} : y_1 > |y_0| \}$$

and

$$\begin{aligned} \beta (t) = e^{th} {{\textbf {x}}}= (\cosh (t) x_0 + \sinh (t) x_1, \cosh (t) x_1 + \sinh (t) x_0, x_2, \ldots , x_d),\end{aligned}$$

then any other element ${{\textbf {y}}}\in W_R$ satisfies ${{\textbf {y}}}\in [\beta (t), \beta (s)]$ for suitable $t < s$, i.e., ${{\textbf {y}}}- \beta (t) \in V_+$ and $\beta (s) - {{\textbf {y}}}\in V_+$. In fact, $\beta (t)_0 \sim e^t(x_0 + x_1) \rightarrow \infty$ for $t \in \infty$ and $\beta (t_0) \sim e^{-t}(x_0 - x_1) \rightarrow -\infty$ for $t \rightarrow -\infty$. Moreover, for $s \rightarrow \infty$

$$\begin{aligned}&(\cosh (s) x_0 + \sinh (s) x_1 - y_0)^2 - (\cosh (s) x_1 + \sinh (s) x_0 - y_1)^2 \\&\sim \Big (e^s \frac{x_0 + x_1}{2} -y_0\Big )^2 - \Big (e^s \frac{x_0 + x_1}{2} -y_1\Big )^2 \sim e^s(x_0 + x_1)(y_1-y_0) \rightarrow \infty \end{aligned}$$

and, for $$t \rightarrow -\infty $$,

$$\begin{aligned}&(\cosh (t) x_0 + \sinh (t) x_1 - y_0)^2 - (\cosh (t) x_1 + \sinh (t) x_0 - y_1)^2 \\&\sim \Big (e^{-t} \frac{x_0 - x_1}{2} -y_0\Big )^2 - \Big (e^{-t} \frac{x_1 -x_0}{2} -y_1\Big )^2 \sim e^{-t} (x_0 - x_1) (-y_0) - e^{-t}(x_1 - x_0) (-y_1) \\&\quad = e^{-t} (x_1 - x_0) (y_0 + y_1) \rightarrow \infty . \end{aligned}$$

This shows that $W(\beta ) = W_R$ for all integral curves of the modular flow in $W_R$.

### Remark 5.13

On the de Sitter space $M = \mathop {\textrm{dS}}\nolimits ^d \subseteq {{\mathbb {R}}}^{1.d}$, the involution $\tau _h$ can be implemented naturally by

$$\begin{aligned} \tau _{h,M}(x) = (-x_0, -x_1, x_2, \ldots , x_d). \end{aligned}$$

This involution does not fix the base point ${{\textbf {e}}}_1$, it reverses the causal structure and it commutes with modular flow. Accordingly, we have the relation

$$\begin{aligned} \tau _{h,M}(W^+(h)) = W^+(-h).\end{aligned}$$

As we shall see in the next section, such a relation can only be realized because $-h \in \mathop {\textrm{Ad}}\nolimits (G)h$, i.e., the direction of the boost can be reversed by an element of *G*. If $-h \not \in \mathop {\textrm{Ad}}\nolimits (G)$ (*h* is not symmetric), then we shall see in Corollary 6.3 below that $W^+(-h) = \emptyset$, so that there is no involution on *M* mapping $W^+(h)$ to $W^+(-h)$.

However, as $\tau _h = \tau \theta$ (as involutions on ${{\mathfrak {g}}}$), and there are natural implementations $\tau _M$ and $\theta _M$ on $M = G/H$, both fixing the base points, the involution $\tau _M\theta _M$ implements the involution $\tau _h$ on *M* and fixes the base point, but it also fixes the wedge region

$$\begin{aligned} \tau _M\theta _M(W^+(h)) = W^+(h) \end{aligned}$$

because it preserves *h* and the causal structure. This is not desirable because we would prefer that $\tau _h$ maps $W^+(h)$ to some “opposite” wedge region (cf. [38]). Possible ways to resolve this problem and ideas how to implement locality conditions on non-compactly causal symmetric spaces are briefly discussed in [41, §4.3].

## Existence of positivity domains for Euler elements

In this section, we show that, for the maximal cone $C= C_{{\mathfrak {q}}}^\textrm{max}$ and a simple Lie algebra ${{\mathfrak {g}}}$, the real tube domain ${\mathcal {T}}_C = {{\mathfrak {h}}}+ C^\circ$ intersects the set ${\mathcal {E}}({{\mathfrak {g}}})$ of Euler elements in a connected subset (Theorem 6.1). This implies that, for an Euler element $h' \in {{\mathfrak {g}}}$, the positivity domain $W_M^+(h')$ is non-empty if and only if $h'$ and *h* are conjugate (Corollary 6.3).

### Theorem 6.1

Suppose that $({{\mathfrak {g}}},\tau ,C)$ is an irreducible simple ncc symmetric Lie algebra with $C = C^{\textrm{max}}_{{\mathfrak {q}}}$, ${\mathcal {T}}_C:= {{\mathfrak {h}}}+ C^\circ$, $G = \mathop {\textrm{Inn}}\nolimits ({{\mathfrak {g}}})$, $H = K^h \exp ({{\mathfrak {h}}}_{{\mathfrak {p}}})$ and $M = G/H$. Then ${\mathcal {E}}({{\mathfrak {g}}}) \cap {\mathcal {T}}_C$ is connected and a subset of ${\mathcal {O}}_h$. More precisely,

6.1 $$\begin{aligned} {\mathcal {E}}({{\mathfrak {g}}}) \cap {\mathcal {T}}_C = {\mathcal {O}}_h \cap {\mathcal {T}}_C = \mathop {\textrm{Ad}}\nolimits (H_e)(h + {\mathcal {D}}_{{\mathfrak {g}}}), \end{aligned}$$

where ${\mathcal {D}}_{{\mathfrak {g}}}= \{ u \in {{\mathfrak {g}}}_1(h) : \Vert u\Vert < 1\}$ is the open unit ball for which $\exp ({\mathcal {D}}_{{\mathfrak {g}}}) P^- = H.P^- \subseteq G/P^-.$

### Proof

We recall from Proposition 4.8 the open subsets ${\mathcal {D}}_\pm := H.eP^{\mp }\subseteq G/P^{\mp }$ which are the open orbits of the base point under $H = K^h \exp ({{\mathfrak {h}}}_{{\mathfrak {p}}})$. Then

$$\begin{aligned} \mathop {\textrm{comp}}\nolimits ({\mathcal {D}}_\pm ) = H \exp ({\mp } C) \end{aligned}$$

follows from Proposition 4.8, applied to the causal Euler element *h* and its negative. These semigroups have the Lie wedges

$$\begin{aligned} \mathop {{\textbf {L}}}\nolimits (\mathop {\textrm{comp}}\nolimits ({\mathcal {D}}_\pm )) = {{\mathfrak {h}}}{\mp } C. \end{aligned}$$

Let $x \in {\mathcal {E}}({{\mathfrak {g}}}) \cap {\mathcal {T}}_C$ for ${\mathcal {T}}_C = {{\mathfrak {h}}}+ C^\circ = \mathop {{\textbf {L}}}\nolimits (\mathop {\textrm{comp}}\nolimits ({\mathcal {D}}_-))^\circ$. We then have $s_t:= \exp (tx) \in \mathop {\textrm{comp}}\nolimits ({\mathcal {D}}_-)^\circ$ for $t > 0$. We conclude that $s_t(\overline{{\mathcal {D}}_-}) \subseteq {\mathcal {D}}_-$ and that there exists a complete metric on ${\mathcal {D}}_-$ for which each $s_t$ is a strict contraction (cf. [48, Thm. II.4]),^5^ so that the Banach Fixed Point Theorem implies the existence of a unique attracting fixed point $m_- \in {\mathcal {D}}_-$ for the vector field $X^{G/P^+}_x \in {\mathcal {V}}(G/P^+)$ defined by *x*. We now have

$$\begin{aligned} m_- \in {\mathcal {D}}_- = H.eP^+ = H_e.eP^+.\end{aligned}$$

Hence there exists $g_1 \in H_e$ with $g_1.m_- = eP^+$, and thus

6.2 $$\begin{aligned} y:= \mathop {\textrm{Ad}}\nolimits (g_1) x \in {{\mathfrak {p}}}^+ = {{\mathfrak {g}}}_1(h) \rtimes {{\mathfrak {g}}}_0(h). \end{aligned}$$

Then $y \in {\mathcal {T}}_C \cap {{\mathfrak {p}}}^+$ is an Euler element, and a similar argument shows that the vector field $X^{G/P^-}_y$ has a unique repelling fixed point $m_+ \in {\mathcal {D}}_+$. So $m_+ = \exp (-z) P^-$ for some $z \in {{\mathfrak {g}}}_1(h)$, and $\exp (z).m_+ = eP^-$. Hence the base point $eP^-\in G/P^-$ is a repelling fixed point of the Euler element $y':= e^{\mathop {\textrm{ad}}\nolimits z} y \in {{\mathfrak {g}}}_0(h)$, and $eP^+$ is an attracting fixed point in $G/P^+$. The attracting and repelling properties of the fixed points imply that

$$\begin{aligned} {{\mathfrak {g}}}_1(h) \subseteq {{\mathfrak {g}}}_1(y') \quad \text{ and } \quad {{\mathfrak {g}}}_{-1}(h) \subseteq {{\mathfrak {g}}}_{-1}(y'), \end{aligned}$$

so that we also have

$$\begin{aligned} {{\mathfrak {g}}}_0(h) = [{{\mathfrak {g}}}_1(h), {{\mathfrak {g}}}_{-1}(h)] \subseteq {{\mathfrak {g}}}_0(y').\end{aligned}$$

As *h* and $y'$ are Euler elements, this entails that ${{\mathfrak {g}}}_\lambda (h) = {{\mathfrak {g}}}_\lambda (y')$ for $\lambda = -1,0,1$. This shows that $\mathop {\textrm{ad}}\nolimits h = \mathop {\textrm{ad}}\nolimits y'$ and hence that $y' = h$ because ${{\mathfrak {z}}}({{\mathfrak {g}}}) = \{0\}$.

We conclude that

$$\begin{aligned} x = \mathop {\textrm{Ad}}\nolimits (g_1)^{-1} y = \mathop {\textrm{Ad}}\nolimits (g_1)^{-1} e^{-\mathop {\textrm{ad}}\nolimits z} h \quad \text{ with } \quad g_1 \in H_e, z \in {\mathcal {D}}_{{\mathfrak {g}}}.\end{aligned}$$

Conversely, we have seen in Proposition 5.4 that

6.3 $$\begin{aligned} e^{\mathop {\textrm{ad}}\nolimits {\mathcal {D}}_{{\mathfrak {g}}}} h = h + {\mathcal {D}}_{{\mathfrak {g}}}\subseteq {\mathcal {T}}_C. \end{aligned}$$

We finally obtain (6.1). $\square$

### Remark 6.2

Note that the preceding proof is based on the natural embedding

$$\begin{aligned} {\mathcal {O}}_h \cong G/G_h \rightarrow G/P^- \times G/P^+ \end{aligned}$$

which maps the Euler element $\mathop {\textrm{Ad}}\nolimits (g)H$ to $(m_+,m_-)$, where $m_+$ is the unique repelling fixed point of the flow defined by *h* in $G/P^-$ and $m_- \in G/P^+$ is the unique attracting fixed point.

### Corollary 8.3

(The set of positivity domains in *M*) If $h_1 \in {\mathcal {E}}({{\mathfrak {g}}})$ is an Euler element for which the positivity domain

$$\begin{aligned} W_M^+(h_1) = \{ m \in M = G/H : X^M_{h_1}(m) \in C_m^\circ \} \end{aligned}$$

is non-empty, then there exists a $g \in G$ with $h_1 = \mathop {\textrm{Ad}}\nolimits (g)h$ and

$$\begin{aligned} W_M^+(h_1) = g.W_M^+(h).\end{aligned}$$

### Proof

As $X^M_{h_1}(g_1H) \in C_{g_1H}^\circ$ is equivalent to $\mathop {\textrm{Ad}}\nolimits (g_1)^{-1} h_1 \in {{\mathfrak {h}}}+ C^\circ$ by (see Lemma 3.3), Theorem 6.1 implies that $h_1 = \mathop {\textrm{Ad}}\nolimits (g) h\in {\mathcal {O}}_h$ for some $g \in G$. The relation $W_M^+(h_1) = g.W_M^+(h)$ now follows directly from the definitions. $\square$

The preceding corollary shows that any wedge domain of the type $W_M^+(h_1)\subseteq M$, $h_1 \in {\mathcal {E}}({{\mathfrak {g}}})$, is a *G*-translate of the wedge domain $W_M^+(h)$, where $h \in C^\circ \cap {{\mathfrak {q}}}_{{\mathfrak {p}}}$ is a causal Euler element. So the action of *G* on the “wedge space” ${\mathcal {W}}(M)$ of *M* is transitive.

### Corollary 8.4

If the causal Euler element *h* is not symmetric, then $W_M^+(-h) = \emptyset$.

### Remark 6.5

(Extensions to the non-simple case) If $({{\mathfrak {g}}},\tau )$ is a direct sum of irreducible ncc symmetric Lie algebra $({{\mathfrak {g}}}_j,\tau _j)$ and $h = \sum _j h_j$ accordingly, then

$$\begin{aligned} C_{{\mathfrak {q}}}^{\textrm{max}}(h) = \prod _j C_{{{\mathfrak {q}}}_j}^{\textrm{max}}(h_j) \end{aligned}$$

(cf. (2.3)). Projecting to the ideals ${{\mathfrak {g}}}_j$, we obtain with Theorem 6.1 for $C = C_{{\mathfrak {q}}}^{\textrm{max}}(h)$ and $C_j = C_{{{\mathfrak {q}}}_j}^{\textrm{max}}(h_j)$ the relation

6.4 $$\begin{aligned} {\mathcal {E}}({{\mathfrak {g}}}) \cap {\mathcal {T}}_C \subseteq \prod _j {\mathcal {E}}({{\mathfrak {g}}}_j) \cap {\mathcal {T}}_{C_j} \subseteq \prod _j {\mathcal {O}}_{h_j} = {\mathcal {O}}_h. \end{aligned}$$

Further,

$$\begin{aligned} {\mathcal {O}}_h \cap {\mathcal {T}}_C = \prod _j {\mathcal {O}}_{h_j} \cap {\mathcal {T}}_{C_j} \end{aligned}$$

and ${\mathcal {D}}_{{\mathfrak {g}}}= \prod _j {\mathcal {D}}_{{{\mathfrak {g}}}_j}$ imply (6.1) for this case.

Note that the situation corresponds to ${{\mathfrak {g}}}= {{\mathfrak {g}}}_s$ (see (2.2)). In the general situation, where we assume only that all ideals of ${{\mathfrak {g}}}$ contained in ${{\mathfrak {h}}}$ are compact, we have

$$\begin{aligned} {{\mathfrak {g}}}= {{\mathfrak {g}}}_k \oplus {{\mathfrak {g}}}_r \oplus {{\mathfrak {g}}}_s,\end{aligned}$$

where ${{\mathfrak {g}}}_k \subseteq {{\mathfrak {h}}}$ is compact, ${{\mathfrak {g}}}_r$ is a direct sum of Riemannian symmetric Lie algebras and ${{\mathfrak {g}}}_s$ is a direct sum of irreducible ncc symmetric Lie algebras. All Euler elements are contained in ${{\mathfrak {g}}}_r + {{\mathfrak {g}}}_s$. If ${{\mathfrak {g}}}$ is only reductive, we assume ${{\mathfrak {z}}}({{\mathfrak {g}}}) \subseteq {{\mathfrak {g}}}^{-\theta }$, so that ${{\mathfrak {z}}}({{\mathfrak {g}}}) \subseteq {{\mathfrak {g}}}_r$. Then $h = h_r + h_s$ and

$$\begin{aligned} C_{{\mathfrak {q}}}^{\textrm{max}}(h)= {{\mathfrak {q}}}_r \oplus C_{{{\mathfrak {q}}}_s}^{\textrm{max}}(h_s).\end{aligned}$$

We conclude that

$$\begin{aligned}{\mathcal {E}}({{\mathfrak {g}}}) \cap {\mathcal {T}}_C \subseteq {\mathcal {E}}({{\mathfrak {g}}}) \cap {\mathcal {T}}_{C_{{\mathfrak {q}}}^{\textrm{max}}(h)} = \big ( ({\mathcal {E}}({{\mathfrak {g}}}_r)\cup \{0\}) \times {\mathcal {E}}({{\mathfrak {g}}}_s)\big ) \cap {\mathcal {T}}_{C_{{{\mathfrak {q}}}_s}^{\textrm{max}}(h_s)}\subseteq ({\mathcal {E}}({{\mathfrak {g}}}_r)\cup \{0\}) \times {\mathcal {O}}_{h_s}.\end{aligned}$$

This shows that, for any Euler element $k \in {{\mathfrak {g}}}$ with $W_M^+(k) \not =\emptyset$ we must have $k_s \in {\mathcal {O}}_{h_s}$, but there is no restriction on the Riemannian component $k_r \in {\mathcal {E}}({{\mathfrak {g}}}_r)$.

## Connectedness of the positivity domain

In this section, we show that if $G \cong \mathop {\textrm{Inn}}\nolimits ({{\mathfrak {g}}})$ is the adjoint group, then the positivity domain $W_M^+(h)$ is connected. This contrasts the situation for compactly causal symmetric spaces, where wedge regions are in general not connected. A typical example is anti-de Sitter spacetime (cf. [52, Lemma 11.2]).

### Theorem 7.1

(Connectedness of positivity domains) Suppose that $({{\mathfrak {g}}},\tau ,C)$ is an irreducible simple ncc symmetric Lie algebra with $C = C^\textrm{max}_{{\mathfrak {q}}}$ and the causal Euler element $h \in C^\circ \cap {{\mathfrak {q}}}_{{\mathfrak {p}}}$. Let $M = G/H$ for $G = \mathop {\textrm{Inn}}\nolimits ({{\mathfrak {g}}})$ and $H = K^h \exp ({{\mathfrak {h}}}_{{\mathfrak {p}}})$. Then the positivity domain $W_M^+(h)$ is connected.

### Proof

From Theorem 6.1 we derive that

$$\begin{aligned} G^+(h):= \{ g \in G : \mathop {\textrm{Ad}}\nolimits (g)^{-1}h \in {\mathcal {T}}_C \} = G^h \exp ({\mathcal {D}}_{{\mathfrak {g}}}) H_e, \end{aligned}$$

and this leads with Lemma 3.3 to

$$\begin{aligned} W_M^+(h) = G^+(h).eH = G^h \exp ({\mathcal {D}}_{{\mathfrak {g}}}).eH.\end{aligned}$$

Since $G^h$ has at most two connected components, this set is either connected or has two connected components ([41, Thm. 7.8]). As $G^h = K^h \exp ({{\mathfrak {q}}}_{{\mathfrak {p}}})$, we have $G^h = K^h G^h_e$, and $\mathop {\textrm{Ad}}\nolimits (K^h)$ preserves the open unit ball in ${{\mathfrak {g}}}_1(h)$. We thus derive from $K^h = H_K$:

$$\begin{aligned} W_M^+(h)&= G^h \exp ({\mathcal {D}}_{{\mathfrak {g}}}).eH = G^h_e K^h\exp ({\mathcal {D}}_{{\mathfrak {g}}}).eH = G^h_e \exp ({\mathcal {D}}_{{\mathfrak {g}}}) K^h.eH = G^h_e \exp ({\mathcal {D}}_{{\mathfrak {g}}}).eH, \end{aligned}$$

which is connected. $\square$

### Corollary 3.7

$$W(\gamma ) = W_M^+(h)$$.

### Proposition 7.3

(The stabilizer group of the observer domain) If ${{\mathfrak {g}}}= {{\mathfrak {g}}}_s$, then $G^h$ coincides with the stabilizer group

$$\begin{aligned} G_{W(\gamma )}:= \{ g \in G : g W(\gamma ) = W(\gamma )\} \end{aligned}$$

of the observer domain $W(\gamma ) \subseteq M = G/H$.

### Proof

We work with the subset realization of $M = G/H$ as $M_{{\mathcal {D}}_+} = \{ g.{\mathcal {D}}_+ : g \in G \}$ from Proposition 4.8. Then

$$\begin{aligned} W(\gamma ) = \{ g {\mathcal {D}}_+ : 0 \in g.{\mathcal {D}}_+, g.{\mathcal {D}}_+\ \text{ bounded } \text{ in } \exp ({{\mathfrak {g}}}_1(h)).P^-\}\end{aligned}$$

(Lemma 5.2(c)). Since $\exp ({{\mathbb {R}}}h)$ acts on $\exp ({{\mathfrak {g}}}_1(h))$ by dilations, it follows that

7.1 $$\begin{aligned} \bigcap _{g{\mathcal {D}}_+\in W(\gamma )} g{\mathcal {D}}_+ = \bigcap _{t \in {{\mathbb {R}}}} e^t {\mathcal {D}}_+ = \{eP^-\}. \end{aligned}$$

Therefore, $g W(\gamma ) = W(\gamma )$ for the action of *g* on $G/H \subseteq {{\mathbb {P}}}(G/P^-)$ implies that *g* preserves the intersection $\{eP^-\}$ of all subsets contained in $W(\gamma )$. This shows that *g* fixes $eP^-$, so that $g \in P^-$.

Next we recall that the involution $\tau _M$ on *M* defined by $\tau _M(gH) = \tau (g)H$ leaves $W(\gamma )$ invariant (Lemma 5.10), and this leads to

$$\begin{aligned} G_{W(\gamma )} = \tau (G_{W(\gamma )}) \subseteq P^- \cap \tau (P^-) = P^- \cap P^+ = G^h. \end{aligned}$$

$$\square $$

The preceding proposition shows that the set ${\mathcal {W}}= {\mathcal {W}}(M)$ of wedge domains in $M = G/H$ coincides with

7.2 $$\begin{aligned} {\mathcal {W}}= G.W(\gamma ) \cong G/G^h \cong {\mathcal {O}}_h. \end{aligned}$$

In particular, it is a symmetric space. Recall that, by Corollary 7.2, the observer domain coincides with the positivity domain $W_M^+(h)$.

## KMS wedge regions

With the structural results obtained so far, we have good control over the positivity domains $W_M^+(h)$ in ncc symmetric spaces $M = G/H$. So one may wonder if they also have an interpretation in terms of a KMS like condition. In [52], this has been shown for modular flows with fixed points, using such a fixed point as a base point. In this section we extend the characterization of the wedge domain *W* in terms of a geometric KMS condition to general ncc spaces.

To simplify references, we list our assumptions and the relevant notation below:

- $${{\mathfrak {g}}}$$ is simple,
- $G= \mathop {\textrm{Inn}}\nolimits ({{\mathfrak {g}}}) \subseteq G_{{\mathbb {C}}}= \mathop {\textrm{Inn}}\nolimits ({{\mathfrak {g}}}_{{\mathbb {C}}})_e$ (by (GP) and (Eff), [52, Lemma 2.12])
- $\sigma : G_{{\mathbb {C}}}\rightarrow G_{{\mathbb {C}}}$ denotes the complex conjugation with respect to *G*.
- $H = G^c \cap G$, where $G^c = (G_{{\mathbb {C}}}^{\overline{\tau }})_e$ and $K_{{\mathbb {C}}}\subseteq G_{{\mathbb {C}}}^\theta$ is an open subgroup. Note that $H \subseteq G^\tau$.
- $$\Xi = G.\mathop {\textrm{Exp}}\nolimits _{eK}(i\Omega _{{\mathfrak {p}}}) \subseteq G_{{\mathbb {C}}}/K_{{\mathbb {C}}}$$.
- $H_{{\mathbb {C}}}\subseteq G^\tau _{{\mathbb {C}}}$ is open with $G \cap H_{{\mathbb {C}}}= H$ (see §5), so that $M = G/H \hookrightarrow G_{{\mathbb {C}}}/H_{{\mathbb {C}}}$.
- $\tau _h^G(H_{{\mathbb {C}}}) = H_{{\mathbb {C}}}$ for the holomorphic involution of $G_{{\mathbb {C}}}$ integrating the complex linear extension of $\tau$.
- $\sigma (H_{{\mathbb {C}}}) = H_{{\mathbb {C}}}$ for the conjugation of $G_{{\mathbb {C}}}$ with respect to *G*.
- $\kappa _h = e^{-\frac{\pi i}{2} \mathop {\textrm{ad}}\nolimits h}$ integrates to the automorphism $\kappa _h^G(g) = \exp \big (-\frac{\pi i}{2}h\big )g \exp \big (\frac{\pi i}{2}h\big )$ of $G_{{\mathbb {C}}}$.

Note that

8.1 $$\begin{aligned} \tau _h^G:= (\kappa _h^G)^2 \end{aligned}$$

is a holomorphic involutive automorphism of $G_{{\mathbb {C}}}$ inducing $\tau _h$ on the Lie algebra ${{\mathfrak {g}}}$.

Let

$$\begin{aligned} \Xi := G.\mathop {\textrm{Exp}}\nolimits _{eK}(i\Omega _{{\mathfrak {p}}}) \subseteq G_{{\mathbb {C}}}/K_{{\mathbb {C}}}\end{aligned}$$

be the crown of *G*/*K*. The involution $\tau _h$ on *G* preserves *K*, hence induces an involution on *G*/*K*, and we extend it to an antiholomorphic involution $\overline{\tau }_h$ on $G_{{\mathbb {C}}}/K_{{\mathbb {C}}}$. The canonical map $G \times _K i \Omega _{{\mathfrak {p}}}\rightarrow \Xi$ is a diffeomorphism ([52, Prop. 4.7]) and

$$\begin{aligned} \overline{\tau }_h(g.\mathop {\textrm{Exp}}\nolimits (ix)) = \tau _h(g).\mathop {\textrm{Exp}}\nolimits (-i \tau _h(x)) \end{aligned}$$

implies that

8.2 $$\begin{aligned} \Xi ^{\overline{\tau }_h} = G^{\tau _h}.\mathop {\textrm{Exp}}\nolimits (i \Omega _{{\mathfrak {p}}}^{-\tau _h}) = \exp ({{\mathfrak {q}}}_{{\mathfrak {p}}}).\mathop {\textrm{Exp}}\nolimits (i \Omega _{{{\mathfrak {h}}}_{{\mathfrak {p}}}}) \ \cong G^h_e \times _{K^h_e} i\Omega _{{{\mathfrak {h}}}_{{\mathfrak {p}}}}. \end{aligned}$$

(see the proof of [52, Thm. 6.1] for details). This describes the fixed point as a “real crown domain” of the Riemannian symmetric space $(G/K)^{\tau _h} = \mathop {\textrm{Exp}}\nolimits ({{\mathfrak {q}}}_{{\mathfrak {p}}})$.

For an open subgroup $H_{{\mathbb {C}}}\subseteq G^\tau _{{\mathbb {C}}}$ (where $\tau$ denotes the holomorphic involution) with $G \cap H_{{\mathbb {C}}}= H$, we obtain an embedding $M = G/H \hookrightarrow G_{{\mathbb {C}}}/H_{{\mathbb {C}}}$. Then the stabilizer of

$$\begin{aligned} m_K:= \mathop {\textrm{Exp}}\nolimits _{eH}\Big (\frac{\pi i}{2} h\Big ) = \exp \Big (\frac{\pi i}{2} h\Big ) H_{{\mathbb {C}}}\in G_{{\mathbb {C}}}/H_{{\mathbb {C}}}\end{aligned}$$

coincides with *K*, so that $G.m_K \cong G/K$ ([52, Thm. 5.4]). Accordingly,

$$\begin{aligned} K_{{\mathbb {C}}}:= (\kappa _h^G)^{-1}(H_{{\mathbb {C}}}) \end{aligned}$$

is an open subgroup of $G_{{\mathbb {C}}}^\theta$ that coincides with the stabilizer $G_{{\mathbb {C}}}^{m_K}$. In this sense $G_{{\mathbb {C}}}/H_{{\mathbb {C}}}\cong G_{{\mathbb {C}}}/K_{{\mathbb {C}}}$, but with different base points $m_H:= eH_{{\mathbb {C}}}$ and $m_K$. Recall that $\tau = e^{\pi i \mathop {\textrm{ad}}\nolimits h}\theta = e^{\frac{\pi i}{2} \mathop {\textrm{ad}}\nolimits h}\theta e^{-\frac{\pi i}{2} \mathop {\textrm{ad}}\nolimits h}$ implies $\theta = (\kappa _h^G)^{-1} \tau \kappa _h^G$. The invariance of $H_{{\mathbb {C}}}$ under $\tau _h^G$ implies that

$$\begin{aligned} H_{{\mathbb {C}}}= (\kappa _h^G)^{-1}(K_{{\mathbb {C}}}),\end{aligned}$$

so that $K_{{\mathbb {C}}}$ and $H_{{\mathbb {C}}}$ are exchanged by the order-4 automorphism $\kappa _h^G$ and invariant under $\tau _h^G$.

As $\tau _h^G$ commutes with $\kappa _h^G$, it also leaves $K_{{\mathbb {C}}}$ invariant. Moreover, $\sigma \kappa _h^G \sigma = (\kappa _h^G)^{-1}$ entails

$$\begin{aligned} \sigma (K_{{\mathbb {C}}}) = \kappa _h^G(\sigma (H_{{\mathbb {C}}})) = \kappa ^G_h(H_{{\mathbb {C}}}) = K_{{\mathbb {C}}}.\end{aligned}$$

Therefore, the antiholomorphic extension $\overline{\tau }_h^G$ also preserves $K_{{\mathbb {C}}}$ and induces on $G_{{\mathbb {C}}}/K_{{\mathbb {C}}}\cong G_{{\mathbb {C}}}/H_{{\mathbb {C}}}$ an antiholomorphic involution $\overline{\tau }_h$ fixing the base point $m_K$ with stabilizer $K_{{\mathbb {C}}}$. Then

$$\begin{aligned} m_H':= \overline{\tau }_h(m_H) =\overline{\tau }_h\Big (\exp \Big (-\frac{\pi i}{2}h\Big ).m_K\Big ) =\exp \Big (\frac{\pi i}{2}h\Big ).m_K =\exp \Big (\pi i h\Big ).m_H,\end{aligned}$$

may be different from $m_H$.

### Remark 8.1

The condition $m_H= m_H'$ is equivalent to $\exp (\pi i h) \in H_{{\mathbb {C}}}$. Note that $e^{\pi i \mathop {\textrm{ad}}\nolimits h} = \tau _h \in \mathop {\textrm{Aut}}\nolimits ({{\mathfrak {g}}}_{{\mathbb {C}}})$ is an involution that commutes with $\tau$, so that the choice of $H_{{\mathbb {C}}}$ determines whether $\exp (\pi i h)$ is contained in $H_{{\mathbb {C}}}$ or not.

For ${{\mathfrak {g}}}= \mathop {{\mathfrak {sl} }}\nolimits _2({{\mathbb {R}}})$, $G = \mathop {\textrm{Inn}}\nolimits ({{\mathfrak {g}}})$, and $$h = \frac{1}{2}\begin{pmatrix} 1 &{}\quad 0 \\ 0 &{}\quad -1 \end{pmatrix}$$, we obtain on $\mathop {\textrm{SL}}\nolimits _2({{\mathbb {R}}})\subseteq \mathop {\textrm{SL}}\nolimits _2({{\mathbb {C}}})$ the involution

$$\begin{aligned} \tau \begin{pmatrix} a &{}\quad b \\ c &{}\quad d \end{pmatrix} = \begin{pmatrix} d &{}\quad c \\ b &{}\quad a \end{pmatrix}. \end{aligned}$$

For $g \in \mathop {\textrm{SL}}\nolimits _2({{\mathbb {C}}})$, the condition $\tau \mathop {\textrm{Ad}}\nolimits (g)\tau = \mathop {\textrm{Ad}}\nolimits (g)$ is equivalent to $\tau (g)g^{-1} \in \ker (\mathop {\textrm{Ad}}\nolimits ) = \{\pm {{\textbf {1}}}\}$. As

$$\begin{aligned} e^{\pi i \mathop {\textrm{ad}}\nolimits h} = \mathop {\textrm{Ad}}\nolimits (\exp \pi ih) = \mathop {\textrm{Ad}}\nolimits \begin{pmatrix} i &{}\quad 0 \\ 0 &{}\quad -i \end{pmatrix} \quad \text{ and } \quad \tau \begin{pmatrix} i &{}\quad 0 \\ 0 &{}\quad -i \end{pmatrix} \begin{pmatrix} i &{}\quad 0 \\ 0 &{}\quad -i \end{pmatrix}^{-1} = - {{\textbf {1}}},\end{aligned}$$

it follows that

$$\begin{aligned} \tau _h = e^{\pi i \mathop {\textrm{ad}}\nolimits h} \in G_{{\mathbb {C}}}^\tau \setminus (G_{{\mathbb {C}}}^\tau )_e.\end{aligned}$$

In particular, $K_{{\mathbb {C}}}$ and $H_{{\mathbb {C}}}$ have two connected components in $G_{{\mathbb {C}}}\cong \mathop {\textrm{PSL}}\nolimits _2({{\mathbb {C}}})$.

In $G \cong \mathop {\textrm{PSL}}\nolimits _2({{\mathbb {R}}})$, a similar argument shows that $$\theta = \mathop {\textrm{Ad}}\nolimits \begin{pmatrix} 0 &{}\quad -1 \\ 1 &{}\quad 0 \end{pmatrix} \in G^\tau {\setminus } G^\tau _e$$. So $G^\tau$ also has two connected components, but only its identity component $G^\tau _e$ acts causally on ${{\mathfrak {q}}}$. Therefore, $H = G^\tau _e$, but for $H_{{\mathbb {C}}}$ we have two choices, $G_{{\mathbb {C}}}^\tau$, or its identity component.

Comparing with the arguments in [52, Lemma 6.3], where $\alpha _{\pi i}= \tau _h$ on *M*, we have to be more careful in the present context. Here $\overline{\tau }_h$ restricts to a map

$$\begin{aligned} M = G.m_H \rightarrow M':= G.m_H' = \exp (\pi i h).M,\end{aligned}$$

and these two copies of *G*/*H* may not be identical. However, the antiholomorphic map

$$\begin{aligned} \sigma _M:= \alpha _{\pi i} \circ \overline{\tau }_h \end{aligned}$$

maps *M* to itself, fixes the base point $m_H$ and commutes with the *G*-action. Hence it fixes *M* pointwise and describes a “complex conjugation” with respect to *M*. In particular, the two maps

$$\begin{aligned} \overline{\tau }_h : M \rightarrow M' \quad \text{ and } \quad \alpha _{\pi i} : M \rightarrow M' \end{aligned}$$

coincide on *M*.

We define the *KMS wedge domain*

8.3 $$\begin{aligned} W^{\textrm{KMS}}:= \{ m \in M : \alpha _{it}(m) \in \Xi \text{ for } 0< t < \pi \}. \end{aligned}$$

### Theorem 8.2

$$W^{\textrm{KMS}} = W_M^+(h)_{eH}$$.

### Proof

“$\subseteq$”: For $z \in {{\mathbb {C}}}$ and $p \in M$, we first observe that

$$\begin{aligned} \overline{\tau }_h(\alpha _z(p)) = \alpha _{\overline{z}}(\overline{\tau }_h(p)) = \alpha _{\overline{z}}\alpha _{\pi i}(p) = \alpha _{\pi i + \overline{z}}(p).\end{aligned}$$

For $z = \frac{\pi i}{2}$, we thus obtain $\alpha _{\pi i/2}(p) \in M_{{\mathbb {C}}}^{\overline{\tau }_h}$. We conclude that

8.4 $$\begin{aligned} \alpha _{\frac{\pi i}{2}}(W^{\textrm{KMS}}) \subseteq \Xi ^{\overline{\tau }_h} = \exp ({{\mathfrak {q}}}_{{\mathfrak {p}}}).\mathop {\textrm{Exp}}\nolimits (i \Omega _{{{\mathfrak {h}}}_{{\mathfrak {p}}}}). \end{aligned}$$

Hence,

$$\begin{aligned} W^{\textrm{KMS}} \subseteq \kappa _h\big (\Xi ^{\overline{\tau }_h}\big ) = G^h_e.\mathop {\textrm{Exp}}\nolimits _{eH}(\kappa _h(i \Omega _{{{\mathfrak {h}}}_{{\mathfrak {p}}}})),\end{aligned}$$

where

$$\begin{aligned} \kappa _h(i\Omega _{{{\mathfrak {h}}}_{{\mathfrak {p}}}}) = \Big \{ x \in {{\mathfrak {q}}}_{{\mathfrak {k}}}: \Vert \mathop {\textrm{ad}}\nolimits x\Vert < \frac{\pi }{2}\Big \} =: \Omega _{{{\mathfrak {q}}}_{{\mathfrak {k}}}}.\end{aligned}$$

This suggest to define a “polar wedge domain” as

$$\begin{aligned} W_M^{\textrm{pol}}(h):= G^h_e.\mathop {\textrm{Exp}}\nolimits (\Omega _{{{\mathfrak {q}}}_{{\mathfrak {k}}}})\subseteq M.\end{aligned}$$

We actually know from Theorem 3.6 that this is the connected component $W = W_M^+(h)_{eH} \subseteq W_M^+(h)$ containing the base point. We thus obtain

8.5 $$\begin{aligned} W^{\textrm{KMS}} \subseteq W = W_M^+(h)_{eH}. \end{aligned}$$

“$\supseteq$”: To see that $W_M^+(h)_{eH}\subseteq W^{\textrm{KMS}}$, we first recall from the first part of the proof that

$$\begin{aligned} W_M^+(h)_{eH} = \kappa _h\big (\Xi ^{\overline{\tau }_h}\big ) = G^h_e.\mathop {\textrm{Exp}}\nolimits _{eH}(\kappa _h(i \Omega _{{{\mathfrak {h}}}_{{\mathfrak {p}}}})) = G^h_e.\alpha _{-\pi i/2}(\mathop {\textrm{Exp}}\nolimits _{eK}(i\Omega _{{{\mathfrak {h}}}_{{\mathfrak {p}}}})).\end{aligned}$$

To see that this domain is contained in the $G^h_e$-invariant domain $W^{\textrm{KMS}}\subseteq M$, we thus have to show that, for $x \in \Omega _{{{\mathfrak {h}}}_{{\mathfrak {p}}}}$, we have

$$\begin{aligned} \alpha _{it}.\mathop {\textrm{Exp}}\nolimits _{eK}(ix) \in \Xi \quad \text{ for } \quad |t| < \pi /2.\end{aligned}$$

Let ${{\mathfrak {t}}}_{{\mathfrak {q}}}\subseteq {{\mathfrak {q}}}_{{\mathfrak {k}}}$ is a maximal abelian subspace (they are all conjugate under $(H_K)_e$). Then, ${{\mathfrak {a}}}_{{\mathfrak {h}}}:= i\kappa _h({{\mathfrak {t}}}_{{\mathfrak {q}}}) \subseteq {{\mathfrak {h}}}_{{\mathfrak {p}}}$ is also maximal abelian and $\Omega _{{{\mathfrak {h}}}_{{\mathfrak {p}}}} = e^{\mathop {\textrm{ad}}\nolimits {{\mathfrak {h}}}_{{\mathfrak {k}}}}.\Omega _{{{\mathfrak {a}}}_{{\mathfrak {h}}}}$. So it suffices to show that, for $x \in \Omega _{{{\mathfrak {a}}}_{{\mathfrak {h}}}}$ and $|t| < \pi /2$, we have $\alpha _{it}.\mathop {\textrm{Exp}}\nolimits _{eK}(ix) \in \Xi .$ By Proposition 2.8, ${{\mathfrak {t}}}_{{\mathfrak {q}}}$ is contained in a $\tau$-invariant subalgebra ${{\mathfrak {s}}}\cong \mathop {{\mathfrak {sl} }}\nolimits _2({{\mathbb {R}}})^s$, where ${{\mathbb {R}}}h + {{\mathfrak {s}}}$ is generated by *h* and ${{\mathfrak {t}}}_{{\mathfrak {q}}}$ and $h = h_0 + h_1 + \cdots + h_s$, where $h_j$, $j = 1,\ldots , s$, is an Euler element in a simple ideal ${{\mathfrak {s}}}_j \cong \mathop {{\mathfrak {sl} }}\nolimits _2({{\mathbb {R}}})$ of ${{\mathfrak {s}}}$. Then ${{\mathfrak {a}}}_{{\mathfrak {h}}}= i \kappa _h({{\mathfrak {t}}}_{{\mathfrak {q}}}) \subseteq {{\mathfrak {a}}}$ is spanned by *s* Euler elements $x_1, \ldots , x_s$ and

$$\begin{aligned} \Omega _{{{\mathfrak {a}}}_{{\mathfrak {h}}}} = \Big \{ \sum _{j = 1}^s t_j x_j : (\forall j)\ |t_j| < \pi /2\Big \}.\end{aligned}$$

Let $S:= \langle \exp {{\mathfrak {s}}}\rangle$ and $\Xi _S:= S.\mathop {\textrm{Exp}}\nolimits (i(\Omega _{{\mathfrak {p}}}\cap {{\mathfrak {s}}})) \subseteq \Xi$. Then the discussion in Remark D.1 implies that, for $|t| < \pi /2$ and $x = \sum _j t_j x_j \in \Omega _{{{\mathfrak {a}}}_{{\mathfrak {h}}}}$, we have $\alpha _{it}(\mathop {\textrm{Exp}}\nolimits _{eK}(ix)) \in \Xi _S \subseteq \Xi .$ $\square$

The preceding proof implies in particular the following interesting observation:

### Corollary 5.8

For every $m \in \Xi ^{\overline{\tau }_h}$, we have $\alpha _{it}(m) \in \Xi$ for $|t| < \pi /2$, so that the orbit map $\alpha ^m$ extends to a holomorphic map ${\mathcal {S}}_{\pm \pi /2} \rightarrow \Xi$.

### Corollary 6.3

$\alpha _{\frac{\pi i}{2}} : W^{\textrm{KMS}} \rightarrow \Xi ^{\overline{\tau }_h}$ is a diffeomorphism that induces an equivalence of fiber bundles

$$\begin{aligned} W^{\textrm{KMS}} \cong G^h_e \times _{K^h_e} \Omega _{{{\mathfrak {q}}}_{{\mathfrak {k}}}} \rightarrow G^h_e \times _{K^h_e} i\Omega _{{{\mathfrak {h}}}_{{\mathfrak {p}}}} \cong \Xi ^{\overline{\tau }_h}.\end{aligned}$$

### Proof

Theorem 8.2 implies in particular that $\alpha _{\frac{\pi i}{2}} : W^{\textrm{KMS}} \rightarrow \Xi ^{\overline{\tau }_h}$ is bijective. Since $W^{\textrm{KMS}} = W_M^+(h)_{eH}$ is an open subset of *M* and $\Xi ^{\overline{\tau }_h}$ an open subset of $M_{{\mathbb {C}}}^{\overline{\tau }_h}$, it actually is a diffeomorphism. The second assertion follows from the fact that it commutes with the action of the subgroup $G^h_e$. $\square$

## Figures and Tables

**Table 1:**
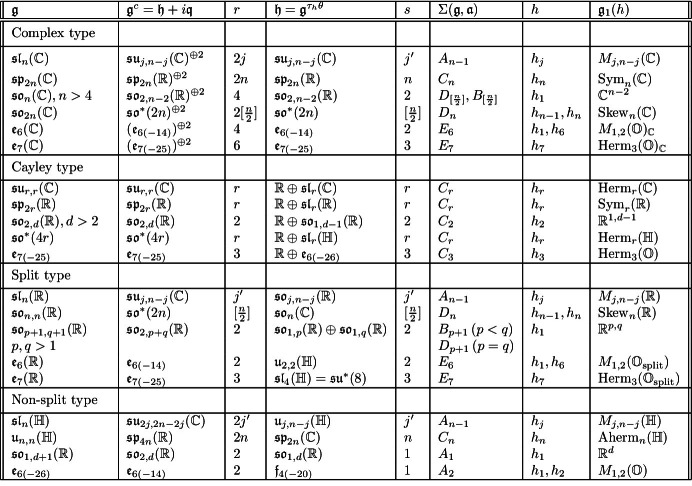
Irreducible ncc symmetric Lie algebras with corresponding causal Euler elements $h \in {{\mathfrak {a}}}$

## Irreducible ncc symmetric Lie algebras

The following table lists all irreducible non-compactly causal symmetric Lie algebras $({{\mathfrak {g}}},\tau )$ according to the following types:

- Complex type: ${{\mathfrak {g}}}= {{\mathfrak {h}}}_{{\mathbb {C}}}$ and $\tau$ is complex conjugation with respect to ${{\mathfrak {h}}}$. In this case ${{\mathfrak {g}}}^c \cong {{\mathfrak {h}}}^{\oplus 2}$, so that $\mathop {\textrm{rk}}\nolimits _{{\mathbb {R}}}({{\mathfrak {g}}}^c) = 2 \mathop {\textrm{rk}}\nolimits _{{\mathbb {R}}}({{\mathfrak {h}}})$.
- Cayley type (CT): $\tau = \tau _{h_1}$ for an Euler element $h_1 \in {{\mathfrak {h}}}$. Then $\mathop {\textrm{rk}}\nolimits _{{\mathbb {R}}}({{\mathfrak {g}}}^c) = \mathop {\textrm{rk}}\nolimits _{{\mathbb {R}}}({{\mathfrak {g}}}) = \mathop {\textrm{rk}}\nolimits _{{\mathbb {R}}}({{\mathfrak {h}}})$.
- Split type (ST): $\tau \not =\tau _{h_1}$ for all $h_1 \in {{\mathfrak {h}}}\cap {\mathcal {E}}({{\mathfrak {g}}})$ and $\mathop {\textrm{rk}}\nolimits _{{\mathbb {R}}}{{\mathfrak {h}}}= \mathop {\textrm{rk}}\nolimits _{{\mathbb {R}}}{{\mathfrak {g}}}^c$:
- Non-split type (NST): $\tau \not =\tau _{h_1}$ for all $h_1 \in {{\mathfrak {h}}}\cap {\mathcal {E}}({{\mathfrak {g}}})$ and $\mathop {\textrm{rk}}\nolimits _{{\mathbb {R}}}{{\mathfrak {h}}}=\frac{ \mathop {\textrm{rk}}\nolimits _{{\mathbb {R}}}{{\mathfrak {g}}}^c}{2}$:

In Table 1, we write $r = \mathop {\textrm{rk}}\nolimits _{{\mathbb {R}}}({{\mathfrak {g}}}^c)$ and $s = \mathop {\textrm{rk}}\nolimits _{{\mathbb {R}}}({{\mathfrak {h}}})$. Further ${{\mathfrak {a}}}\subseteq {{\mathfrak {p}}}$ is maximal abelian of dimension *r*. For root systems $\Sigma ({{\mathfrak {g}}},{{\mathfrak {a}}})$ of type $A_{n-1}$, there are $n-1$ Euler elements $h_1, \ldots , h_{n-1}$, but for the other root systems there are less; see [38, Thm. 3.10] for the concrete list. For $1 \le j <n$ we write $j':= \min (j,n-j)$.

## Geodesics in symmetric spaces

This appendix contains some elementary observations concerning geodesics in symmetric spaces.

### Lemma B.1

Let $M = G/H$ be a symmetric space with symmetric Lie algebra $({{\mathfrak {g}}},\tau )$, $x \in {{\mathfrak {g}}}$ and $y \in {{\mathfrak {q}}}$. Then

B.1 $$\begin{aligned} \exp (tx) H = \exp (ty) H \quad \text{ for } \text{ all } \quad t \in {{\mathbb {R}}}\end{aligned}$$

holds if and only if $p_{{\mathfrak {q}}}(x) = y$ and $[x,y] = 0$.

In particular, $\gamma (t):= \exp (tx)H$ is a geodesic in *M* if and only if $[x, \tau (x)] = 0$.

### Proof

The relation (B.1) is equivalent to

$$\begin{aligned} \exp (-ty) \exp (tx) \in H \subseteq G^{\tau ^G} \quad \text{ for } \text{ all } \quad t \in {{\mathbb {R}}}.\end{aligned}$$

Applying $\tau ^G$, we obtain

$$\begin{aligned} \exp (ty) \exp (t \tau (x)) = \exp (-ty) \exp (tx), \end{aligned}$$

which leads to $\exp (2ty) = \exp (tx) \exp (-t \tau (x)).$ Evaluating the derivative of this curve in the right trivialization of *T*(*G*), we get

$$\begin{aligned} 2y = x + e^{t\ ad x}(-\tau (x))= x - e^{t\ ad x}(\tau (x))\quad \text{ for } \text{ all } \quad t \in {{\mathbb {R}}}. \end{aligned}$$

For $t = 0$ we get $p_{{\mathfrak {q}}}(x) = y$, and taking derivatives in 0 shows that $[x,\tau (x)] = 0.$

If, conversely, this condition is satisfied, then $x = x_{{\mathfrak {h}}}+ x_{{\mathfrak {q}}}$ with $x_{{\mathfrak {h}}}\in {{\mathfrak {h}}}$ and $x_{{\mathfrak {q}}}\in {{\mathfrak {q}}}$, where

$$\begin{aligned} 0 = [x,\tau (x)] = 2 [x_{{\mathfrak {h}}}, x_{{\mathfrak {q}}}]. \end{aligned}$$

Therefore,

$$\begin{aligned} \exp (tx)H = \exp (t x_{{\mathfrak {q}}}) \exp (t x_{{\mathfrak {h}}}) H = \exp (t x_{{\mathfrak {q}}}) H = \mathop {\textrm{Exp}}\nolimits _{eH}(t x_{{\mathfrak {q}}}) \end{aligned}$$

is a geodesic in *M*. $\square$

The following lemma provides important information on the subset $M^x$.

### Lemma B.2

Let $x \in {{\mathfrak {g}}}$ and write

$$\begin{aligned} M^x:= \{ gH \in M : \mathop {\textrm{Ad}}\nolimits (g)^{-1}x \in {{\mathfrak {q}}}\}. \end{aligned}$$

Then $M^x$ is a submanifold of *M* which is invariant under the action of $G^x$, and the orbits or $G^x_e$ are the connected components of $M^x$.

### Proof

Let $m_0 = g_0 H \in M^x$ and $x_c:= \mathop {\textrm{Ad}}\nolimits (g_0)^{-1} x$. For $y \in {{\mathfrak {q}}}$ we have

$$\begin{aligned} \mathop {\textrm{Exp}}\nolimits _{m_0}(g_0.y) = g_0.\mathop {\textrm{Exp}}\nolimits _{eH}(y) = g_0 (\exp y) H = \exp (\mathop {\textrm{Ad}}\nolimits (g_0)y).m_0 \end{aligned}$$

and

$$\begin{aligned} \mathop {\textrm{Ad}}\nolimits (g_0\exp (y))^{-1} x= & {} e^{-\mathop {\textrm{ad}}\nolimits y} x_c = \cosh (\mathop {\textrm{ad}}\nolimits y)x_c - \sinh (\mathop {\textrm{ad}}\nolimits y) x_c\\= & {} \underbrace{\cosh (\mathop {\textrm{ad}}\nolimits y)x_c}_{\in {{\mathfrak {q}}}} - \underbrace{\frac{\sinh (\mathop {\textrm{ad}}\nolimits y)}{\mathop {\textrm{ad}}\nolimits y} [y,x_c]}_{\in {{\mathfrak {h}}}}. \end{aligned}$$

Let $U \subseteq {{\mathfrak {q}}}$ be a 0-neighborhood for which $\mathop {\textrm{Exp}}\nolimits _{eH}\vert _U$ is a diffeomorphism onto an open subset of *M* and the spectral radius of $\mathop {\textrm{ad}}\nolimits y$ is smaller than $\pi$ for $y \in U$. Then $\frac{\sinh (\mathop {\textrm{ad}}\nolimits y)}{\mathop {\textrm{ad}}\nolimits y} : {{\mathfrak {h}}}\rightarrow {{\mathfrak {h}}}$ is invertible. With the above formula, we thus conclude for $y \in U$ that $\mathop {\textrm{Exp}}\nolimits _{m_0}(g_0.y) \in M^x$ is equivalent to $[y,x_c] = 0$, which is equivalent to $\mathop {\textrm{Ad}}\nolimits (g_0)y \in {{\mathfrak {g}}}^x$. This shows that $M^x$ is a submanifold of *M*.

As $\mathop {\textrm{Exp}}\nolimits _{m_0}(g_0.y) = \exp (\mathop {\textrm{Ad}}\nolimits (g_0)y).m_0 \in \exp ({{\mathfrak {g}}}^x).m_0$, it further follows that the orbit of $m_0$ under the connected group $G^x_e$ contains a neighborhood of $m_0$. This shows that the orbits of $G^x_e$ in $M^x$ are connected open subsets, hence coinciding with its connected components. $\square$

### Remark B.3

For $x \in {{\mathfrak {q}}}$ the centralizer ${{\mathfrak {g}}}^x$ is $\tau$-invariant, so that ${{\mathfrak {g}}}^x = {{\mathfrak {h}}}^x \oplus {{\mathfrak {q}}}^x$ and the dimension of the $G^x_e$-orbit through *eH* is $\mathop {\textrm{dim}}\nolimits {{\mathfrak {q}}}^x$. We have

$$\begin{aligned} M^x_{eH} \cong G^x_e/ (H \cap G^x_e), \end{aligned}$$

and Lemma B.2 shows that the geodesic $\mathop {\textrm{Exp}}\nolimits _{eH}({{\mathbb {R}}}x)$ is central in the symmetric space $M^x_{eH}$ in the sense that its tangent space ${{\mathbb {R}}}x$ is central in the Lie algebra ${{\mathfrak {g}}}^x$ (cf. [36]).

### Lemma B.4

For $y \in {{\mathfrak {q}}}$, the equality $M^y = G^y.eH$ is equivalent to

B.2 $$\begin{aligned} {\mathcal {O}}_y \cap {{\mathfrak {q}}}= \mathop {\textrm{Ad}}\nolimits (H) y. \end{aligned}$$

### Proof

As $y \in {{\mathfrak {q}}}$, the base point *eH* is contained in $M^y$, and thus $G^y.eH \subseteq M^y$. So the equality $M^y = G^y.eH$ means that $M^y \subseteq G^y.eH$, i.e., $\mathop {\textrm{Ad}}\nolimits (g)^{-1}y \in {{\mathfrak {q}}}$ implies $gH \in G^y.eH$, resp., $g \in G^y H$. This in turn is equivalent to $\mathop {\textrm{Ad}}\nolimits (g)^{-1}y \in \mathop {\textrm{Ad}}\nolimits (H)y$. $\square$

## Lawson’s Theorem

Let $(G,\tau ^G,H,C)$ be a causal symmetric Lie group, i.e., $\tau ^G$ is an involutive automorphism of *G*, $H \subseteq G^{\tau ^G}$ an open subgroup and $C \subseteq {{\mathfrak {q}}}$ a hyperbolic pointed generating $\mathop {\textrm{Ad}}\nolimits (H)$-invariant closed convex cone. We write ${{\mathfrak {g}}}= {{\mathfrak {h}}}\oplus {{\mathfrak {q}}}$ for the corresponding decomposition of ${{\mathfrak {g}}}= \mathop {{\textbf {L}}}\nolimits (G)$.

According to [30, Lemma 2.3], $\exp \vert _{C}$ is injective if and only if $\Gamma _Z:= {{\mathfrak {z}}}({{\mathfrak {g}}}) \cap \exp _G^{-1}(e)$ satisfies

$$\begin{aligned} \Gamma _Z \cap {{\mathfrak {q}}}= \Gamma _Z \cap (C-C) = \{0\}. \end{aligned}$$

If ${{\mathfrak {z}}}({{\mathfrak {g}}}) \subseteq {{\mathfrak {q}}}$, this condition is satisfied if and only if $\exp \vert _{{{\mathfrak {z}}}({{\mathfrak {g}}})}$ is injective. This condition is always satisfied if ${{\mathfrak {g}}}$ is semisimple because ${{\mathfrak {z}}}({{\mathfrak {g}}}) = \{0\}$ in this case.

Suppose that $\Gamma _Z = \{0\}$. By [30, Lemma 2.4], $\exp \vert _C$ is a homeomorphism onto a closed subset of *G* if and only if, for no non-zero $x \in C \cap {{\mathfrak {z}}}({{\mathfrak {g}}})$, the subgroup $\overline{\exp ({{\mathbb {R}}}x)}$ is compact. By [30, Thm. 3.1], this in turn is equivalent to the polar map

$$\begin{aligned} \Phi : C \times G^{\tau ^G} \rightarrow \exp (C) G^{\tau ^G} \end{aligned}$$

being a homeomorphism onto a closed subset of *G*. [30, Thm. 3.1] further shows that $\exp (C) G^{\tau ^G}$ is a subsemigroup of *G*. If *G* is 1-connected, then the subgroup $G^{\tau ^G}$ is connected and *Z*(*G*) is simply connected, so that all requirements from above are satisfied ([30, Cor. 3.2]).

### Theorem C.1

(Lawson’s Theorem) Let $(G, \tau ^G, H, C)$ be a non-compactly causal reductive symmetric Lie group. Suppose that ${{\mathfrak {z}}}({{\mathfrak {g}}}) \subseteq {{\mathfrak {q}}}$ and that $\Gamma _Z = \{0\}$. Then $S:= \exp (C)H$ is a closed subsemigroup of *G* with Lie wedge $\mathop {{\textbf {L}}}\nolimits (S) = {{\mathfrak {h}}}+ C$.

### Proof

Our assumption implies that $\exp \vert _{{{\mathfrak {z}}}({{\mathfrak {g}}})} : {{\mathfrak {z}}}({{\mathfrak {g}}}) \rightarrow Z(G)_e$ is bijective, hence a diffeomorphism onto the closed subgroup $Z(G)_e$. It follows in particular that $\exp ({{\mathbb {R}}}x) \cong {{\mathbb {R}}}$ is non-compact for each non-zero $x \in {{\mathfrak {z}}}({{\mathfrak {g}}})$. Therefore the polar map $\Phi$ is a homeomorphism onto a closed subset and the remaining assertions follow from [30, Thm. 3.1]. $\square$

### Remark C.2

(a) If *G* is reductive, then $G = (G,G)_e Z(G)_e$ and if $x \in {{\mathfrak {z}}}({{\mathfrak {g}}})$ satisfies $\exp z \in (G,G)_e$, then $\exp z \in Z((G,G)_e)^{-\tau ^G}$, which is a discrete group. We shall see below that this group may be infinite, even if $\Gamma _Z = \{0\}$.

(b) If $M = G_{{\mathbb {C}}}/G$ is of complex type and *G* is hermitian, then $Z(G_{{\mathbb {C}}})$ is finite.

(c) If *M* is of non-complex type and irreducible, then ${{\mathfrak {g}}}^c$ is simple hermitian with ${{\mathfrak {z}}}({{\mathfrak {k}}}^c) = {{\mathbb {R}}}i h$, where $h \in {\mathcal {E}}({{\mathfrak {g}}})$ is a causal Euler element. If *Z*(*G*) is infinite, then ${{\mathfrak {g}}}$ is also hermitian, hence of tube type because it contains an Euler element [38]. Then all Euler elements in ${{\mathfrak {g}}}$ are conjugate and this implies that $({{\mathfrak {g}}},\tau )$ is of Cayley type. So ${{\mathfrak {z}}}({{\mathfrak {k}}}) \subseteq {{\mathfrak {q}}}_{{\mathfrak {k}}}$ and thus $Z(G)^{-\tau }$ is infinite if *G* is simply connected. This shows that it is possible that $(G,G)_e \cap Z(G)_e$ is infinite.

A concrete example is the group

$$\begin{aligned} G:= ({\widetilde{\mathop {\textrm{SL}}\nolimits }}_2({{\mathbb {R}}}) \times {{\mathbb {R}}})/D, \end{aligned}$$

where $D \subseteq Z({\widetilde{\mathop {\textrm{SL}}\nolimits }}_2({{\mathbb {R}}})) \times {{\mathbb {R}}}\cong {{\mathbb {Z}}}\times {{\mathbb {R}}}$ is the graph of a non-zero homomorphism $\gamma : Z({\widetilde{\mathop {\textrm{SL}}\nolimits }}_2({{\mathbb {R}}})) \rightarrow {{\mathbb {R}}}$. Then $Z(G) \cong {{\mathbb {R}}}$ and $Z(G) \cap (G,G) \cong Z({\widetilde{\mathop {\textrm{SL}}\nolimits }}_2({{\mathbb {R}}})) \cong {{\mathbb {Z}}}$.

### Remark C.3

Suppose that $h_0 \in {{\mathfrak {h}}}\cap {\mathcal {E}}({{\mathfrak {g}}})$ is such that $-\tau _{h_0}(C) = C$, then $C \cap {{\mathfrak {z}}}({{\mathfrak {g}}})$ is contained in $C \cap -C = \{0\}$. Therefore the condition on $C \cap {{\mathfrak {z}}}({{\mathfrak {g}}})$ in Lawson’s Theorem (cf. Appendix C) is satisfied.

## de Sitter space

In this appendix we collect some concrete observations concerning de Sitter space $\mathop {\textrm{dS}}\nolimits ^d$, which is an important example of a non-compactly causal symmetric space. Some facts on 2-dimensional de Sitter space are used in particular in some of our proofs to verify the corresponding assertions for ${{\mathfrak {g}}}= \mathop {{\mathfrak {sl} }}\nolimits _2({{\mathbb {R}}})$.

In $(d+1)$-dimensional Minkowski space ${{\mathbb {R}}}^{1,d}$, we write the Lorentzian form as

$$\begin{aligned} \beta (x,y) = x_0y_0 - {{\textbf {x}}}{{\textbf {y}}}\quad \text{ for } \quad x = (x_0,{{\textbf {x}}}), y = (y_0, {{\textbf {y}}}).\end{aligned}$$

We consider *d*-dimensional de Sitter space

$$\begin{aligned} M:= \mathop {\textrm{dS}}\nolimits ^d:= \{ x = (x_0, {{\textbf {x}}}) \in {{\mathbb {R}}}^{1,d} : x_0^2 - {{\textbf {x}}}^2 = -1\},\end{aligned}$$

$G = \mathop {\textrm{SO}}\nolimits _{1,d}({{\mathbb {R}}})_e$ and the Euler element $h \in \mathop {{\mathfrak {so} }}\nolimits _{1,d}({{\mathbb {R}}})$, defined by

$$\begin{aligned} h.(x_0, \ldots , x_d) = (x_1, x_0, 0,\ldots , 0).\end{aligned}$$

It generates the Lorentz boost in the $x_0$-$x_1$-plane. The fixed point set of the modular flow in $M = \mathop {\textrm{dS}}\nolimits ^d$ is

$$\begin{aligned} M^\alpha = M \cap \mathop {\textrm{span}}\nolimits \{{{\textbf {e}}}_2,\ldots , {{\textbf {e}}}_d\} = \{(0,0,x_2,\ldots , x_d) : x_2^2 + \cdots + x_d^2 = 1\} \cong {{\mathbb {S}}}^{d-2}.\end{aligned}$$

This submanifold is connected for $d > 2$ and consists of two points for $d = 2$. The corresponding wedge domain is the connected subset

$$\begin{aligned} W_M^+(h) = M \cap W_R = \{ x \in \mathop {\textrm{dS}}\nolimits ^d : x_1 > |x_0|\}.\end{aligned}$$

By [52, Prop. D.3], the timelike geodesics of *M* of velocity 1 take the form

$$\begin{aligned} \gamma (t) = \mathop {\textrm{Exp}}\nolimits _x(tv)= \cosh (t) x + \sinh (t) v, \qquad \beta (v,v) = 1, \beta (x,v) = 0, \beta (x,x) = 1 \end{aligned}$$

whereas the trajectories of the modular flow are

$$\begin{aligned} \alpha _t(x) = e^{th}x = (\cosh (t) x_0 + \sinh (t) x_1, \cosh (t) x_1 + \sinh (t) x_0, x_2, \ldots , x_d).\end{aligned}$$

Comparing both expressions leads for *h*-modular geodesics to the conditions

$$\begin{aligned} x_2 = \cdots = x_d = 0 \quad \text{ and } \quad v = h.x = (x_1, x_0,0,\ldots , 0).\end{aligned}$$

Therefore exactly two orbits of the modular flow are timelike geodesics. If we also ask for the geodesic to be positive with respect to the causal structure, then $x_1 > 0$ determines the geodesic uniquely.

We infer from [52, Prop. D.3] that

$$\begin{aligned} \mathop {\textrm{Exp}}\nolimits _{{{\textbf {e}}}_2}(t {{\textbf {e}}}_1) = \cos (t) {{\textbf {e}}}_2 + \sin (t) {{\textbf {e}}}_1 \end{aligned}$$

is a closed space-like geodesics. For $0< t < \pi$, its values are contained in $W_M^+(h)$, and this geodesic arc connects the two fixed points ${{\textbf {e}}}_2$ to $-{{\textbf {e}}}_2$ of the modular flow.

### Remark D.1

In addition to *h*, we also consider the Euler elements defined by

$$\begin{aligned} h_d(x_0,\ldots , x_d) = (x_d,0,\ldots , 0, x_0).\end{aligned}$$

The involution corresponding to *h* acts on ${{\mathbb {R}}}^{1+d}$ by

$$\begin{aligned} \tau _h(x_0, x_1, \ldots , x_d) = (-x_0, -x_1,x_2, \ldots , x_d),\end{aligned}$$

and its antilinear extension acts on ${{\mathbb {C}}}^{1+d}$ by

$$\begin{aligned} \overline{\tau }_h(z_0, z_1, \ldots , z_d) = (-\overline{z_0}, -\overline{z_1},\overline{z_2}, \ldots , \overline{z_d}).\end{aligned}$$

Note that, in ${{\mathfrak {g}}}$, we have $\tau _h(h) = h$ and $\tau _h(h_d) = - h_d$, so that $h \in {{\mathfrak {q}}}_{{\mathfrak {p}}}$ and $h_d \in {{\mathfrak {h}}}_{{\mathfrak {p}}}$.

In ${{\mathbb {C}}}^{1+d}$, we consider the domain

$$\begin{aligned} \Xi := \{ z = x + i y \in {{\mathbb {C}}}^{1+d} : y_0> 0, y_0^2 > y_1^2 + \cdots + y_d^2 \}.\end{aligned}$$

On $\Xi$ the antiholomorphic involution $\overline{\tau }_h$ has the fixed point set

$$\begin{aligned} \Xi ^{\overline{\tau }_h}&= \Xi \cap (i{{\mathbb {R}}}^2 \oplus {{\mathbb {R}}}^{d-1}) \\&= \{ (i x_0, i x_1, x_2, \ldots , x_d) : x_0> |x_1|, - x_0^2 + x_1^2 - x_2^2 - \ldots - x_d^2= -1\}\\&= \{ (i x_0, i x_1, x_2,\ldots , x_d) : x_0> |x_1|, \underbrace{x_0^2 -x_1^2}_{>0} + x_2^2 + \cdots + x_d^2= 1\}. \end{aligned}$$

It follows in particular that

$$\begin{aligned} x_0^2 - x_1^2 \in (0,1].\end{aligned}$$

The analytic extension of the modular flow $(\alpha _t)_{t \in {{\mathbb {R}}}}$ acts on $\Xi$ by

$$\begin{aligned} \alpha _{it}(z_0, \ldots , z_d) = (\cos t \cdot z_0 + i \sin t \cdot z_1, i \sin t \cdot z_0 + \cos t \cdot z_1, z_2, \ldots , z_d).\end{aligned}$$

Starting with a $\overline{\tau }_h$-fixed element $z = (ix_0, ix_1, x_2, \ldots , x_d)$ in $\Xi$, this leads to

$$\begin{aligned} \alpha _{it}(ix_0, ix_1, x_2,\ldots , x_d) = (\cos t \cdot ix_0 - \sin t \cdot x_1, - \sin t \cdot x_0 + \cos t \cdot i x_1, x_2, \ldots , x_d)\end{aligned}$$

with imaginary part

$$\begin{aligned} (x_0 \cos t, x_1 \cos t, 0,\ldots , 0), \end{aligned}$$

so that we obtain for $|t| < \pi /2$ that

$$\begin{aligned} |x_0 \cos t| = x_0 \cos t > |x_1| \cos t, \end{aligned}$$

which implies that

D.1 $$\begin{aligned} \alpha _{it}(z) \in \Xi \quad \text{ for } \quad z \in \Xi ^{\overline{\tau }_h} \quad \text{ and } \quad |t| < \pi /2. \end{aligned}$$

### Example D.2

For the special case $d = 2$, we have $\mathop {{\mathfrak {sl} }}\nolimits _2({{\mathbb {R}}}) \cong {{\mathbb {R}}}^{1,2}$ and the Euler element

$$\begin{aligned} h^0= \frac{1}{2} \begin{pmatrix} 1 &{}\quad 0 \\ 0 &{}\quad -1 \end{pmatrix} \in \mathop {{\mathfrak {sl} }}\nolimits _2({{\mathbb {R}}})\cong {{\mathbb {R}}}^{1,2} \end{aligned}$$

corresponds to the base point ${{\textbf {e}}}_2$ (see [52]), so that

$$\begin{aligned} \mathop {{\mathfrak {sl} }}\nolimits _2({{\mathbb {R}}}) \supseteq {\mathcal {O}}_h \cong \mathop {\textrm{dS}}\nolimits ^2 \subseteq {{\mathbb {R}}}^{1,2}.\end{aligned}$$

Accordingly,

$$\begin{aligned} C= & {} \mathop {\textrm{cone}}\nolimits (e^0, f^0), \quad C^c = \mathop {\textrm{cone}}\nolimits (e^0, -f^0), \quad \quad \text{ and } \quad x_0:= \frac{1}{2}(e^0 - f^0) \\= & {} \frac{1}{2}\begin{pmatrix} 0 &{}\quad 1 \\ -1 &{}\quad 0 \end{pmatrix} \in C^c. \end{aligned}$$

For $g_t:= \exp (t x_0)$ we then have

$$\begin{aligned} \mathop {\textrm{Ad}}\nolimits (g_{\pi /2}) h^0 = -h^1, \quad \mathop {\textrm{Ad}}\nolimits (g_{\pi /2}) h^1 = h^0 \quad \text{ and } \quad \mathop {\textrm{Ad}}\nolimits (g_\pi ) h^0 = -h^0. \end{aligned}$$

We also note that, for $0< t < \pi$, the Lie algebra element $\mathop {\textrm{Ad}}\nolimits (g_t) h^0$ corresponds to $\mathop {\textrm{Exp}}\nolimits _{{{\textbf {e}}}_2}(t {{\textbf {e}}}_1) \in W_M^+(h^0)$. Note that

$$\begin{aligned} g_\pi \in K^{\tau _h^G} = K^{\tau ^G}. \end{aligned}$$

## Footnotes

1 A closed real subspace $\textsf{H}$ of a complex Hilbert space ${\mathcal {H}}$ is called standard if $\overline{\textsf{H}+i\textsf{H}}={\mathcal {H}}$ and $\textsf{H}\cap i\textsf{H}=\{0\}$

2 Note that the cones $V_+(m)$ are open, whereas *C* is closed.

3 This theorem is stated for complex hermitian Jordan triple systems, but $V = {{\mathfrak {g}}}_1(h)$ is a real form of the complex JTS $V_{{\mathbb {C}}}= {{\mathfrak {g}}}_{{{\mathbb {C}}},1}(h)$ on which we have an antilinear isomorphism $\sigma$ with $V = V_{{\mathbb {C}}}^\sigma$. Therefore, the uniqueness in the spectral decomposition shows that, for $x \in V$, the corresponding spectral tripotents are contained in *V*.

4 As ${\mathcal {D}}_+$ is invariant under the group $(H_K)_e$ which acts linearly, and this group acts transitively on the set of all maximal flat subtriples of *V* ([59, Lemma VI.3.1]), it suffices to shows that an element with a spectral resolution $x = \sum _{j = 1}^r x_j c_j$ is contained in ${\mathcal {D}}_{{\mathfrak {g}}}$ if and only if $|x_j| < 1$ for every *j*. This follows easily from (4.5).

5 This reference deals with bounded symmetric domains in complex spaces, but ${\mathcal {D}}$ can be embedded into such a domain ${\mathcal {D}}_{{\mathbb {C}}}$ by embedding ${{\mathfrak {g}}}\hookrightarrow {{\mathfrak {g}}}_{{\mathbb {C}}}\cong {{\mathfrak {g}}}^c_{{\mathbb {C}}}$. If $C_{{{\mathfrak {g}}}^c} \subseteq {{\mathfrak {g}}}^c$ is an invariant cone with $C = {{\mathfrak {g}}}\cap i C_{{{\mathfrak {g}}}^c}$, then $({{\mathfrak {g}}},\tau , C) \hookrightarrow ({{\mathfrak {g}}}_{{\mathbb {C}}}, \tau _{{{\mathfrak {g}}}_{{\mathbb {C}}}}, i C_{{{\mathfrak {g}}}^c})$ is a causal embedding and ${\mathcal {D}}_+ = H.eP^- \subseteq G^c.eP^-_{{\mathbb {C}}}= {\mathcal {D}}_+^{{\mathbb {C}}}$ is a real form of a complex bounded symmetric domain ${\mathcal {D}}_+^{{\mathbb {C}}}$; see [56, Lem. 1.4] or [26, Lem 5.1.11] for more details.

## References

[1] Araki H (1976). Relative entropy of states of von Neumann algebras. Publ. RIMS, Kyoto Univ..

[2] Bertram W, Neeb K-H (2004). Projective completions of Jordan pairs, Part I. The generalized projective geometry of a Lie algebra. J. Algebra.

[3] Bisognano J, Wichmann EH (1976). On the duality condition for quantum fields. J. Math. Phys..

[4] Bratteli, O., Robinson, D. W.: Operator Algebras and Quantum Statistical Mechanics I, 2nd ed., Texts and Monographs in Physics, Springer (1987)

[5] Bratteli, O., Robinson, D. W.: Operator Algebras and Quantum Statistical Mechanics II, 2nd ed., Texts and Monographs in Physics, Springer (1996)

[6] Bros J, Moschella U (1996). Two-point functions and quantum fields in de Sitter universe. Rev. Math. Phys..

[7] Brunetti R, Guido D, Longo R (2002). Modular localization and Wigner particles. Rev. Math. Phys..

[8] Ceyhan F, Faulkner T (2020). Recovering the QNEC from the ANEC. Commun. Math. Phys..

[9] Ciolli, F., Longo, R., Ranallo, A., Ruzzi, G.: Relative entropy and curved spacetimes, J. Geom. and Phys. **172** (2022), Paper No. 104416, 16 pp

[10] Ciolli F, Longo R, Ruzzi G (2020). The information in a wave. Commun. Math. Phys..

[11] Correa da Silva R, Lechner G (2023). Modular structure and inclusions of twisted Araki-Woods algebras. Commun. Math. Phys..

[12] Dappiaggi C, Lechner G, Morfa-Morales E (2011). Deformations of quantum field theories on spacetimes with Killing vector fields. Commun. Math. Phys..

[13] Dybalski W, Morinelli V (2020). Bisognano-Wichmann property for asymptotically complete massless QFT. Commun. Math. Phys..

[14] Fewster, C.: Lectures on quantum energy inequalities. In: Lobo, Francisco S. N. (ed.), Wormholes, Warp Drives and Energy Conditions. Springer, Fundamental Theories of Physics, Cham, **189**, 215–254, (2017). arXiv:1208.5399

[15] Fewster CJ, Hollands S (2005). Quantum energy inequalities in two-dimensional conformal field theory. Rev. Math. Phys..

[16] Faulkner, T., Leigh, R. G., Parrikar, O., Wang, H.: Modular Hamiltonians for deformed half-spaces and the averaged null energy condition, J. High Energy Phys. **2016:9** (2016), Paper 38, 35 p. arXiv:1605.08072

[17] Frahm, J., Neeb, K.-H., Ólafsson, G.: Nets of standard subspaces on non-compactly causal symmetric spaces, to appear in “Toshiyuki Kobayashi Festschrift,” Progress in Mathematics, Springer-Nature; arxiv:2303.10065

[18] Guido D, Longo R (2003). A converse Hawking-Unruh effect and dS2-CFT correspondence. Ann. Henri Poincaré.

[19] Guido D, Longo R (1995). An algebraic spin and statistics theorem. Commun. Math. Phys..

[20] Haag, R.: Local Quantum Physics. Fields, Particles, Algebras. Second edition, Texts and Monographs in Physics, Springer, Berlin (1996)

[21] Haag R, Hugenholtz NM, Winnink M (1967). On the equilibrium states in quantum statistical mechanics. Commun. Math. Phys..

[22] Helgason S (1978). Differential Geometry, Lie Groups, and Symmetric Spaces.

[23] Hilgert, J., Neeb, K.-H.: Lie Semigroups and Their Applications. Lecture Notes in Math, vol. 1552. Springer Verlag, Berlin, Heidelberg, New York (1993)

[24] Hilgert J, Neeb K-H (1995). Compression semigroups of open orbits on real flag manifolds. Monatshefte für Math..

[25] Hilgert J, Neeb K-H (2012). Structure and Geometry of Lie Groups.

[26] Hilgert J, Ólafsson G (1997). Causal Symmetric Spaces, Geometry and Harmonic Analysis, Perspectives in Mathematics.

[27] Kontou EA, Sanders K (2020). Energy conditions in general relativity and quantum field theory. Class. Quant. Grav..

[28] Krötz B, Neeb K-H (1996). On hyperbolic cones and mixed symmetric spaces. J. Lie Theory.

[29] Koeller J, Leichenauer S, Levine A, Shahbazi-Moghaddam A (2018). Local modular Hamiltonians from the quantum null energy condition. Phys. Rev. D.

[30] Lawson JD (1994). Polar and Ol’shanskiĭ decompositions. J. Reine Ang. Math..

[31] Longo R (1997). An analogue of the Kac-Wakimoto Formula and black hole conditional entropy. Commun. Math. Phys..

[32] Longo R (2019). Entropy of coherent excitations. Lett. Math. Phys..

[33] Longo R (2020). Entropy distribution of localised states. Commun. Math. Phys..

[34] Longo R, Morsella G (2023). The Massless Modular Hamiltonian. Commun. Math. Phys..

[35] Longo R, Feng X (2018). Relative entropy in CFT. Adv. Math..

[36] Loos O (1969). Symmetric Spaces I: General Theory.

[37] Loos O (1985). Charakterisierung symmetrischer R-Räume durch ihre Einheitsgitter. Math. Zeitschrift.

[38] Morinelli, V., Neeb, K.-H.: Covariant homogeneous nets of standard subspaces. Commun. Math. Phys. **386**, 305–358 (2021). arXiv:2010.07128

[39] Morinelli V, Neeb K-H (2022). A family of non-modular covariant AQFTs. Anal. Math. Phys..

[40] Morinelli, V., Neeb, K.-H.: From local nets to Euler elements, in preparation

[41] Morinelli V, Neeb K-H, Ólafsson G (2023). From Euler elements and $$3$$-gradings to non-compactly causal symmetric spaces. J. Lie Theory.

[42] Much, A., Passegger, A. G., Verch, R.: An approximate local modular quantum energy inequality in general quantum field theory, arXiv:2210.01145

[43] Morinelli V, Tanimoto Y, Wegener B (2022). Modular operator for null plane algebras in free fields. Commun. Math. Phys..

[44] Mund J (2001). The Bisognano-Wichmann theorem for massive theories. Ann. Henri Poincaré.

[45] Narnhofer H, Peter I, Thirring W (1996). How hot is the de Sitter space?, “Memorial Issue for H. Umezawa,” Internat. J. Mod. Phys. B.

[46] Neeb K-H (1991). Conal orders on homogeneous spaces. Inventiones Math..

[47] Neeb, K.-H.: Holomorphy and Convexity in Lie Theory. Expositions in Math. **28**, de Gruyter Verlag, Berlin, 2000

[48] Neeb K-H (2001). Compressions of infinite-dimensional bounded symmetric domains. Semigroup Forum.

[49] Neeb, K.-H., Ólafsson, G.: Antiunitary representations and modular theory. In: Grabowska, K., Grabowski, J., Fialowski, A., Neeb, K.-H. (eds) 50th Sophus Lie Seminar, Banach Center Publications **113**, 291–362 (2017) arXiv:1704.01336

[50] Neeb K-H, Ólafsson G (2021). Nets of standard subspaces on Lie groups. Adv. Math..

[51] Neeb K-H, Ólafsson G (2023). Wedge domains in compactly causal symmetric spaces. Int. Math. Res. Not..

[52] Neeb, K.-H., Ólafsson, G.: Wedge domains in non-compactly causal symmetric spaces. Geometriae Dedicata **217**(2), 30 (2023) arXiv:2205.07685 10.1007/s10455-023-09937-6 PMC10757968 38169487

[53] Neeb K-H, Ólafsson G, Ørsted B (2021). Standard subspaces of Hilbert spaces of holomorphic functions on tube domains. Commun. Math. Phys..

[54] Oeh D (2022). Nets of standard subspaces induced by unitary representations of admissible Lie groups. J. Lie Theory.

[55] Oeh D (2022). Classification of 3-graded causal subalgebras of real simple Lie algebras. Transf. Groups.

[56] Ólafsson G (1991). Symmetric spaces of hermitian type. Diff. Geom. Its Appl..

[57] Olshanski GI (1981). Invariant cones in Lie algebras, Lie semigroups and the holomorphic discrete series Function. Anal. Appl..

[58] Olshanski GI (1982). Convex cones in symmetric Lie algebras, Lie semigroups and invariant causal (order) structures on pseudo-Riemannian symmetric spaces. Soviet Math. Dokl..

[59] Roos, G., Jordan triple systems. In: Faraut, J. et al (eds.) Analysis and Geometry on Complex Homogeneous Domains, Progress in Math. **185**, Birkhäuser, Boston (2000)

[60] Satake, I.: Algebraic Structures of Symmetric Domains. Publ. Math. Soc. Japan **14**, Princeton Univ. Press (1980)

[61] Verch R (2000). The averaged null energy condition for general quantum field theories in two dimensions. J. Math. Phys..

[62] Wald RM (1984). General Relativity.

